# Retrofitting fiscal policies to reduce the double burden of malnutrition in Peru: A system dynamics modelling study

**DOI:** 10.1371/journal.pmed.1005167

**Published:** 2026-08-13

**Authors:** Nora A. Escher, Judite Gonçalves, Anibal Velasquez Valdivia, Paul Crosland, Luis Huicho, J. Jaime Miranda, Christopher Millett, Paraskevi Seferidi

**Affiliations:** 1 Public Health Policy Evaluation Unit, School of Public Health, Imperial College London, London, United Kingdom; 2 NOVA National School of Public Health, Public Health Research Centre, Comprehensive Health Research Center (CHRC), NOVA University Lisbon, Lisbon, Portugal; 3 Faculty of Public Health and Administration, Universidad Peruana Cayetano Heredia, Lima, Peru; 4 Youth Mental Health and Technology, Brain and Mind Centre, Faculty of Medicine and Health, Central Clinical School, University of Sydney, Sydney, Australia; 5 Department of Medicine, Faculty of Medicine, Universidad Peruana Cayetano Heredia, Lima, Peru; 6 Centro de Investigación en Salud Materna e Infantil, Universidad Peruana Cayetano Heredia, Lima, Peru; 7 Centro de Investigación para el Desarrollo Integral y Sostenible, Universidad Peruana Cayetano Heredia, Lima, Peru; 8 Sydney School of Public Health, Faculty of Medicine and Health, The University of Sydney, Sydney, New South Wales, Australia; 9 CRONICAS Centre of Excellence in Chronic Diseases, Universidad Peruana Cayetano Heredia, Lima, Peru; 10 Center for Research on Population and Health, Faculty of Health Sciences, American University of Beirut, Beirut, Lebanon; The Hospital for Sick Children, CANADA

## Abstract

**Background:**

Peru faces a double burden of malnutrition, characterised by the co-existence of overnutrition and undernutrition. Although several fiscal policies have been implemented to address nutrition challenges, these have been designed and implemented separately, limiting their potential for synergistic impact. This paper investigates the potential impact of retrofitting unhealthy food and beverage taxation for ‘double-duty’ action by allocating revenues towards welfare transfers and fruit and vegetable subsidies on overweight and stunting in Peru.

**Methods and findings:**

We developed a system dynamics model to simulate overweight and stunting in children and adults of reproductive age, using secondary data sources and published effect estimates. We modelled several fiscal policy scenarios, informed by WHO recommendations and Peru’s policy landscape. We performed several tests to build confidence in the model. Fiscal policy scenarios can have beneficial effects on overweight compared to business-as-usual, mainly through the impact of additional unhealthy food and beverage taxes, which would reduce cumulative person-years lived with adult overweight between 2024 and 2040 by −58.6 (95% Uncertainty Interval [UI] [−77.7, −37.9]) million, mainly among the richest. Reinvesting tax revenue to increase the amount of cash offered through Peru’s existing conditional cash transfer could reduce cumulative person-years lived with child stunting by up to 876 thousand (95% UI [−1,139, −567]) by 2040, with 82% of these being among the poorest. This study relies on several assumptions regarding policy impacts and responses and does not incorporate cost-effectiveness analysis due to its exclusion of older age groups.

**Conclusions:**

Retrofitting unhealthy food and beverage taxation to reallocate revenue towards targeted welfare programmes, such as cash transfers, has substantial potential for ‘double-duty’ impacts on overnutrition and undernutrition in Peru.

## Author summary

**Why was this study done?:**

- Peru faces a double burden of malnutrition (DBM), with high levels of both overweight and stunting.
- The World Health Organization (WHO) has called for double-duty actions to address the DBM, including by retrofitting existing interventions to concurrently address multiple forms of malnutrition.
- The WHO recommends fiscal policies as an effective way to improve diets, but their potential as double-duty actions has not been previously explored.

**What did the researchers do and find?:**

- We developed a system dynamics model that simulates overweight and stunting trends in children and adults of reproductive age in Peru between 2000 and 2040 across two socioeconomic groups.
- We developed policy scenarios informed by two of Peru’s flagship fiscal policies with strong potential to reduce overweight and stunting, its sugar-sweetened beverage tax and its conditional cash transfer Juntos, as well as existing fiscal policy recommendations for healthier diets.
- We modelled a total of eight scenarios combining two tax options on unhealthy foods and beverages with four ways of reallocating the resulting revenue by expanding or increasing Juntos transfers, expanding the Juntos Early Childhood Transfer, or funding a fruit and vegetable subsidy.
- Retrofitting Peru’s current fiscal policies by expanding its current sugar-sweetened beverage tax and reinvesting its revenue to increase the amount of cash offered through Peru’s existing conditional cash transfer programme has strong potential to reduce total rates of overweight and stunting among the poorest by 2040.

**What do these findings mean?:**

- Retrofitting fiscal policies to incorporate double-duty objectives by reallocating revenues from unhealthy food and beverage taxes towards social safety nets holds significant promise for addressing the DBM in Peru. These benefits are maximised when revenues are strategically reinvested in programmes targeting the poorest.
- Future modelling studies should examine the health impacts and cost‑effectiveness of reallocating revenues from unhealthy food and beverage taxes across different strategies to address the diverse forms of the DBM.
- This study relies on several assumptions regarding policy impacts, market growth, and industry responses and does not incorporate cost-effectiveness analysis due to its exclusion of older age groups.

## Abstract 3

In this modelling study, Nora Escher and colleagues investigate whether retrofitting policies, to allocate taxes from unhealthy food taxes towards healthy food policies, can improve the double burden of overnutrition and undernutrition in Peru.

## Introduction

Malnutrition in all its forms affects nearly one-third of the global population, posing a major health challenge and a barrier to achieving the United Nations’ (UN) Sustainable Development Goals (SDGs) [1,2]. Globally, over 820 million people still lack sufficient food, while many others consume poor-quality diets, leading to micronutrient deficiencies, rising obesity, and diet-related noncommunicable diseases (NCDs) [3]. In many low- and middle-income countries (LMICs), the growing shift toward diets high in ultra-processed foods (UPFs) and sugar-sweetened beverages (SSBs) has resulted in a double burden of malnutrition (DBM), where undernutrition and overnutrition coexist within populations, households, and individuals [4–7]. This phenomenon is exemplified in Peru, which has faced a significant increase in overweight and obesity over the last decades, with 65% of adults classified as overweight in 2022 [6,8]. At the same time, Peru has a long history of stunting, and despite significant reductions since the 2000s, recent data indicate that progress has stagnated over the last 5 years [4]. These trends have led to DBM at both household and population levels in Peru, with persistent inequalities [5,6].

In response, the World Health Organization (WHO) has called for double-duty actions, i.e., interventions that tackle both undernutrition and overnutrition simultaneously by targeting their shared drivers [9]. Fiscal policies, including taxes on SSBs and unhealthy foods, subsidies for nutritious foods, and social assistance programmes, have enormous potential as double-duty actions [10]. These policies, usually implemented in silos, can be retrofitted to reduce both overnutrition and undernutrition through revenue reallocation, as recommended by WHO [11]. Revenue reallocation can also build public support through compensation of poorer groups and greater transparency in how revenues are spent [11–15]. While more than 110 countries worldwide have introduced taxes on SSBs [16], and some on UPFs [17], earmarking of revenues remains limited and has primarily focussed on addressing overnutrition [18]. The potential to use food tax revenue to fund double-duty actions, and the optimal combination of policies to address both undernutrition and overnutrition in different country contexts remains largely unexplored.

Peru has implemented fiscal policies to address undernutrition and overnutrition, but these have been designed and implemented separately with no policies explicitly designed or implemented as double-duty actions [19–23]. This primarily stems from the division of responsibility across government departments, with overnutrition overseen by the Ministry of Health, whilst undernutrition falls under social policy programmes led by the Ministry of Development and Social Inclusion. We focus on the *Juntos* cash transfer program (2005) and a tiered SSB tax (2018) as examples of policies implemented in the country with potential to be retrofitted as double-duty actions. Given robust national or international evidence of their independent impacts [22,24–30] and recent reforms, such as the Early Childhood Transfer of Juntos, these policies are both evidence-based and timely options for exploring double-duty potential of fiscal policies in Peru.

*Juntos* was developed to tackle poverty, especially in rural areas, but has since been credited with contributing to the reduction of under five stunting rates in Peru, which declined nationally from 31.3% in 2000 to 12.2% in 2019 [4,31]. *Juntos* initially provided 100 soles monthly (~22% of the minimum wage then) to households achieving health and education conditionalities, under the Basic Transfer (*Transferencia Base*). In recent years, additional transfers were introduced, such as the Transfer for Early Childhood (*Transferencia primera infancia)* in 2021, which added 50 soles for households with children under five [26]. Peru’s tiered SSB tax was introduced in 2018 in response to the country’s increasing obesity rates. It applies a 25% tax rate to beverages with more than 6 g of sugar per 100 mL and 17% to lower-sugar options [32]. Unlike Mexico and Colombia, which implemented broader junk food taxes, Peru has not yet extended taxes to unhealthy foods [33,34]. Moreover, Peru does not earmark revenue from its SSB tax for any specific health or nutrition programmes. Although the impacts of the Peruvian SSB tax on overweight and obesity have not yet been evaluated, substantial modelling and observational evidence from other settings, including in Latin America, indicate its strong potential to reduce SSB intake and associated overweight and obesity prevalence [21–23].

The WHO emphasises the potential of retrofitting existing interventions as a viable mechanism for creating double-duty actions [9]. It identifies revenue reallocation as an important mechanism to enhance synergistic impacts of fiscal policies [11]. Although previous research has extensively examined the impact of SSB taxes on dietary intake and overnutrition outcomes [21,22,35], very few studies have explored revenue-earmarking strategies, and those that did focussed on limited scenarios primarily targeting overnutrition [36]. No prior studies have explicitly examined the potential of retrofitting existing nutrition‑relevant fiscal policies, such as Peru’s SSB tax and Juntos, to jointly target the DBM. In this study, we investigated the role of fiscal policies on the DBM in Peru by modelling the potential impact of retrofitting unhealthy food and beverage taxation for double-duty objectives through revenue reallocation, expanding conditional cash transfer programmes and fruit and vegetable subsidies in Peru. We employed a system dynamics approach to estimate impacts of various tax and reallocation scenarios on national stunting and overweight across socioeconomic groups.

## Methods

### System dynamics modelling

System dynamics modelling is a differential equation-based simulation modelling approach concerned with explaining dynamic behaviour, i.e., how problems change over time [37]. It is grounded on three main principles: feedback, accumulations, and delays. Feedback denotes situations in which an effect generates an outcome that, in turn, influences its original cause, creating causal loops. Accumulations, or stocks, refer to the gradual change of a system state, arising when rates of decrease and increase differ over time. Finally, delays capture the phenomenon where the effect of one variable on another does not occur instantaneously, but after a temporal lag [37].

These characteristics constitute system dynamics as a highly suitable approach for this study. First, the capacity of system dynamics to capture feedback mechanisms enables modelling of the intergenerational transmission of nutrition outcomes, from children to adults and back to children. Previous modelling analysis of the DBM in Peru highlights this important gap and recommends that future modelling work should incorporate the intergenerational transmission of overnutrition and undernutrition [38]. Second, the modelling of accumulations and delays allows us to capture important temporal lags that often characterise changes in prices and market conditions. There are limited studies that employ system dynamics models to explore the dynamics and impacts of SSB taxation [39]. Only one prior study estimated the impact of SSB taxation on body weight via changes in dietary energy intake [40], with no exploration of both overnutrition and undernutrition outcomes or double-duty scenarios. Finally, given our focus on aggregated population-level dynamics, system dynamics modelling offers a more suitable methodological approach compared to other micro-perspective modelling frameworks [41].

### Overview of modelling process

We developed a system dynamics model in Vensim DSS 10.1.3 in two iterative steps. First, we developed the model’s population component, comprising a co-flow structure tracking three ageing chains of Peru’s overall, stunted, and overweight populations, based on Sterman’s population dynamics formulations [37] and building on our previous modelling work on the DBM [42]. We then developed the model’s policy component, comprising three main market stock and flow structures, representing the market size of SSBs, UPFs, and fruit and vegetables, as well as a fourth stock and flow structure, representing tax revenue accumulation. We manually calibrated the model to reproduce historical trends of adult overweight and under-5 stunting, using data from Peru’s Demographic and Health Surveys (2000–2020), and historical market size data from Euromonitor (2000–2024) [43], evaluating quality of fit by visually comparing simulated outputs with corresponding reference modes. A causal loop diagram depicting a summarised overview of the model structure is presented in S1 Fig. Detailed equations of stock and flow formulations are presented in S4 Text. The modelling process is described in more detail below.

### Population model development

We developed a system dynamics model to simulate overweight (including both overweight and obesity) and stunting trends in children and adults of reproductive age in Peru between 2000 and 2040. This time horizon allowed capturing intergenerational impacts of DBM, while also utilising existing shorter-term evidence to support our assumptions. The model simulates overall, stunted, and overweight populations as they age over time, from birth to adulthood (0–49 years). Persons aged 50+ years were not included due to lack of anthropometric data in Peru. The model also captures the intergenerational transmission of stunting through low birth weight. It is disaggregated into six age (0–4, 5–9, 10–14, 15–24, 25–34, and 35–49) and two socioeconomic status groups (poorest quintile and remaining 80%). The model was populated using data from secondary data sources, including the World Bank and Peru’s Demographic and Health Surveys and meta-analyses of longitudinal studies [44–47]. Further details on population model structure and data sources are provided in S1 and S2 Texts.

### Policy selection

**Fig 1:**
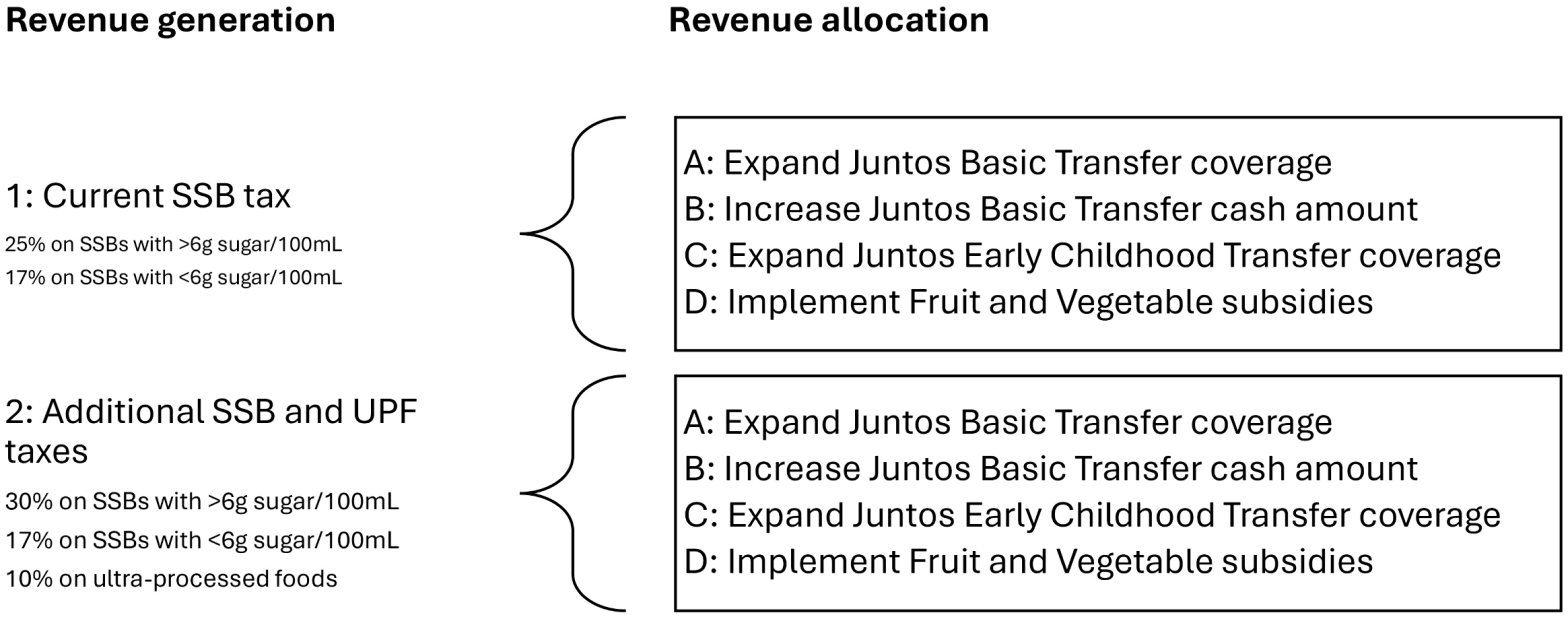
Overview of modelled scenarios showing revenue generation and allocation options. Different policy scenarios for revenue allocation are further described in S2 Table. SSB: sugar-sweetened beverages; UPF: ultra-processed food.

Selection of fiscal policies was informed by WHO recommendations identifying unhealthy food and beverage taxation and revenue-earmarking to fruit and vegetable subsidies as key levers to promote healthier diets [11], as well as increasing calls for food environment policies, such as SSB taxation, to act as double-duty actions [10]. In total, we simulated eight combined policy scenarios, resulting from the combinations of two revenue generation scenarios and four revenue allocation scenarios (Fig 1).

Revenue generation was estimated from unhealthy food and beverage taxes. Specifically, the first scenario modelled the current SSB tax (25% for beverages with >6 g/100 mL of sugar and 17% for those with <6 g/100 mL), and the second scenario modelled an increased SSB tax (30% on beverages with >6 g/100 mL of sugar) combined with a new 10% UPF tax, following Colombia’s example in the implementation of UPF taxation [34]. Further details on policy scenarios are provided in S2 Table.

Revenue allocation scenarios reinvested money generated by each taxation option into A) Expanding the Basic Transfer of the *Juntos* programme to more people, B) Increasing the amount of the Basic Transfer of the Juntos programme, C) Expanding the Early Childhood Transfer of the Juntos programme to more people, or D) Financing a Fruit and Vegetables subsidy. Scenarios A and B were selected in accordance with SDG 1.3, reflecting the goals of ensuring both adequate and sufficient coverage of social assistance, while Scenario C reflects recent changes to the Juntos programme. Scenario D follows WHO recommendations for revenue reallocation to promote healthier diets [11].

### Modelling revenue generation and allocation

We estimated tax revenue under each tax scenario by applying the respective tax rates to modelled market sizes for UPFs and SSBs. We modelled UPF and SSB market sizes using annual per capita sales data from Euromonitor (in Peruvian Nuevos Soles, S/) [43], assuming uniformity across socioeconomic groups, given lack of stratified data. We used previously published data on pre-tax SSB purchases in Peru [32] to estimate the proportion of the SSB market that fell within the < 6 g sugar/100 mL and >6 g sugar/100 mL groups. We projected growth rates in SSB and UPF market sizes to align with the Latin American average by 2040. In Peru, similarly to other Latin American countries, UPF and SSB sales more than doubled between 2010 and 2024 [43,48].

Estimated tax revenue was distributed to each of the four revenue allocation scenarios to estimate the maximum potential impact under an idealised full earmarking assumption. For *Juntos* coverage expansions (allocation scenarios A and C), the poorer quintile had priority over wealthier quintiles. After achieving full coverage at 100%, excess revenue was left unspent. For Juntos cash amount increase (allocation scenario B), we estimated the cash increase that the accumulated tax revenue could achieve per household per year. For fruit and vegetable subsidies (allocation scenario D), we calculated the flat, population-wide subsidy rate that estimated SSB/UPF tax revenue could fund, using annual fruit and vegetable sales data from Euromonitor [43].

### Modelling policy impacts

We modelled the effect of policies on stunting and overweight prevalence and person-years between 2024 and 2040. Prevalence was estimated as the number of people with stunting and overweight divided by the total number of people in each age and socioeconomic group. Person-years were estimated as the cumulative number of years lived with stunting and overweight between 2024 and 2040 in each age and socioeconomic group. Before 2024, we assumed that changes in transition probabilities informed by epidemiological data already captured any potential effects of existing policies, including the SSB tax (since 2018) and the *Juntos* programme (since 2005); therefore, no additional policy impacts were modelled. From 2024, we estimated the impact of different policy scenarios on stunting and overweight using effect estimates derived from previous studies (S3 Table), prioritising primary studies from Peru. When these were not available, we used meta-analyses of Latin American studies, meta-analyses of LMIC-based studies, primary studies from Latin American countries, meta-analyses of high-income countries studies, and primary studies from high-income countries, in this order of priority.

#### Effect of SSB and UPF taxes on overweight.

To model the effect of additional taxation (a 5 percentage point increase in the SSB tax and a new 10% UPF tax) on overweight in children and adults, we used published estimates of own-price and cross-price elasticities for UPF and SSB demand to simulate changes in market size (S3 Table). Elasticity effects were assumed to gradually increase, peaking after 2 years to reflect an adjustment period, and then lessening over 7 years until dissipating completely, consistent with prior research [29,49]. We then linked country-level changes in SSB consumption to overweight prevalence using estimates from a multi-country study [28]. For UPFs, comparable data were unavailable, so we applied the same values as for SSBs. Due to lack of relevant data, we assumed uniform SSB and UPF consumption across income groups.

#### Effect of Juntos policies.

We modelled the effect of different *Juntos* scenarios on both stunting and overweight in children only.

In **policy scenario A**, we modelled expansion of coverage of the *Juntos Basic Transfer*. Using the latest data on the number of households that received the *Juntos Basic Transfer* and the average number of children per household, we estimated the coverage expansion achievable through the modelled tax revenue generation. Since all children in the low socioeconomic group were already covered under the current *Juntos* scheme, this scenario modelled coverage expansion of the *Basic Transfer* to children in the high socioeconomic group. We modelled the effects of *Juntos* on stunting and overweight in children using data from primary studies [24,25]. In the main analysis, we modelled no effects in adults, due to inconsistent findings in available literature [25]. We further conducted a sensitivity analysis assuming an effect on adult overweight equal to that for child overweight.

In **policy scenario B**, we modelled the impact of increasing the cash value provided to current beneficiaries of the *Juntos Basic Transfer*. Due to a lack of Peruvian studies on the effect of a cash transfer increase, we relied on estimates from Mexico’s conditional cash transfer programme [24,27]. Given mixed findings of Peruvian studies on the impacts of conditional cash transfers on adult overweight, we again only modelled effects of cash transfer increases on stunting and overweight in children in our main analysis [25]. We further conducted a sensitivity analysis assuming an effect on adult overweight equal to that for child overweight.

In **policy scenario C**, we simulated the impact of expanding coverage of the *Juntos Early Childhood Transfer*, using data on programme participation and its effect on stunting [26]. Due to data availability, the modelled effect was restricted to stunting in children under five, with no effects modelled on childhood overweight or adults living in recipient households. This scenario modelled expanding coverage of this transfer to all children under five in the lowest wealth quintile, before expanding to the upper quintiles. We adjusted the impact of *Juntos* scenarios on overweight and stunting using an annually varying discount rate, recognising that the purchasing power of cash transfers declines over time [50].

#### Effect of fruit and vegetable subsidies.

In **policy scenario D,** we modelled the impact of a reduction in the price of fruit and vegetables on stunting in children and overweight in children and adults. We used data from a multi-country study linking food prices to national stunting prevalence across 176 countries [51], and a longitudinal study from the U.S., which estimated the impact of a 10% price increase in fruit and vegetables on overweight [52]. In the lowest wealth quintile, we adjusted the effect of the price change to the proportion of people in that quintile meeting fruit and vegetable dietary requirements, using published fruit and vegetable consumption estimates [53]. Due to limited evidence on the effects of fruit and vegetable subsidies over time, we assumed that, similar to tax scenarios, the impact gradually increases, reaches its peak 2 years after implementation, and then tapers off, with effects diminishing after 7 years [29,49].

### Model testing

Following recommendations by Sterman [37], we conducted several model robustness assessments. First, we conducted behaviour reproduction tests to visually evaluate our model’s ability to replicate observed trends (S3 Text). Second, we ensured that all model variables have a clear real-life meaning, with consistency in units of measurement (S3 Table and S4 Text). Third, we conducted deterministic sensitivity analyses by varying all exogenous (data-informed) model parameters by ±10% (S5 Text). Fourth, we conducted extreme condition testing, assessing whether the model produced expected behaviour under extreme scenarios, such as extreme fertility and mortality rates (S6 Text). Finally, we compared the model’s behaviour under different integration methods and time steps (S7 Text) [16]. Separate robustness tests were conducted for the population model without any policies, and for the model including policy effects.

### Probabilistic sensitivity analysis

We incorporated uncertainty in effect estimates through probabilistic sensitivity analysis. We employed Monte Carlo simulations to draw random values from normal distributions parameterised for selected effect estimates, price elasticities, and transition probabilities used in the model (see S4 Table). We then estimated the median and 2.5 and 97.5 percentiles of 800 model iterations to produce final point estimates and 95% uncertainty intervals (95% UIs) for adult overweight and child stunting prevalence and cumulative person-years.

AI tools were not used to generate any article content and were only used for minor editorial support (grammar, syntax, and clarity checks) in limited, selected parts of the text.

## Results

The model reproduced observed trends in overweight and stunting overall and by socioeconomic groups until 2020 (S3 Text). In a business-as-usual scenario, it estimated that overweight rates would continue to gradually rise, reaching ~83.1% in adults by 2040, whilst stunting in children would continue its trend, plateauing at ~6.1% in the last 10 years of the simulation.

### Tax revenue generation

**Fig 2:**
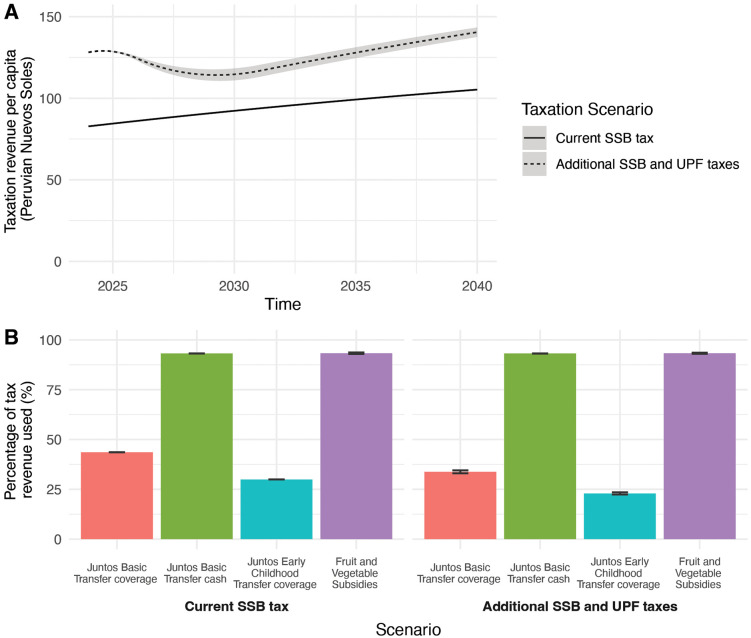
Estimated revenue generation and reallocation under modelled scenarios. **(A)** Revenue generated by the current SSB tax (25% on SSBs with ≥6 g/100 ml sugar) and the additional SSB and UPF taxes (30% on SSBs with ≥6 g/100 ml sugar and 10% on UPFs). **(B)** Percentage of tax revenue used in each reallocation scenario for the current SSB tax and the additional SSB and UPF taxes by the end of the simulation period (2040). Shaded areas and error bars represent 95% Uncertainty Intervals (95% UI). SSB: sugar-sweetened beverages; UPF: ultra-processed food.

Our model estimated that maintaining the current SSB tax could yield on average over S/2.4 billion or S/94.8 per capita each year, with revenues expected to grow over time due to market expansion (Fig 2A), accumulating a total of almost S/39 billion during 2024–2040. In comparison, additional SSB and UPF taxes were projected to generate on average more than S/3.2 billion or/S125.1 per capita each year. A temporary revenue dip may occur after the first year of implementation, due to the taxes’ effects on purchasing behaviour, but the model predicted annual revenue recovery within 5 years, followed by sustained long-term growth (Fig 2A), totalling ~S/51 billion between 2024 and 2040.

### Tax revenue reallocation

Under Scenario A, estimated revenue from both taxation scenarios would allow a 100% coverage of the *Juntos Basic Transfer* among households with children under 15 (S2A Fig), using no more than 44% of the generated revenue from the current SSB tax and 34% of the additional SSB/UPF taxes by 2040 (Fig 2B). Scenario B would enable an increase of over 300% and 400% in *Basic Transfer* amounts for current recipients, using revenue generated under the current SSB and additional SSB/UPF taxes, respectively (S2B Fig). Scenario C would achieve full coverage of the *Early Childhood Transfer* for households with children under 5 (S2C Fig), using only 30% and 21% of revenue generated under the current SSB and additional SSB/UPF taxes, respectively (Fig 2B). Finally, revenue under the current SSB or additional SSB/UPF tax scenarios could fund up to a 17% or a 23% population-wide subsidy for fruits and vegetables, respectively (S2D Fig), under Scenario D.

### Scenario impacts on overweight and stunting

**Fig 3:**
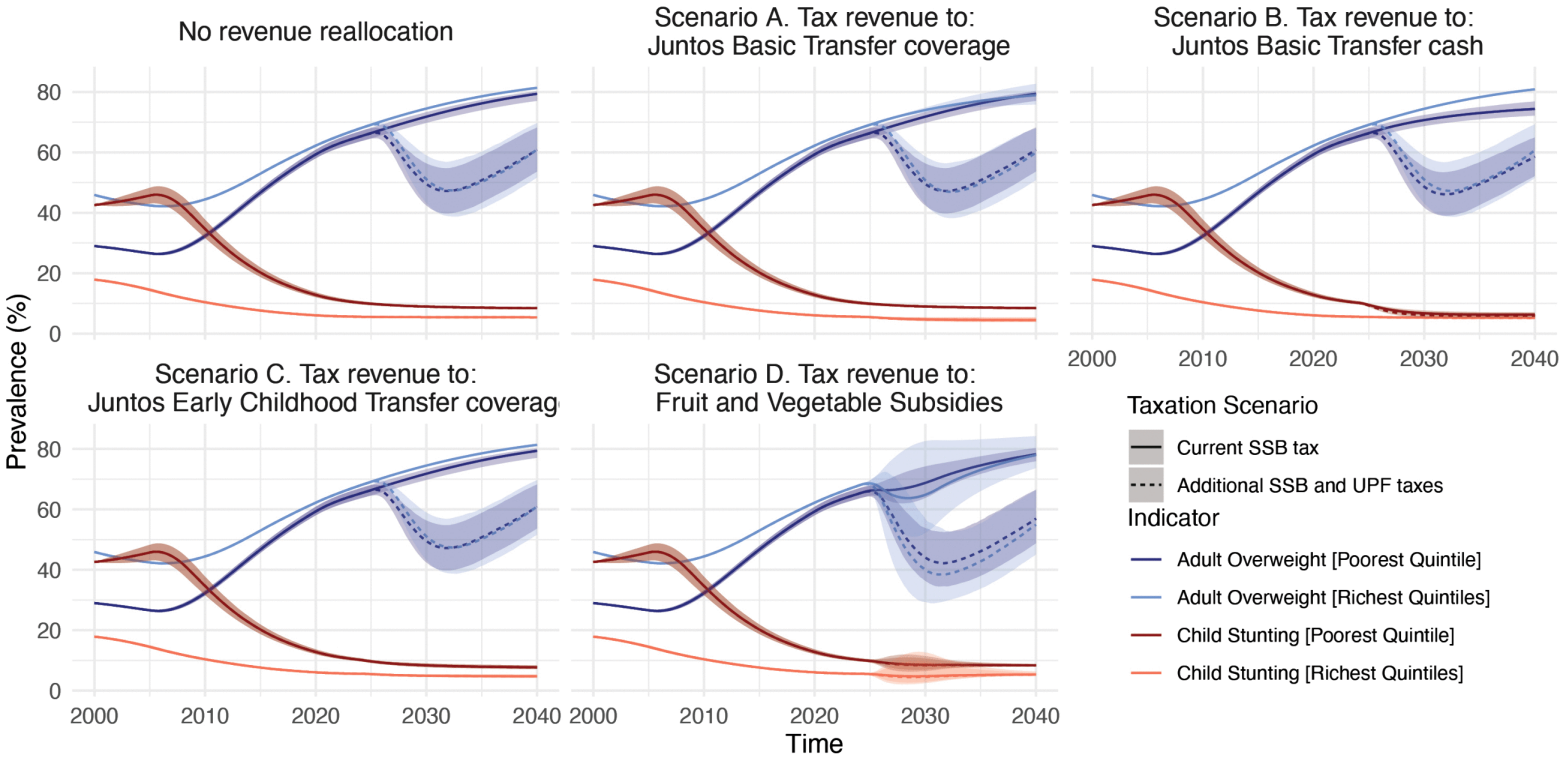
Modelled effects of policy scenarios on child stunting and adult overweight prevalence by socioeconomic group. Lines represent point estimates and shaded area represents 95% Uncertainty Intervals (95% UIs). Dashed lines represent scenarios with revenue generated by the current SSB tax, while dotted lines represent scenarios with revenue generated from additional SSB and UPF taxes. Each panel shows a different revenue reallocation scenario. SSB: sugar-sweetened beverages; UPF: ultra-processed food.

**Fig 4:**
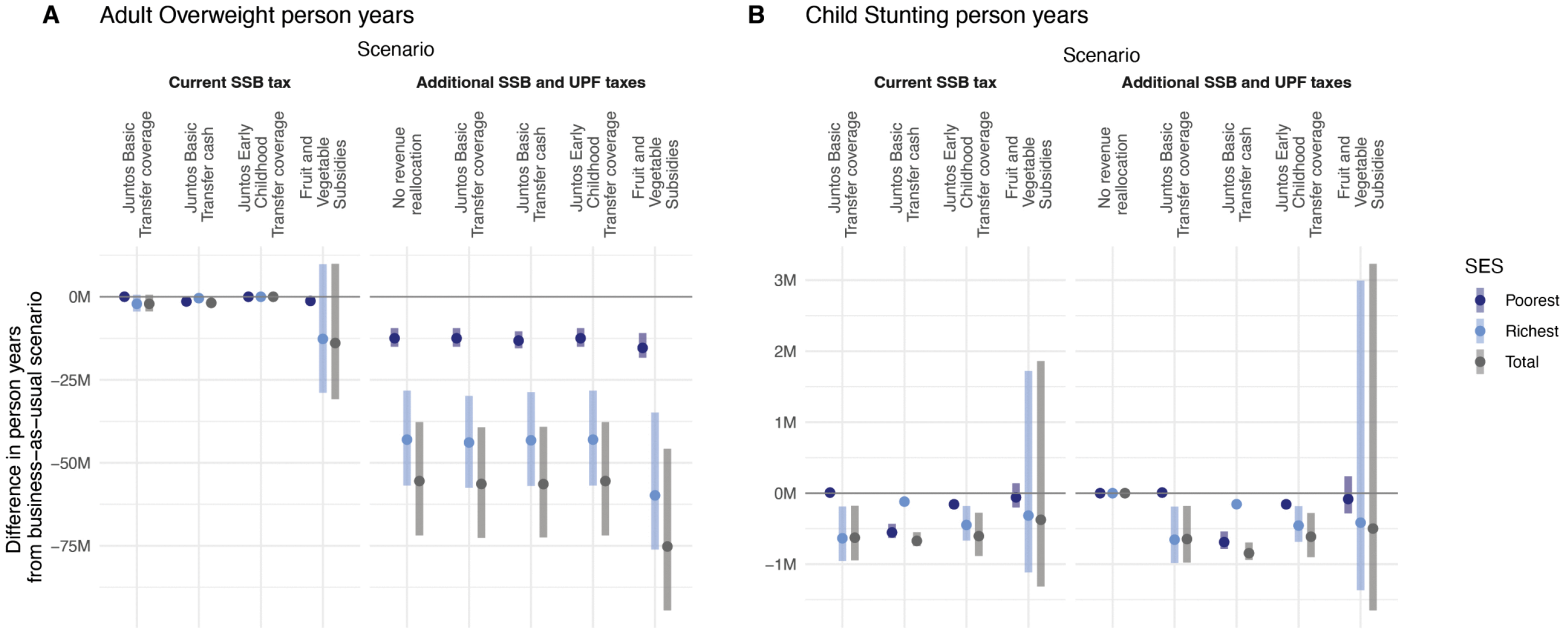
Difference in cumulative person-years lived with overweight in adults of reproductive age and stunting in children by 2040 between business-as-usual and modelled scenarios, overall and by socioeconomic group. Error bars represent 95% Uncertainty Intervals. SSB: sugar-sweetened beverages; UPF: ultra-processed food; SES: socioeconomic status.

Figure 3 shows estimated trends in adult overweight and childhood stunting prevalence across scenarios, while Fig 4 presents reductions in cumulative person-years lived with these outcomes between 2024 and 2040, compared to business-as-usual. Expansion in the coverage of the *Juntos Basic Transfer* (Scenario A) would mainly benefit overweight and stunting among the higher socioeconomic groups only, as all lower-income households were already receiving this transfer. The model suggested a reduction of 658 (95% UI [−1,128, −45]) thousand child stunting person-years and 59.69 (95% UI [−78.4, −39.5]) million adult overweight person-years under the additional SSB/UPF tax scenario compared to business-as-usual, with the latter mainly attributed to the effect of the taxes on SSB and UPF consumption, which alone would reduce cumulative person-years lived with adult overweight by 58.6 (95% UI [−77.7, −37.9]) million. In contrast, increasing the Juntos Basic Transfer cash amount (Scenario B), would predominantly benefit those in the lowest quintile, who are existing beneficiaries. Effects on stunting would be the greatest under this scenario, leading to a reduction in cumulative person-years lived with child stunting by up to 876 thousand (95% UI [−1,139, −567]), with 82% of these being among the poorest, although inflation may lead to a plateauing effect over time. Simulated reductions in childhood overweight would eventually reduce adult overweight as children age, saving an additional 59.6 (95% UI [−78.4, −39.5]) million adult overweight person-years under the additional SSB/UPF tax scenario compared to business-as-usual. Modelled expansion of the *Early Childhood Transfer* coverage (Scenario C) would not incur any additional impact on overweight beyond that of the taxes, although a reduction of 621 (95% UI [−1,098, −94]) thousand child stunting person-years under the additional SSB/UPF scenario compared to business-as-usual was estimated, with ~26% of these occurring among the poorest. Finally, modelled fruit and vegetable subsidies (Scenario D) would result in a small initial decrease in stunting and overweight, followed by a gradual rebound, as effectiveness of subsidies diminished over time. However, UIs were wide due to uncertainty in the effect estimates of fruit and vegetable subsidies on overweight and stunting. Sensitivity analysis assuming that expansion of coverage and cash amount of Juntos also benefits overweight in adults showed an additional reduction of maximum of 2.1 (95% UI [−2.4, −1.3]) and 1.6 (95% UI [−1.3, −1.9]) million overweight person-years among adults under the additional SSB/UPF Scenarios A and B, respectively (S5 Table).

### Model testing

Our model reproduced observed trends in overweight and stunting overall and by socioeconomic groups well (S3 Text). Sensitivity analyses showed that most ±10% parameter changes caused only minor numerical deviations (<5%) and no changes in the pattern of modelled trends (S5 Text). Overweight prevalence was most sensitive to changes in UPF and SSB market size parameters, including those impacting the rate of market size growth, while stunting prevalence was minimally impacted by variations in policy parameters and more sensitive to population model factors, such as baseline values of infant stunting and stunting transition probabilities. Extreme-value testing validated the model’s logical consistency, showing predictable responses to variations in mortality, fertility, market growth, and tax rates (S6 Text). Comparison of different integration methods and time steps showed consistency in simulations (S7 Text).

## Discussion

This study demonstrates that retrofitting fiscal policies as dual benefit actions has the potential to reduce both overnutrition and undernutrition in Peru. Specifically, expanding existing SSB taxes and directing revenues to welfare transfers could reduce stunting and overweight, with varying impacts across socioeconomic groups. Estimated benefits of policy scenarios on overweight in both socioeconomic groups were mainly attributed to SSB and UPF taxes, despite market feedback mechanisms reducing their effectiveness over time (S1 Fig, loop B: SSB market and loop B: UPF market). Using tax revenue to raise the amount of conditional cash transfers for current recipients of the *Juntos Basic Transfer* appears particularly effective in reducing stunting among the poorest, where stunting prevalence is highest, dominating the impact of the reinforcing feedback loop driving the intergenerational transmission of stunting (S1 Fig, loop R: Intergenerational transmission of stunting). Conversely, fruit and vegetable subsidies could moderately reduce overweight, though estimates had wide confidence intervals, and benefits would disproportionately favour wealthier groups, with limited and uncertain impact on stunting among the poorest.

Our model suggests that SSB/UPF taxes can reduce overweight across socioeconomic groups in the short-term, consistent with previous literature [54]. Evidence regarding the long-term effects of such taxes remains limited, although recent data from Hungary support our estimated fade-out effect of modelled SSB/UPF taxes on overweight [49]. Our findings show that deploying tax revenues to increase the *Juntos Basic Transfer* amount would reduce stunting in the poorest quintile. The *Basic Transfer* benefit has not been indexed to inflation since programme inception in 2005, contrary to World Bank recommendations, which may partially explain the increase in stunting in Peru in recent years [55]. While expanding coverage of both *Juntos Basic Transfer* and *Early Childhood Transfer* showed similar effects on childhood stunting, the latter would be particularly effective in reducing under five stunting, especially among the poorest. This highlights its importance as an early nutrition intervention, given the extensive literature on the role of early life nutrition in mitigating the long-term health consequences of stunting [56–58]. Simulations show that fruit and vegetable subsidies temporarily reduce overweight in wealthier groups, given their higher baseline consumption of fruit and vegetables [53]. This is consistent with evidence suggesting that price cuts of 10–30% for fruit and vegetables increased intake, but demand was inelastic among poorer groups [59,60].

Our research has several strengths. First, it deploys a novel, population-based model that simulates the effects of fiscal policies on the DBM, incorporating demographic trends in Peru, intergenerational transmission of stunting, and socioeconomic group stratification. Second, selected policies are based on existing initiatives, enhancing their practical and political applicability. Third, there is growing recognition of the need for models that can simultaneously assess impacts on stunting and overweight to better evaluate and compare policies [61]. While this model did not aim to provide forecasts or precise estimates of effect size, it offers valuable insights into the effects of different what-if scenarios on policy budgeting and DBM outcomes.

This study also has several limitations. First, it relies on several simplified assumptions regarding *Juntos* programme impacts, SSB and UPF market growth, tax and subsidy impact fade-out, and industry responses, such as product reformulation following taxes. While such assumptions are inherent to modelling, sensitivity analyses and extreme value testing were conducted to assess robustness. Second, due to limited Peru-specific data, the study relies on some international estimates, which may not fully reflect the local context and could affect simulation accuracy. Third, although the model captured socioeconomic inequalities through stratification into two socioeconomic groups, we did not examine more granular disaggregation by wealth or consider other factors of inequality, such as geographic region. Fourth, our study does not model all potential double-duty actions in Peru but rather focuses on flagship fiscal policies that have strong DBM potential due to existing evidence of their impacts on nutrition outcomes [22,24–30]. Future research should explore the impact of other policy options with strong double-duty potential, including breastfeeding promotion and healthcare interventions targeting infectious disease burden. Similarly, our study focuses on overweight/obesity and stunting as DBM indicators, although Peru faces high levels of several additional overnutrition, such as diabetes, as well as undernutrition outcomes, such as anaemia. Future research should explore additional outcomes, particularly as new evidence on policy impacts emerges. Finally, we did not conduct cost-effectiveness analysis, as summary measures of population health needed for this analysis, such as disability-adjusted life years, would have been underestimated in this model due to its age group coverage, which does not include people over 49, excluding age groups where overweight-attributable measures are the highest.

Our findings highlight several policy design priorities for retrofitting fiscal policies in Peru to achieve double-duty objectives. First, cash transfers, such as those provided through *Juntos,* represent a straightforward mechanism for reinvesting tax revenues by leveraging existing infrastructure and align with targets related to prioritising vulnerable populations, outlined in Peru’s National Development Strategic Plan to 2050 [62]. Increasing the *Basic Transfer* amount should be prioritised, as it appears to have potential to reduce stunting among the poorest households. Second, targeted transfers to families with young children offer a relatively low-cost approach to addressing stunting during a critical developmental stage. With ~100,000 households currently receiving these transfers in Peru, there is potential for further expansion among the lowest socioeconomic group. Third, targeted subsidies (e.g., through healthy food vouchers) to enhance fruit and vegetable consumption among low-income populations could promote greater health equity. This directly responds to priority L1.1 of Peru’s National Multisectoral Health Policy to 2030 to improve healthy population habits and behaviours [63]. However, such interventions face implementation challenges, and further research is necessary to assess their feasibility and potential impact. Fourth, the model simulated a declining impact of tax and subsidy policies, driven by consumer adaptation, highlighting the need for complementary policies and continuous monitoring, evaluation, and adaptation, to sustain the long-term impact of fiscal interventions. Following recommendations related to policy monitoring and evaluation, outlined in the National Multisectoral Health Policy [63], can respond to these needs. Behavioural change initiatives, such as school-based programmes promoting healthy habits, as well as food environment modifications, could reinforce and amplify the benefits of tax and subsidy measures, ensuring a more lasting impact on malnutrition outcomes [64], whilst enhancing the impact of conditional cash transfers by discouraging spending on unhealthy foods. Ultimately, these recommendations can be achieved by capitalising on existing intersectoral platforms, such as those that enabled recent cross‑departmental health and development guidelines [62,63], and strengthening collaboration with civil society actors who have been instrumental in implementing relevant policies in Peru, including the SSB tax. At the same time, persistent barriers need to be overcome, including weak alignment of shared policy goals across ministries and sustaining long‑term funding, especially amid periods of political instability.

In conclusion, retrofitting fiscal policies to incorporate double-duty objectives by reallocating revenues towards social safety nets holds significant promise for addressing the DBM in Peru, particularly when revenues are strategically reinvested in programmes targeting the poorest, with the potential to generate both immediate and long-term health and economic impacts. Our findings demonstrate how system dynamics approaches can advance academic understanding of the DBM by capturing intergenerational pathways, and accounting for over‑time effects such as revenue accumulation and market adaptation, elements largely overlooked in previous modelling studies. Directly linking revenue streams from taxes on unhealthy foods and beverages to undernutrition interventions aligns with WHO recommendations for fiscal policies to promote healthier diets while offering double-duty potential [11]. While taxes on SSBs and UPFs present equity challenges, their progressive health impacts, especially when paired with earmarked revenues for targeted welfare programmes or subsidies, could make them valuable tools in addressing the DBM. Integrating fiscal policies can address both immediate and systemic DBM drivers, such as poor diets and poverty, while supporting progress on key SDGs, including poverty reduction (SDG 1), zero hunger (SDG 2), and improved health and well-being (SDG 3).

## Supporting information

S1 Text Population model structure. (DOCX)

S2 Text Data sources and calculations for population model. (DOCX)

S1 Table Overview of variables and their data sources for population model. (DOCX)

S1 Fig Causal loop diagram representing a high-level structure of main feedback mechanisms comprising the system dynamics model used in this study. (DOCX)

S2 Table Overview of revenue reallocation scenarios. (DOCX)

S3 Table Exogenous model parameter values and their data sources for the policy scenario modelling. (DOCX)

S3 Text Behaviour reproduction. (DOCX)

S4 Text Model equations. (DOCX)

S5 Text Sensitivity testing. (DOCX)

S6 Text Extreme condition testing. (DOCX)

S7 Text Comparison of integration methods and time steps. (DOCX)

S4 Table Variables and their parameters used in the probabilistic sensitivity analysis. (DOCX)

S2 Fig Policy mechanisms showing changes in (A) Juntos Basic Transfer coverage in children, (B) Percentage change in Juntos Basic Transfer cash amount, (C) Juntos Early Childhood Transfer coverage in children under-5, and (D) Fruit and vegetable subsidy rate, under different scenarios. (DOCX)

S5 Table Cumulative person-years lived with overweight in adults of reproductive age by 2040 under sensitivity analysis assuming Juntos effect on overweight in adults, equal to the Juntos effect on overweight in children, for Scenarios A and B, overall and by socioeconomic group. (DOCX)

S1 Alternative Language Abstract File contains Spanish language abstract. (DOCX)

## Abbreviations

CHRC: Comprehensive Health Research Center
DBM: double burden of malnutrition
LMICs: low- and middle-income countries
SDGs: Sustainable Development Goals
SSBs: sugar-sweetened beverages
UI: Uncertainty Interval
UPFs: ultra-processed foods
WHO: World Health Organization.
