# Supplementary material for: Retrofitting fiscal policies to reduce the double burden of malnutrition in Peru: A system dynamics modelling study
Source: PLoS Med. 2026 Aug 13;23(8):e1005167. doi: 10.1371/journal.pmed.1005167 (PMC13472416; doi:10.1371/journal.pmed.1005167)
Supplement: Supplementary file 1 [file pmed.1005167.s001.docx]

**S1 Text: Population Model Structure**

We developed a System Dynamics model to simulate trends in overweight and stunting among children and adults of reproductive age in Peru, from 2000 to 2040. The model follows Sterman’s standard stock-and-flow formulations for population dynamics, and builds upon our prior modelling work.[1] It comprises three interconnected aging chains (co-flows) representing: (1) the overall population, (2) the stunted/short-stature population, and (3) the overweight population. The overall population aging chain simulates demographic trends, which in turn inform the dynamics of the stunting and overweight co-flows (Figure 1).

Each of the three aging chains is organised into child and adult stocks, further stratified into age groups (0-4, 5-9, 10-14 years for children, and 15-24, 25-34, 35-49 years for adults) and two socioeconomic groups. The separation into child and adult stocks reflects the onset of reproduction at age 15, enabling the simulation of births and intergenerational transmission cycles through feedback loops. Adults over the age of 50 were excluded due to the limited availability of data for this age group in Peru. Socioeconomic stratification was divided into two groups: the poorest 20% (lowest quintile) and the remaining 80% (upper quintiles). This stratification reflects the distribution of overweight and stunting across wealth index quintiles from nationally representative Demographic and Health Survey (DHS) data, where stunting is most prevalent in the lowest quintile, while overweight prevalence is lowest in this group (Figure 2).

Changes in the stocks of stunted or overweight individuals are influenced by transitions between nutritional states (Figure 1). Individuals can recover from stunting or overweight and move back to a normal nutritional state (recovery outflows), or they may transition from the normal state to stunted or overweight stocks (faltering inflows). These flows are informed by transition probabilities for each age and socioeconomic group (see Supplementary Text S2). Furthermore, the model captures the intergenerational transmission of stunting through maternal and neonatal characteristics, by incorporating the additional burden of low birth weight attributed to adult short stature, as well as the increased risk of stunting associated with low birth weight (Figure 1). These relationships were modelled using relative risks derived from meta-analyses of longitudinal studies.^[2-5]^

 **Figure 1.** Representation of the overall population aging chain (left) and the stunting co-flow (right). The reinforcing feedback loop (**R**), highlighted in red, illustrates the relationship between adult short stature and stunting, through LBW. The overweight co-flow follows the same structural framework as the stunting co-flow but does not include the intergenerational transmission loop. LBW = low birth weight; RR: Relative Risk.

**Figure 2.** (A) Trends in overweight prevalence and (B) trends in stunting prevalence from 2000 to 2023. Prevalences are disaggregated by wealth quintiles, based on Demographic and Health Survey (DHS) data.

**References**

1.	Seferidi P, Guzman-Abello L, Ballard E, Creed-Kanashiro HM, Huicho L, Miranda JJ, et al. Actions against the double burden of malnutrition in Peru: a community-informed system dynamics model. Lancet Reg Health Am. 2025;46:101102. Epub 20250421. doi: 10.1016/j.lana.2025.101102. PubMed PMID: 40329938; PubMed Central PMCID: PMCPMC12052979.

2.	Schellong K, Schulz S, Harder T, Plagemann A. Birth weight and long-term overweight risk: systematic review and a meta-analysis including 643,902 persons from 66 studies and 26 countries globally. PLoS One. 2012;7(10):e47776. Epub 20121017. doi: 10.1371/journal.pone.0047776. PubMed PMID: 23082214; PubMed Central PMCID: PMCPMC3474767.

3.	Christian P, Lee SE, Donahue Angel M, Adair LS, Arifeen SE, Ashorn P, et al. Risk of childhood undernutrition related to small-for-gestational age and preterm birth in low- and middle-income countries. Int J Epidemiol. 2013;42(5):1340-55. Epub 20130806. doi: 10.1093/ije/dyt109. PubMed PMID: 23920141; PubMed Central PMCID: PMCPMC3816349.

4.	Kozuki N, Katz J, Lee AC, Vogel JP, Silveira MF, Sania A, et al. Short Maternal Stature Increases Risk of Small-for-Gestational-Age and Preterm Births in Low- and Middle-Income Countries: Individual Participant Data Meta-Analysis and Population Attributable Fraction. J Nutr. 2015;145(11):2542-50. Epub 20150930. doi: 10.3945/jn.115.216374. PubMed PMID: 26423738; PubMed Central PMCID: PMCPMC6457093.

5.	Vats H, Saxena R, Sachdeva MP, Walia GK, Gupta V. Impact of maternal pre-pregnancy body mass index on maternal, fetal and neonatal adverse outcomes in the worldwide populations: A systematic review and meta-analysis. Obes Res Clin Pract. 2021;15(6):536-45. Epub 20211112. doi: 10.1016/j.orcp.2021.10.005. PubMed PMID: 34782256.
