# Supplementary material for: Retrofitting fiscal policies to reduce the double burden of malnutrition in Peru: A system dynamics modelling study
Source: PLoS Med. 2026 Aug 13;23(8):e1005167. doi: 10.1371/journal.pmed.1005167 (PMC13472416; doi:10.1371/journal.pmed.1005167)
Supplement: Supplementary file 2 [file pmed.1005167.s002.docx]

**S2 Text. Data sources and calculations for population model**

The model was informed by demographic and anthropometric data from Peru, including age-specific fertility rates, gender proportions, all-cause mortality rates, and stunting and overweight prevalence (Table 1). We used data from 2000 to inform baseline values and data from 2000 and 2020 to estimate annual change rates up to 2020 to model behaviour over time. Socioeconomic differences were incorporated into the model by applying age-specific baseline prevalence rates for stunting and overweight, and stratified transition probabilities.

We estimated baseline age-specific transition probabilities between nutritional states for children aged 1 to 15 years, using data from the Young Lives cohort (Younger Cohort, samples from 2002–2016) and a previously established Markov Chain model.[1] We used the model’s odds ratios (ORs) for socioeconomic status, which represent the relative likelihood of transitioning into a specific state for the two socioeconomic groups, to calculate stratified baseline transition probabilities for lower and upper socioeconomic groups.

We estimated baseline transition probabilities in adults, using repeated cross-sectional data from the DHS and a heuristic developed by Meisel et al., given that longitudinal data were unavailable.[1, 2] We estimated transition probabilities for each 5-year age group, using prevalence data from 2000 and 2005, assuming that individuals in each age group in 2000 correspond to the same individuals aged five years older in 2005, leveraging the national representativeness of the DHS data. Since the adult stocks in the model include 10- and 15-year age groups, we used the mean transition probabilities of adjacent 5-year age groups to align with the model’s structure. We assumed no transitions in and out of stunting/short stature for individuals over the age of 24.

We modelled behaviour over time by adjusting baseline transition probabilities using age group-specific annual change rates, stratified by socioeconomic groups. We estimated annual change rates using data from the DHS for children under 5 and adults. For 2000 to 2020, we estimated the annual change in prevalence by taking the 5-year difference in prevalence and dividing it by 5. For the time from 2020 to 2023, same approach was used, but for a 3 year period. Since DHS data for ages 5-15 were unavailable, we approximated observed trends for stunting and overweight for these age groups by applying the same change rates we applied for ages 0-5 to baseline transition probabilities for ages 5-15. We assumed that change rates capture the period effect of trends, which is the same across all age groups, whereas the baseline transition probabilities capture the age-effect, which is age-specific. This approach accurately reflected observed stunting and overweight trends during model testing (see Supplementary Text S3). For short stature adults, annual changes were applied for the age group 15-24, assuming stagnant growth thereafter, while for overweight, consistent annual change rates were applied throughout adulthood.

**References**

1.	Escher NA, Carrillo-Larco RM, Parnham JC, Curi-Quinto K, Ghosh-Jerath S, Millett C, et al. Longitudinal transitions of the double burden of overweight and stunting from childhood to early adulthood in India, Peru, and Vietnam. Int J Epidemiol. 2024;53(6). doi: 10.1093/ije/dyae151. PubMed PMID: 39545485; PubMed Central PMCID: PMCPMC11565240.

2.	Meisel JD, Sarmiento OL, Olaya C, Lemoine PD, Valdivia JA, Zarama R. Towards a novel model for studying the nutritional stage dynamics of the Colombian population by age and socioeconomic status. PLoS One. 2018;13(2):e0191929. Epub 20180208. doi: 10.1371/journal.pone.0191929. PubMed PMID: 29420563; PubMed Central PMCID: PMCPMC5805245.
