# Supplementary material for: Retrofitting fiscal policies to reduce the double burden of malnutrition in Peru: A system dynamics modelling study
Source: PLoS Med. 2026 Aug 13;23(8):e1005167. doi: 10.1371/journal.pmed.1005167 (PMC13472416; doi:10.1371/journal.pmed.1005167)
Supplement: Supplementary file 3 [file pmed.1005167.s003.docx]

**S1 Table. Overview of variables and their data sources for population model**

| Variable (unit) [subscript] | value | Source | Note |
| --- | --- | --- | --- |
| adult cohort duration (Year) [15-24] | 10 | NA | duration of each age group: 15-24, 25-34, 35-49 |
| adult cohort duration (Year) [25-34] | 10 | NA | duration of each age group: 15-24, 25-34, 35-49 |
| adult cohort duration (Year) [35-49] | 15 | NA | duration of each age group: 15-24, 25-34, 35-49 |
| adult mortality rate annual change (Dmnl/Year/Year) [15-24] | -2.1E-05 | INEI. Perú: Situación y Perspectivas de la Mortalidad por Sexo y Grupos de Edad, Nacional y por Departamentos, 1990 – 2025. <https://proyectos.inei.gob.pe/web/biblioineipub/bancopub/est/lib0901/libro.pdf>. (accessed January 15 2025). [1] | Estimated as annual mortality rate change between 2000 and 2020 (mean of 5-year age groups) |
| adult mortality rate annual change (Dmnl/Year/Year) [25-34] | -3.2E-05 | INEI. Perú: Situación y Perspectivas de la Mortalidad por Sexo y Grupos de Edad, Nacional y por Departamentos, 1990 – 2025. <https://proyectos.inei.gob.pe/web/biblioineipub/bancopub/est/lib0901/libro.pdf>. (accessed January 15 2025). [1] | Estimated as annual mortality rate change between 2000 and 2020 (mean of 5-year age groups) |
| adult mortality rate annual change (Dmnl/Year/Year) [35-49] | -0.00006 | INEI. Perú: Situación y Perspectivas de la Mortalidad por Sexo y Grupos de Edad, Nacional y por Departamentos, 1990 – 2025. <https://proyectos.inei.gob.pe/web/biblioineipub/bancopub/est/lib0901/libro.pdf>. (accessed January 15 2025). [1] | Estimated as annual mortality rate change between 2000 and 2020 (mean of 5-year age groups) |
| Baseline adult mortality rate (Dmnl/Year ) [15-24] | 0.00137 | INEI. Perú: Situación y Perspectivas de la Mortalidad por Sexo y Grupos de Edad, Nacional y por Departamentos, 1990 – 2025. <https://proyectos.inei.gob.pe/web/biblioineipub/bancopub/est/lib0901/libro.pdf>. (accessed January 15 2025). [1] | Mean estimated mortality rates of 5-year age groups in 2000 |
| Baseline adult mortality rate (Dmnl/Year ) [25-34] | 0.002157 | INEI. Perú: Situación y Perspectivas de la Mortalidad por Sexo y Grupos de Edad, Nacional y por Departamentos, 1990 – 2025. <https://proyectos.inei.gob.pe/web/biblioineipub/bancopub/est/lib0901/libro.pdf>. (accessed January 15 2025). [1] | Mean estimated mortality rates of 5-year age groups in 2000 |
| Baseline adult mortality rate (Dmnl/Year ) [35-49] | 0.00454 | INEI. Perú: Situación y Perspectivas de la Mortalidad por Sexo y Grupos de Edad, Nacional y por Departamentos, 1990 – 2025. <https://proyectos.inei.gob.pe/web/biblioineipub/bancopub/est/lib0901/libro.pdf>. (accessed January 15 2025). [1] | Mean estimated mortality rates of 5-year age groups in 2000 |
| Baseline adult overweight prevalence (Dmnl) [15-24, lower] | 0.198715 | DHS Perú 2000 | Prevalence of overweight (BMI≥25) in women 15-49 in Peru, 2000, for poorest 20% and wealthiest 80% of population. No data on men. |
| Baseline adult overweight prevalence (Dmnl) [15-24, upper] | 0.261599 | DHS Perú 2000 | Prevalence of overweight (BMI≥25) in women 15-49 in Peru, 2000, for poorest 20% and wealthiest 80% of population. No data on men. |
| Baseline adult overweight prevalence (Dmnl) [25-34, lower] | 0.321429 | DHS Perú 2000 | Prevalence of overweight (BMI≥25) in women 15-49 in Peru, 2000, for poorest 20% and wealthiest 80% of population. No data on men. |
| Baseline adult overweight prevalence (Dmnl) [25-34, upper] | 0.501211 | DHS Perú 2000 | Prevalence of overweight (BMI≥25) in women 15-49 in Peru, 2000, for poorest 20% and wealthiest 80% of population. No data on men. |
| Baseline adult overweight prevalence (Dmnl) [35-49, lower] | 0.371169 | DHS Perú 2000 | Prevalence of overweight (BMI≥25) in women 15-49 in Peru, 2000, for poorest 20% and wealthiest 80% of population. No data on men. |
| Baseline adult overweight prevalence (Dmnl) [35-49, upper] | 0.660353 | DHS Perú 2000 | Prevalence of overweight (BMI≥25) in women 15-49 in Peru, 2000, for poorest 20% and wealthiest 80% of population. No data on men. |
| baseline adult TP overweight faltering (Dmnl)[A15to24] | 0.16047 | DHS Perú 2000 and 2005 | Transition probabilities (TP) are calculated using the heuristic method from Meisel et al. for five-year age groups. These probabilities are then averaged and adjusted to match the cohort duration. |
| baseline adult TP overweight faltering (Dmnl)[A35to49] | 0.107676 | DHS Perú 2000 and 2005 | Transition probabilities (TP) are calculated using the heuristic method from Meisel et al. for five-year age groups. These probabilities are then averaged and adjusted to match the cohort duration. |
| baseline adult TP overweight faltering(Dmnl)[A25to34] | 0.172701 | DHS Perú 2000 and 2005 | Transition probabilities (TP) are calculated using the heuristic method from Meisel et al. for five-year age groups. These probabilities are then averaged and adjusted to match the cohort duration. |
| baseline adult TP overweight recovery (Dmnl) [15-24] | 5.23E-21 | DHS Perú 2000 and 2005 | Transition probabilities (TP) are calculated using the heuristic method from Meisel et al. for five-year age groups. These probabilities are then averaged and adjusted to match the cohort duration. |
| baseline adult TP overweight recovery (Dmnl) [25-34] | 1.32E-23 | DHS Perú 2000 and 2005 | Transition probabilities (TP) are calculated using the heuristic method from Meisel et al. for five-year age groups. These probabilities are then averaged and adjusted to match the cohort duration. |
| baseline adult TP overweight recovery (Dmnl) [35-49] | 0.025901 | DHS Perú 2000 and 2005 | Transition probabilities (TP) are calculated using the heuristic method from Meisel et al. for five-year age groups. These probabilities are then averaged and adjusted to match the cohort duration. |
| baseline adult TP stunting faltering (Dmnl)[15-24] | 0.000538 | DHS Perú 2000 and 2005 | Transition probabilities (TP) are calculated using the heuristic method from Meisel et al. for five-year age groups. These probabilities are then averaged and adjusted to match the cohort duration. |
| baseline adult TP stunting faltering (Dmnl)[25-34] | 0 |  | No transitions of stunting are assumed after age 24 |
| baseline adult TP stunting faltering (Dmnl)[35-49] | 0 |  | No transitions of stunting are assumed after age 24 |
| baseline adult TP stunting recovery (Dmnl) [15-24] | 0.243851 | DHS Perú 2000 and 2005 | Transition probabilities (TP) are calculated using the heuristic method from Meisel et al. for five-year age groups. These probabilities are then averaged and adjusted to match the cohort duration. |
| baseline adult TP stunting recovery (Dmnl) [25-34] | 0 |  | No transitions of stunting are assumed after age 24 |
| baseline adult TP stunting recovery (Dmnl) [35-49] | 0 |  | No transitions of stunting are assumed after age 24 |
| baseline child mortality rate (Dmnl/Year) [0-4] | 0.015913 | INEI. Perú: Situación y Perspectivas de la Mortalidad por Sexo y Grupos de Edad, Nacional y por Departamentos, 1990 – 2025. <https://proyectos.inei.gob.pe/web/biblioineipub/bancopub/est/lib0901/libro.pdf>. (accessed January 15 2025). [1] | Mean estimated mortality rates of 5-year age groups in 2000 |
| baseline child mortality rate (Dmnl/Year) [10-14] | 0.0008 | INEI. Perú: Situación y Perspectivas de la Mortalidad por Sexo y Grupos de Edad, Nacional y por Departamentos, 1990 – 2025. <https://proyectos.inei.gob.pe/web/biblioineipub/bancopub/est/lib0901/libro.pdf>. (accessed January 15 2025). [1] | Mean estimated mortality rates of 5-year age groups in 2000 |
| baseline child mortality rate (Dmnl/Year)[5-9] | 0.000855 | INEI. Perú: Situación y Perspectivas de la Mortalidad por Sexo y Grupos de Edad, Nacional y por Departamentos, 1990 – 2025. <https://proyectos.inei.gob.pe/web/biblioineipub/bancopub/est/lib0901/libro.pdf>. (accessed January 15 2025). [1] | Mean estimated mortality rates of 5-year age groups in 2000 |
| baseline child overweight prevalence (Dmnl)[0-4, lower] | 0.052657 | DHS Perú 2000 | Overweight prevalence (weight for height z-score>2SD) in children under-5 in Peru, 2000, for poorest 20% of population |
| baseline child overweight prevalence (Dmnl)[0-4, upper] | 0.071896 | DHS Perú 2000 | Overweight prevalence (weight for height z-score>2SD) in children under-5 in Peru, 2000, for wealthiest 80% of population |
| baseline child overweight prevalence (Dmnl)[10-14, lower] | 0.202825 | Young Lives Cohort Peru (Older Cohort) | Overweight prevalence (weight-for-height z-score > 2SD) in the Older Cohort during Round 1 (2002), when they were 8 years old, based on data from the Young Lives Cohort, for poorest 20% of population |
| baseline child overweight prevalence (Dmnl)[10-14, upper] | 0.213458 | Young Lives Cohort Peru (Older Cohort) | Overweight prevalence (weight-for-height z-score > 2SD) in the Older Cohort during Round 1 (2002), when they were 8 years old, based on data from the Young Lives Cohort, for wealthiest 80% of population |
| baseline child overweight prevalence (Dmnl)[5-9, lower] | 0.202825 | Young Lives Cohort Peru (Older Cohort) | Overweight prevalence (weight-for-height z-score > 2SD) in the Older Cohort during Round 1 (2002), when they were 8 years old, based on data from the Young Lives Cohort, for poorest 20% of population |
| baseline child overweight prevalence (Dmnl)[5-9, upper] | 0.213458 | Young Lives Cohort Peru (Older Cohort) | Overweight prevalence (weight-for-height z-score > 2SD) in the Older Cohort during Round 1 (2002), when they were 8 years old, based on data from the Young Lives Cohort, for wealthiest 80% of population |
| baseline child stunting prevalence (Dmnl)[0-4, lower] | 0.459776 | DHS Perú 2000 | Stunting prevalence (height for age z-score<-2SD) for children under-5 in Peru in 2000, for poorest 20% of population |
| baseline child stunting prevalence (Dmnl)[0-4, upper] | 0.199653 | DHS Perú 2000 | Stunting prevalence (height for age z-score<-2SD) for children under-5 in Peru in 2000, for wealthiest 80% of population |
| baseline child stunting prevalence (Dmnl)[10-14, lower] | 0.4091 | Young Lives Cohort Peru (Older Cohort) | Stunting prevalence (height-for-age z-score > 2SD) in the Older Cohort during Round 1 (2002), when they were 8 years old, based on data from the Young Lives Cohort, for poorest 20% of population |
| baseline child stunting prevalence (Dmnl)[10-14, upper] | 0.1682 | Young Lives Cohort Peru (Older Cohort) | Stunting prevalence (height-for-age z-score > 2SD) in the Older Cohort during Round 1 (2002), when they were 8 years old, based on data from the Young Lives Cohort, for wealthiest 80% of population |
| baseline child stunting prevalence (Dmnl)[5-9, lower] | 0.4091 | Young Lives Cohort Peru (Older Cohort) | Stunting prevalence (height-for-age z-score > 2SD) in the Older Cohort during Round 1 (2002), when they were 8 years old, based on data from the Young Lives Cohort, for poorest 20% of population |
| baseline child stunting prevalence (Dmnl)[5-9, upper] | 0.1682 | Young Lives Cohort Peru (Older Cohort) | Stunting prevalence (height-for-age z-score > 2SD) in the Older Cohort during Round 1 (2002), when they were 8 years old, based on data from the Young Lives Cohort, for wealthiest 80% of population |
| baseline child TP overweight faltering (Dmnl)[0-4] | 0.177084 | Young Lives Cohort Peru (Younger Cohort) | The transition probability (TP) was calculated using a Markov Chain model for children aged 1–5 (Younger Cohort, round 1 to 2), estimated retrospectively for 2000 using Annual Change Rates, and adjusted for the 5-year cohort duration. |
| baseline child TP overweight faltering (Dmnl)[10-14] | 0.07382 | Young Lives Cohort Peru (Younger Cohort) | The transition probability (TP) was calculated using a Markov Chain model for children aged 8–15 (Younger Cohort, round 3 to 5), estimated retrospectively for 2000 using Annual Change Rates, and adjusted for the 5-year cohort duration. |
| baseline child TP overweight faltering (Dmnl)[5-9] | 0.253153 | Young Lives Cohort Peru (Younger Cohort) | The transition probability (TP) was calculated using a Markov Chain model for children aged 5–8 (Younger Cohort, round 2 to 3), estimated retrospectively for 2000 using Annual Change Rates, and adjusted for the 5-year cohort duration. |
| baseline child TP overweight recovery (Dmnl)[0-4] | 0.693378 | Young Lives Cohort Peru (Younger Cohort) | The transition probability (TP) was calculated using a Markov Chain model for children aged 1–5 (Younger Cohort, round 1 to 2), estimated retrospectively for 2000 using Annual Change Rates, and adjusted for the 5-year cohort duration. |
| baseline child TP overweight recovery (Dmnl)[10-14] | 0.379123 | Young Lives Cohort Peru (Younger Cohort) | The transition probability (TP) was calculated using a Markov Chain model for children aged 8–15 (Younger Cohort, round 3 to 5), estimated retrospectively for 2000 using Annual Change Rates, and adjusted for the 5-year cohort duration. |
| baseline child TP overweight recovery (Dmnl)[5-9] | 0.688826 | Young Lives Cohort Peru (Younger Cohort) | The transition probability (TP) was calculated using a Markov Chain model for children aged 5–8 (Younger Cohort, round 2 to 3), estimated retrospectively for 2000 using Annual Change Rates, and adjusted for the 5-year cohort duration. |
| baseline child TP stunting faltering (Dmnl)[0-4] | 0.280259 | Young Lives Cohort Peru (Younger Cohort) | The transition probability (TP) was calculated using a Markov Chain model for children aged 1–5 (Younger Cohort, round 1 to 2), estimated retrospectively for 2000 using Annual Change Rates, and adjusted for the 5-year cohort duration. |
| baseline child TP stunting faltering (Dmnl)[10-14] | 0.090927 | Young Lives Cohort Peru (Younger Cohort) | The transition probability (TP) was calculated using a Markov Chain model for children aged 8–15 (Younger Cohort, round 3 to 5), estimated retrospectively for 2000 using Annual Change Rates, and adjusted for the 5-year cohort duration. |
| baseline child TP stunting faltering (Dmnl)[5-9] | 0.087622 | Young Lives Cohort Peru (Younger Cohort) | The transition probability (TP) was calculated using a Markov Chain model for children aged 5–8 (Younger Cohort, round 2 to 3), estimated retrospectively for 2000 using Annual Change Rates, and adjusted for the 5-year cohort duration. |
| baseline child TP stunting recovery (Dmnl)[0-4] | 0.37824 | Young Lives Cohort Peru (Younger Cohort) | The transition probability (TP) was calculated using a Markov Chain model for children aged 1–5 (Younger Cohort, round 1 to 2), estimated retrospectively for 2000 using Annual Change Rates, and adjusted for the 5-year cohort duration. |
| baseline child TP stunting recovery (Dmnl)[10-14] | 0.277992 | Young Lives Cohort Peru (Younger Cohort) | The transition probability (TP) was calculated using a Markov Chain model for children aged 8–15 (Younger Cohort, round 3 to 5), estimated retrospectively for 2000 using Annual Change Rates, and adjusted for the 5-year cohort duration. |
| baseline child TP stunting recovery (Dmnl)[5-9] | 0.763643 | Young Lives Cohort Peru (Younger Cohort) | The transition probability (TP) was calculated using a Markov Chain model for children aged 5–8 (Younger Cohort, round 2 to 3), estimated retrospectively for 2000 using Annual Change Rates, and adjusted for the 5-year cohort duration. |
| baseline fertility rate (Dmnl/Year )[15-24] | 0.103 | INEI. Perú: Situación y Perspectivas de la Mortalidad por Sexo y Grupos de Edad, Nacional y por Departamentos, 1990 – 2025. <https://proyectos.inei.gob.pe/web/biblioineipub/bancopub/est/lib0901/libro.pdf>. (accessed January 15 2025). [1] | Fertility rate (births per woman) by age group (mean of 5-year age groups) |
| baseline fertility rate (Dmnl/Year )[25-34] | 0.123 | INEI. Perú: Situación y Perspectivas de la Mortalidad por Sexo y Grupos de Edad, Nacional y por Departamentos, 1990 – 2025. <https://proyectos.inei.gob.pe/web/biblioineipub/bancopub/est/lib0901/libro.pdf>. (accessed January 15 2025). [1] | Fertility rate (births per woman) by age group (mean of 5-year age groups) |
| baseline fertility rate (Dmnl/Year )[35-49] | 0.039 | INEI. Perú: Situación y Perspectivas de la Mortalidad por Sexo y Grupos de Edad, Nacional y por Departamentos, 1990 – 2025. <https://proyectos.inei.gob.pe/web/biblioineipub/bancopub/est/lib0901/libro.pdf>. (accessed January 15 2025). [1] | Fertility rate (births per woman) by age group (mean of 5-year age groups) |
| baseline infant overweight prevalence (Dmnl) [lower] | 0.0993 | DHS Perú 2000 | Prevalence of overweight (weight for height z-score>2SD) in children under-1 in Peru, 2000, for poorest 20% of population. Estimated from Peru DHS, 2000 |
| baseline infant overweight prevalence (Dmnl) [upper] | 0.116 | DHS Perú 2000 | Prevalence of overweight (weight for height z-score>2SD) in children under-1 in Peru, 2000, for wealthiest 80% of population. Estimated from Peru DHS, 2000 |
| baseline infant stunting (Dmnl) [lower] | 0.135 | DHS Perú 2000 | Stunting prevalence (height for age z-score<-2SD) for children under-1 in Peru in 2000, for poorest 20% of population. Estimated from Peru DHS, 2000 |
| baseline infant stunting (Dmnl) [upper] | 0.063 | DHS Perú 2000 | Stunting prevalence (height for age z-score<-2SD) for children under-1 in Peru in 2000, for wealthiest 80% of population. Estimated from Peru DHS, 2000 |
| baseline LBW (Dmnl) | 0.102 | WHO. Global Health Observatory data repository. Low Birth Weight. Data by country. 2023. https://apps.who.int/gho/data/view.main.LBWCOUNTRYv?lang=en (accessed January 15 2025). [2] | Low birth weight (LBW) value for year 2000 in Peru |
| baseline population adults (people)[15-24] | 5264450 | INEI. Perú: Estimaciones y Proyecciones de la Población Nacional, Por Año Calendario y Edad Simple, 1950-2050, Boletín Especial Nº 24. Lima, 2019. <https://www.inei.gob.pe/media/MenuRecursivo/publicaciones_digitales/Est/Lib1681/libro.pdf> (accessed January 15 2025). [3] | Total population by age group in 2000. |
| baseline population adults (people)[25-34] | 4202240 | INEI. Perú: Estimaciones y Proyecciones de la Población Nacional, Por Año Calendario y Edad Simple, 1950-2050, Boletín Especial Nº 24. Lima, 2019. <https://www.inei.gob.pe/media/MenuRecursivo/publicaciones_digitales/Est/Lib1681/libro.pdf> (accessed January 15 2025). [3] | Total population by age group in 2000. |
| baseline population adults (people)[35-49] | 4278320 | INEI. Perú: Estimaciones y Proyecciones de la Población Nacional, Por Año Calendario y Edad Simple, 1950-2050, Boletín Especial Nº 24. Lima, 2019. <https://www.inei.gob.pe/media/MenuRecursivo/publicaciones_digitales/Est/Lib1681/libro.pdf> (accessed January 15 2025). [3] | Total population by age group in 2000. |
| baseline population children (people) [0-4] | 2994960 | INEI. Perú: Estimaciones y Proyecciones de la Población Nacional, Por Año Calendario y Edad Simple, 1950-2050, Boletín Especial Nº 24. Lima, 2019. <https://www.inei.gob.pe/media/MenuRecursivo/publicaciones_digitales/Est/Lib1681/libro.pdf> (accessed January 15 2025). [3] | Total population by age group in 2000. |
| baseline population children (people)[10-14] | 3021560 | INEI. Perú: Estimaciones y Proyecciones de la Población Nacional, Por Año Calendario y Edad Simple, 1950-2050, Boletín Especial Nº 24. Lima, 2019. <https://www.inei.gob.pe/media/MenuRecursivo/publicaciones_digitales/Est/Lib1681/libro.pdf> (accessed January 15 2025). [3] | Total population by age group in 2000. |
| baseline population children (people)[5-9] | 3083200 | INEI. Perú: Estimaciones y Proyecciones de la Población Nacional, Por Año Calendario y Edad Simple, 1950-2050, Boletín Especial Nº 24. Lima, 2019. <https://www.inei.gob.pe/media/MenuRecursivo/publicaciones_digitales/Est/Lib1681/libro.pdf> (accessed January 15 2025). [3] | Total population by age group in 2000. |
| baseline short stature prevalence (Dmnl)[0-4, upper] | 0.087735 | DHS Perú 2000 | Prevalence of short stature (height<145cm) in women in Peru, 2000, for wealthiest 80% of population. No data on men. |
| baseline short stature prevalence (Dmnl)[0-4,lower] | 0.195501 | DHS Perú 2000 | Prevalence of short stature (height<145cm) in women in Peru, 2000, for poorest 20% of population. No data on men |
| baseline short stature prevalence (Dmnl)[10-14, upper] | 0.13295 | DHS Perú 2000 | Prevalence of short stature (height<145cm) in women in Peru, 2000, for wealthiest 80% of population. No data on men. |
| baseline short stature prevalence (Dmnl)[10-14,lower] | 0.203542 | DHS Perú 2000 | Prevalence of short stature (height<145cm) in women in Peru, 2000, for poorest 20% of population. No data on men |
| baseline short stature prevalence (Dmnl)[5-9, upper] | 0.09377 | DHS Perú 2000 | Prevalence of short stature (height<145cm) in women in Peru, 2000, for wealthiest 80% of population. No data on men. |
| baseline short stature prevalence (Dmnl)[5-9,lower] | 0.158257 | DHS Perú 2000 | Prevalence of short stature (height<145cm) in women in Peru, 2000, for poorest 20% of population. No data on men |
| child cohort duration (Year)[0-4] | 5 | NA | duration of each age group: 0-4, 5-9, 10-14 |
| child cohort duration (Year)[10-14] | 5 | NA | duration of each age group: 0-4, 5-9, 10-14 |
| child cohort duration (Year)[5-9] | 5 | NA | duration of each age group: 0-4, 5-9, 10-14 |
| child mortality rate annual change (Dmnl/Year/Year)[0-4] | -0.00047 | INEI. Perú: Situación y Perspectivas de la Mortalidad por Sexo y Grupos de Edad, Nacional y por Departamentos, 1990 – 2025. <https://proyectos.inei.gob.pe/web/biblioineipub/bancopub/est/lib0901/libro.pdf>. (accessed January 15 2025). [1] | Estimated as annual mortality rate change between 2000 and 2020 (mean of 5-year age groups) |
| child mortality rate annual change (Dmnl/Year/Year)[10-14] | -1.3E-05 | INEI. Perú: Situación y Perspectivas de la Mortalidad por Sexo y Grupos de Edad, Nacional y por Departamentos, 1990 – 2025. <https://proyectos.inei.gob.pe/web/biblioineipub/bancopub/est/lib0901/libro.pdf>. (accessed January 15 2025). [1] | Estimated as annual mortality rate change between 2000 and 2020 (mean of 5-year age groups) |
| child mortality rate annual change (Dmnl/Year/Year)[5-9] | -1.6E-05 | INEI. Perú: Situación y Perspectivas de la Mortalidad por Sexo y Grupos de Edad, Nacional y por Departamentos, 1990 – 2025. <https://proyectos.inei.gob.pe/web/biblioineipub/bancopub/est/lib0901/libro.pdf>. (accessed January 15 2025). [1] | Estimated as annual mortality rate change between 2000 and 2020 (mean of 5-year age groups) |
| female percentage (Dmnl) | 0.501 | World Bank, Perú | Population, female (% of total population) in 2000 |
| fertility rate annual change (Dmnl/Year/Year) [15-24] | -0.002175 | INEI Perú, Informe Principal Encuesta Demográfica y de Salud Familiar | Fertility rate changes between 2000 and 2020, according to observed data. After 2020, it does not change. |
| fertility rate annual change (Dmnl/Year/Year) [25-34] | -0.0016 | INEI Perú, Informe Principal Encuesta Demográfica y de Salud Familiar | Fertility rate changes between 2000 and 2020, according to observed data. After 2020, it does not change. |
| fertility rate annual change (Dmnl/Year/Year) [35-49] | -0.00055 | INEI Perú, Informe Principal Encuesta Demográfica y de Salud Familiar | Fertility rate changes between 2000 and 2020, according to observed data. After 2020, it does not change. |
| FINAL TIME (Year) | 2040 | NA |  |
| INITIAL TIME (Year) | 2000 | NA |  |
| LBW and stunting RR (Dmnl) | 2.23 | Christian et al. Risk of childhood undernutrition related to small-for-gestational age and preterm birth in low- and middle-income countries. Int J Epidemiol. 2013;42(5):1340-1355. doi:10.1093/ije/dyt109. [4] | Regional OR for stunting at 12 to 60 months among babies born low birth weight in Latin America from a meta-analysis of birth cohorts. |
| maternal height and LBW RR (Dmnl) | 1.98 | Kozuki et al. Short Maternal Stature Increases Risk of Small-for-Gestational-Age and Preterm Births in Low- and Middle-Income Countries: Individual Participant Data Meta-Analysis and Population Attributable Fraction. J Nutr. 2015;145(11):2542-2550. doi:10.3945/jn.115.216374. [5] | Pooled adjusted RRs between short maternal stature (<145 cm, reference: ≥155 cm) and short for gestational age (<10% compared to ≥10%) |
| OR wealth overweight faltering adults (Dmnl) | 0.549111 | Young Lives Cohort Peru (Younger Cohort) | The Odds Ratio (OR), calculated from the Markov Chain model, represents the relative odds of wealthier individuals undergoing the specified transition compared to those in the poorest wealth group. |
| OR wealth overweight faltering children (Dmnl) | 1.038 | Young Lives Cohort Peru (Younger Cohort) | The Odds Ratio (OR), calculated from the Markov Chain model, represents the relative odds of wealthier individuals undergoing the specified transition compared to those in the poorest wealth group. |
| OR wealth overweight recovery adults (Dmnl) | 2.17521 | Young Lives Cohort Peru (Younger Cohort) | The Odds Ratio (OR), calculated from the Markov Chain model, represents the relative odds of wealthier individuals undergoing the specified transition compared to those in the poorest wealth group. |
| OR wealth overweight recovery children (Dmnl) | 1.61029 | Young Lives Cohort Peru (Younger Cohort) | The Odds Ratio (OR), calculated from the Markov Chain model, represents the relative odds of wealthier individuals undergoing the specified transition compared to those in the poorest wealth group. |
| OR wealth stunting faltering adults (Dmnl) | 3.03201 | Young Lives Cohort Peru (Younger Cohort) | The Odds Ratio (OR), calculated from the Markov Chain model, represents the relative odds of wealthier individuals undergoing the specified transition compared to those in the poorest wealth group. |
| OR wealth stunting faltering children (Dmnl) | 4.09991 | Young Lives Cohort Peru (Younger Cohort) | The Odds Ratio (OR), calculated from the Markov Chain model, represents the relative odds of wealthier individuals undergoing the specified transition compared to those in the poorest wealth group. |
| OR wealth stunting recovery adults (Dmnl) | 0.723105 | Young Lives Cohort Peru (Younger Cohort) | The Odds Ratio (OR), calculated from the Markov Chain model, represents the relative odds of wealthier individuals undergoing the specified transition compared to those in the poorest wealth group. |
| OR wealth stunting recovery children (Dmnl) | 0.612202 | Young Lives Cohort Peru (Younger Cohort) | The Odds Ratio (OR), calculated from the Markov Chain model, represents the relative odds of wealthier individuals undergoing the specified transition compared to those in the poorest wealth group. |
| overweight ACR adults 2000 (Dmnl/Year/Year)[15-24,lower] | -0.00963 | DHS Perú 2000-2005 | Annual Change Rates (ACR) in prevalence were calculated for 5-year age groups and averaged across these groups to align with the cohort duration. |
| overweight ACR adults 2000 (Dmnl/Year/Year)[15-24,upper] | 0.003628 | DHS Perú 2000-2005 | Annual Change Rates (ACR) in prevalence were calculated for 5-year age groups and averaged across these groups to align with the cohort duration. |
| overweight ACR adults 2000 (Dmnl/Year/Year)[25-34,lower] | -0.00338 | DHS Perú 2000-2005 | Annual Change Rates (ACR) in prevalence were calculated for 5-year age groups and averaged across these groups to align with the cohort duration. |
| overweight ACR adults 2000 (Dmnl/Year/Year)[25-34,upper] | 0.002854 | DHS Perú 2000-2005 | Annual Change Rates (ACR) in prevalence were calculated for 5-year age groups and averaged across these groups to align with the cohort duration. |
| overweight ACR adults 2000 (Dmnl/Year/Year)[35-49,lower] | 0.002981 | DHS Perú 2000-2005 | Annual Change Rates (ACR) in prevalence were calculated for 5-year age groups and averaged across these groups to align with the cohort duration. |
| overweight ACR adults 2000 (Dmnl/Year/Year)[35-49,upper] | 0.002598 | DHS Perú 2000-2005 | Annual Change Rates (ACR) in prevalence were calculated for 5-year age groups and averaged across these groups to align with the cohort duration. |
| overweight ACR adults 2005 (Dmnl/Year/Year)[15-24,lower] | 0.015045 | DHS Perú 2005-2010 | Annual Change Rates (ACR) in prevalence were calculated for 5-year age groups and averaged across these groups to align with the cohort duration. |
| overweight ACR adults 2005 (Dmnl/Year/Year)[15-24,upper] | 0.005103 | DHS Perú 2005-2010 | Annual Change Rates (ACR) in prevalence were calculated for 5-year age groups and averaged across these groups to align with the cohort duration. |
| overweight ACR adults 2005 (Dmnl/Year/Year)[25-34,lower] | 0.027095 | DHS Perú 2005-2010 | Annual Change Rates (ACR) in prevalence were calculated for 5-year age groups and averaged across these groups to align with the cohort duration. |
| overweight ACR adults 2005 (Dmnl/Year/Year)[25-34,upper] | 0.011381 | DHS Perú 2005-2010 | Annual Change Rates (ACR) in prevalence were calculated for 5-year age groups and averaged across these groups to align with the cohort duration. |
| overweight ACR adults 2005 (Dmnl/Year/Year)[35-49,lower] | 0.022961 | DHS Perú 2005-2010 | Annual Change Rates (ACR) in prevalence were calculated for 5-year age groups and averaged across these groups to align with the cohort duration. |
| overweight ACR adults 2005 (Dmnl/Year/Year)[35-49,upper] | 0.012442 | DHS Perú 2005-2010 | Annual Change Rates (ACR) in prevalence were calculated for 5-year age groups and averaged across these groups to align with the cohort duration. |
| overweight ACR adults 2010 (Dmnl/Year/Year)[15-24,lower] | 0.016419 | DHS Perú 2010-2015 | Annual Change Rates (ACR) in prevalence were calculated for 5-year age groups and averaged across these groups to align with the cohort duration. |
| overweight ACR adults 2010 (Dmnl/Year/Year)[15-24,upper] | 0.02088 | DHS Perú 2010-2015 | Annual Change Rates (ACR) in prevalence were calculated for 5-year age groups and averaged across these groups to align with the cohort duration. |
| overweight ACR adults 2010 (Dmnl/Year/Year)[25-34,lower] | 0.025223 | DHS Perú 2010-2015 | Annual Change Rates (ACR) in prevalence were calculated for 5-year age groups and averaged across these groups to align with the cohort duration. |
| overweight ACR adults 2010 (Dmnl/Year/Year)[25-34,upper] | 0.017232 | DHS Perú 2010-2015 | Annual Change Rates (ACR) in prevalence were calculated for 5-year age groups and averaged across these groups to align with the cohort duration. |
| overweight ACR adults 2010 (Dmnl/Year/Year)[35-49,lower] | 0.025831 | DHS Perú 2010-2015 | Annual Change Rates (ACR) in prevalence were calculated for 5-year age groups and averaged across these groups to align with the cohort duration. |
| overweight ACR adults 2010 (Dmnl/Year/Year)[35-49,upper] | 0.012886 | DHS Perú 2010-2015 | Annual Change Rates (ACR) in prevalence were calculated for 5-year age groups and averaged across these groups to align with the cohort duration. |
| overweight ACR adults 2015 (Dmnl/Year/Year)[15-24,lower] | 0.007249 | DHS Perú 2015-2020 | Annual Change Rates (ACR) in prevalence were calculated for 5-year age groups and averaged across these groups to align with the cohort duration. |
| overweight ACR adults 2015 (Dmnl/Year/Year)[15-24,upper] | 0.011362 | DHS Perú 2015-2020 | Annual Change Rates (ACR) in prevalence were calculated for 5-year age groups and averaged across these groups to align with the cohort duration. |
| overweight ACR adults 2015 (Dmnl/Year/Year)[25-34,lower] | 0.026278 | DHS Perú 2015-2020 | Annual Change Rates (ACR) in prevalence were calculated for 5-year age groups and averaged across these groups to align with the cohort duration. |
| overweight ACR adults 2015 (Dmnl/Year/Year)[25-34,upper] | 0.008482 | DHS Perú 2015-2020 | Annual Change Rates (ACR) in prevalence were calculated for 5-year age groups and averaged across these groups to align with the cohort duration. |
| overweight ACR adults 2015 (Dmnl/Year/Year)[35-49,lower] | 0.025895 | DHS Perú 2015-2020 | Annual Change Rates (ACR) in prevalence were calculated for 5-year age groups and averaged across these groups to align with the cohort duration. |
| overweight ACR adults 2015 (Dmnl/Year/Year)[35-49,upper] | 0.010178 | DHS Perú 2015-2020 | Annual Change Rates (ACR) in prevalence were calculated for 5-year age groups and averaged across these groups to align with the cohort duration. |
| overweight ACR adults 2020 (Dmnl/Year/Year)[15-24,lower] | -0.0012 | DHS Perú 2020 and 2023 | Annual Change Rates (ACR) in prevalence were calculated for 5-year age groups and averaged across these groups to align with the cohort duration. |
| overweight ACR adults 2020 (Dmnl/Year/Year)[15-24,upper] | 0.002997 | DHS Perú 2020 and 2023 | Annual Change Rates (ACR) in prevalence were calculated for 5-year age groups and averaged across these groups to align with the cohort duration. |
| overweight ACR adults 2020 (Dmnl/Year/Year)[25-34,lower] | -0.01329 | DHS Perú 2020 and 2023 | Annual Change Rates (ACR) in prevalence were calculated for 5-year age groups and averaged across these groups to align with the cohort duration. |
| overweight ACR adults 2020 (Dmnl/Year/Year)[25-34,upper] | 0.002696 | DHS Perú 2020 and 2023 | Annual Change Rates (ACR) in prevalence were calculated for 5-year age groups and averaged across these groups to align with the cohort duration. |
| overweight ACR adults 2020 (Dmnl/Year/Year)[35-49,lower] | -0.00396 | DHS Perú 2020 and 2023 | Annual Change Rates (ACR) in prevalence were calculated for 5-year age groups and averaged across these groups to align with the cohort duration. |
| overweight ACR adults 2020 (Dmnl/Year/Year)[35-49,upper] | -0.00685 | DHS Perú 2020 and 2023 | Annual Change Rates (ACR) in prevalence were calculated for 5-year age groups and averaged across these groups to align with the cohort duration. |
| overweight ACR children 2000 (Dmnl/Year/Year)[0-4,lower] | -0.00111 | DHS Perú 2020 and 2023 | Annual Change Rates (ACR) in prevalence were calculated for 5-year age groups and averaged across these groups to align with the cohort duration. |
| overweight ACR children 2000 (Dmnl/Year/Year)[0-4,upper] | -0.0035 | DHS Perú 2020 and 2023 | Annual Change Rates (ACR) in prevalence were calculated for 5-year age groups and averaged across these groups to align with the cohort duration. |
| overweight ACR children 2000 (Dmnl/Year/Year)[10-14,lower] | 0.003669 | NCD-RisC | Annual Change Rates (ACR) in prevalence were calculated for 5-year age groups and averaged across these groups to align with the cohort duration. |
| overweight ACR children 2000 (Dmnl/Year/Year)[10-14,upper] | 0.003669 | NCD-RisC | Annual Change Rates (ACR) in prevalence were calculated for 5-year age groups and averaged across these groups to align with the cohort duration. |
| overweight ACR children 2000 (Dmnl/Year/Year)[5-9,lower] | 0.004378 | NCD-RisC | Annual Change Rates (ACR) in prevalence were calculated for 5-year age groups and averaged across these groups to align with the cohort duration. |
| overweight ACR children 2000 (Dmnl/Year/Year)[5-9,upper] | 0.004378 | NCD-RisC | Annual Change Rates (ACR) in prevalence were calculated for 5-year age groups and averaged across these groups to align with the cohort duration. |
| overweight ACR children 2005 (Dmnl/Year/Year)[0-4,lower] | -0.00037 | DHS Perú 2005-2010 | Annual Change Rates (ACR) in prevalence were calculated for 5-year age groups and averaged across these groups to align with the cohort duration. |
| overweight ACR children 2005 (Dmnl/Year/Year)[0-4,upper] | -0.0025 | DHS Perú 2005-2010 | Annual Change Rates (ACR) in prevalence were calculated for 5-year age groups and averaged across these groups to align with the cohort duration. |
| overweight ACR children 2005 (Dmnl/Year/Year)[10-14,lower] | 0.00673 | NCD-RisC | Annual Change Rates (ACR) in prevalence were calculated for 5-year age groups and averaged across these groups to align with the cohort duration. |
| overweight ACR children 2005 (Dmnl/Year/Year)[10-14,upper] | 0.00673 | NCD-RisC | Annual Change Rates (ACR) in prevalence were calculated for 5-year age groups and averaged across these groups to align with the cohort duration. |
| overweight ACR children 2005 (Dmnl/Year/Year)[5-9,lower] | 0.007602 | NCD-RisC | Annual Change Rates (ACR) in prevalence were calculated for 5-year age groups and averaged across these groups to align with the cohort duration. |
| overweight ACR children 2005 (Dmnl/Year/Year)[5-9,upper] | 0.007602 | NCD-RisC | Annual Change Rates (ACR) in prevalence were calculated for 5-year age groups and averaged across these groups to align with the cohort duration. |
| overweight ACR children 2010 (Dmnl/Year/Year)[0-4,lower] | 0.0021 | DHS Perú 2010-2015 | Annual Change Rates (ACR) in prevalence were calculated for 5-year age groups and averaged across these groups to align with the cohort duration. |
| overweight ACR children 2010 (Dmnl/Year/Year)[0-4,upper] | -0.00127 | DHS Perú 2010-2015 | Annual Change Rates (ACR) in prevalence were calculated for 5-year age groups and averaged across these groups to align with the cohort duration. |
| overweight ACR children 2010 (Dmnl/Year/Year)[10-14,lower] | 0.01199 | NCD-RisC | Annual Change Rates (ACR) in prevalence were calculated for 5-year age groups and averaged across these groups to align with the cohort duration. |
| overweight ACR children 2010 (Dmnl/Year/Year)[10-14,upper] | 0.01199 | NCD-RisC | Annual Change Rates (ACR) in prevalence were calculated for 5-year age groups and averaged across these groups to align with the cohort duration. |
| overweight ACR children 2010 (Dmnl/Year/Year)[5-9,lower] | 0.013067 | NCD-RisC | Annual Change Rates (ACR) in prevalence were calculated for 5-year age groups and averaged across these groups to align with the cohort duration. |
| overweight ACR children 2010 (Dmnl/Year/Year)[5-9,upper] | 0.013067 | NCD-RisC | Annual Change Rates (ACR) in prevalence were calculated for 5-year age groups and averaged across these groups to align with the cohort duration. |
| overweight ACR children 2015 (Dmnl/Year/Year)[0-4,lower] | 0.003791 | DHS Perú 2015-2020 | Annual Change Rates (ACR) in prevalence were calculated for 5-year age groups and averaged across these groups to align with the cohort duration. |
| overweight ACR children 2015 (Dmnl/Year/Year)[0-4,upper] | 0.000844 | DHS Perú 2015-2020 | Annual Change Rates (ACR) in prevalence were calculated for 5-year age groups and averaged across these groups to align with the cohort duration. |
| overweight ACR children 2015 (Dmnl/Year/Year)[10-14,lower] | 0.01651 | NCD-RisC | Annual Change Rates (ACR) in prevalence were calculated for 5-year age groups and averaged across these groups to align with the cohort duration. |
| overweight ACR children 2015 (Dmnl/Year/Year)[10-14,upper] | 0.01651 | NCD-RisC | Annual Change Rates (ACR) in prevalence were calculated for 5-year age groups and averaged across these groups to align with the cohort duration. |
| overweight ACR children 2015 (Dmnl/Year/Year)[5-9,lower] | 0.017491 | NCD-RisC | Annual Change Rates (ACR) in prevalence were calculated for 5-year age groups and averaged across these groups to align with the cohort duration. |
| overweight ACR children 2015 (Dmnl/Year/Year)[5-9,upper] | 0.017491 | NCD-RisC | Annual Change Rates (ACR) in prevalence were calculated for 5-year age groups and averaged across these groups to align with the cohort duration. |
| overweight ACR children 2020 (Dmnl/Year/Year)[0-4,lower] | -0.0062 | DHS Perú 2020 and 2023 | Annual Change Rates (ACR) in prevalence were calculated for 5-year age groups and averaged across these groups to align with the cohort duration. |
| overweight ACR children 2020 (Dmnl/Year/Year)[0-4,upper] | 0.001497 | DHS Perú 2020 and 2023 | Annual Change Rates (ACR) in prevalence were calculated for 5-year age groups and averaged across these groups to align with the cohort duration. |
| overweight ACR children 2020 (Dmnl/Year/Year)[10-14,lower] | 0.01793 | NCD-RisC | Annual Change Rates (ACR) in prevalence were calculated for 5-year age groups and averaged across these groups to align with the cohort duration. |
| overweight ACR children 2020 (Dmnl/Year/Year)[10-14,upper] | 0.01793 | NCD-RisC | Annual Change Rates (ACR) in prevalence were calculated for 5-year age groups and averaged across these groups to align with the cohort duration. |
| overweight ACR children 2020 (Dmnl/Year/Year)[5-9,lower] | 0.0185 | NCD-RisC | Annual Change Rates (ACR) in prevalence were calculated for 5-year age groups and averaged across these groups to align with the cohort duration. |
| overweight ACR children 2020 (Dmnl/Year/Year)[5-9,upper] | 0.0185 | NCD-RisC | Annual Change Rates (ACR) in prevalence were calculated for 5-year age groups and averaged across these groups to align with the cohort duration. |
| stunting ACR adults 2000 (Dmnl/Year/Year)[15-24,lower] | -0.0078 | DHS Perú 2000-2005 | Annual Change Rates (ACR) in prevalence were calculated for 5-year age groups and averaged across these groups to align with the cohort duration. |
| stunting ACR adults 2000 (Dmnl/Year/Year)[15-24,upper] | 0.002263 | DHS Perú 2000-2005 | Annual Change Rates (ACR) in prevalence were calculated for 5-year age groups and averaged across these groups to align with the cohort duration. |
| stunting ACR adults 2005 (Dmnl/Year/Year)[15-24,lower] | 0.001308 | DHS Perú 2005-2010 | Annual Change Rates (ACR) in prevalence were calculated for 5-year age groups and averaged across these groups to align with the cohort duration. |
| stunting ACR adults 2005 (Dmnl/Year/Year)[15-24,upper] | -0.00347 | DHS Perú 2005-2010 | Annual Change Rates (ACR) in prevalence were calculated for 5-year age groups and averaged across these groups to align with the cohort duration. |
| stunting ACR adults 2010 (Dmnl/Year/Year)[15-24,lower] | -0.00249 | DHS Perú 2010-2015 | Annual Change Rates (ACR) in prevalence were calculated for 5-year age groups and averaged across these groups to align with the cohort duration. |
| stunting ACR adults 2010 (Dmnl/Year/Year)[15-24,upper] | -0.00202 | DHS Perú 2010-2015 | Annual Change Rates (ACR) in prevalence were calculated for 5-year age groups and averaged across these groups to align with the cohort duration. |
| stunting ACR adults 2015 (Dmnl/Year/Year)[15-24,lower] | -0.00391 | DHS Perú 2015-2020 | Annual Change Rates (ACR) in prevalence were calculated for 5-year age groups and averaged across these groups to align with the cohort duration. |
| stunting ACR adults 2015 (Dmnl/Year/Year)[15-24,upper] | -0.00235 | DHS Perú 2015-2020 | Annual Change Rates (ACR) in prevalence were calculated for 5-year age groups and averaged across these groups to align with the cohort duration. |
| stunting ACR adults 2020 (Dmnl/Year/Year)[15-24,lower] | -0.00331 | DHS Perú 2020-2023 | Annual Change Rates (ACR) in prevalence were calculated for 5-year age groups and averaged across these groups to align with the cohort duration. |
| stunting ACR adults 2020 (Dmnl/Year/Year)[15-24,upper] | -0.00081 | DHS Perú 2020-2023 | Annual Change Rates (ACR) in prevalence were calculated for 5-year age groups and averaged across these groups to align with the cohort duration. |
| stunting ACR children 2000 (Dmnl/Year/Year)[lower] | 0.002765 | DHS Perú 2000-2005 | Annual Change Rates (ACR) in prevalence were calculated for 5-year age groups and averaged across these groups to align with the cohort duration. |
| stunting ACR children 2000 (Dmnl/Year/Year)[upper] | -0.00326 | DHS Perú 2000-2005 | Annual Change Rates (ACR) in prevalence were calculated for 5-year age groups and averaged across these groups to align with the cohort duration. |
| stunting ACR children 2005 (Dmnl/Year/Year)[lower] | -0.02448 | DHS Perú 2005-2010 | Annual Change Rates (ACR) in prevalence were calculated for 5-year age groups and averaged across these groups to align with the cohort duration. |
| stunting ACR children 2005 (Dmnl/Year/Year)[upper] | -0.01066 | DHS Perú 2005-2010 | Annual Change Rates (ACR) in prevalence were calculated for 5-year age groups and averaged across these groups to align with the cohort duration. |
| stunting ACR children 2010 (Dmnl/Year/Year)[lower] | -0.02048 | DHS Perú 2010-2015 | Annual Change Rates (ACR) in prevalence were calculated for 5-year age groups and averaged across these groups to align with the cohort duration. |
| stunting ACR children 2010 (Dmnl/Year/Year)[upper] | -0.01263 | DHS Perú 2010-2015 | Annual Change Rates (ACR) in prevalence were calculated for 5-year age groups and averaged across these groups to align with the cohort duration. |
| stunting ACR children 2015 (Dmnl/Year/Year)[lower] | -0.01667 | DHS Perú 2015-2020 | Annual Change Rates (ACR) in prevalence were calculated for 5-year age groups and averaged across these groups to align with the cohort duration. |
| stunting ACR children 2015 (Dmnl/Year/Year)[upper] | -0.00429 | DHS Perú 2015-2020 | Annual Change Rates (ACR) in prevalence were calculated for 5-year age groups and averaged across these groups to align with the cohort duration. |
| stunting ACR children 2020 (Dmnl/Year/Year)[lower] | -0.00166 | DHS Perú 2020-2023 | Annual Change Rates (ACR) in prevalence were calculated for 5-year age groups and averaged across these groups to align with the cohort duration. |
| stunting ACR children 2020 (Dmnl/Year/Year)[upper] | 0.002113 | DHS Perú 2020-2023 | Annual Change Rates (ACR) in prevalence were calculated for 5-year age groups and averaged across these groups to align with the cohort duration. |
| TIME STEP (Year ) | 0.125 | NA | NA |

Subscript refers to subgroup, i.e. age and/or socioeconomic group (lower for poorer individuals and upper for richer individuals). ACR = Annual Change Rate. DHS = Demographic and Health Surveys. LBW = Low Birth Weight. NCD-RisC = Non-Communicable Disease Risk Factor Collaboration. OR = Odds Ratio. RR = Relative Risk. Dmnl: Dimensionless. TP: Transition Probability .

**References**

1.	Rullier HB, Ramirez RR, Tanco ED, Ochoa JGV, Cruz LMS. Peru: Situacion y perspectivas de la mortalidad por sexo y grupo de edad, nacional y por departamentos, 1990-2025 (Methodologia y tablas de mortalidad). Lima, Peru: INEI, WHO; 2010.

2.	WHO. Global Health Observatory data repository. Low Birth Weight. Data by country 2023. Available from: <https://www.who.int/data/gho/legacy?lang=en>.

3.	Zanabria JAG, Aguilar AS, Calle NH, Espino CG, Tanco ED, Cruz LMS. Peru: Estimaciones y Proyecciones de la Poblacion nacional, por ano calendario y edad simple, 1950-2050. Lima, Peru: INEI, UNFPA, CEPAL; 2019.

4.	Christian P, Lee SE, Donahue Angel M, Adair LS, Arifeen SE, Ashorn P, et al. Risk of childhood undernutrition related to small-for-gestational age and preterm birth in low- and middle-income countries. Int J Epidemiol. 2013;42(5):1340-55. Epub 20130806. doi: 10.1093/ije/dyt109. PubMed PMID: 23920141; PubMed Central PMCID: PMCPMC3816349.

5.	Kozuki N, Katz J, Lee AC, Vogel JP, Silveira MF, Sania A, et al. Short Maternal Stature Increases Risk of Small-for-Gestational-Age and Preterm Births in Low- and Middle-Income Countries: Individual Participant Data Meta-Analysis and Population Attributable Fraction. J Nutr. 2015;145(11):2542-50. Epub 20150930. doi: 10.3945/jn.115.216374. PubMed PMID: 26423738; PubMed Central PMCID: PMCPMC6457093.
