# Supplementary material for: Retrofitting fiscal policies to reduce the double burden of malnutrition in Peru: A system dynamics modelling study
Source: PLoS Med. 2026 Aug 13;23(8):e1005167. doi: 10.1371/journal.pmed.1005167 (PMC13472416; doi:10.1371/journal.pmed.1005167)
Supplement: Supplementary file 4 [file pmed.1005167.s004.docx]

**S1 Figure. Causal loop diagram representing a high-level structure of main feedback mechanisms comprising the system dynamics model used in this study**

B: Balancing Feedback Loop; R: Reinforcing Feedback Loop; SSB: Sugar-sweetened beverages; UPF: Ultra-processed foods.

The + sign in the arrows represents positive polarity, i.e. the arrow indicates change in the same direction.

The - sign in the arrows represents negative polarity, i.e. the arrow indicates change in opposite directions.
