# Supplementary material for: Retrofitting fiscal policies to reduce the double burden of malnutrition in Peru: A system dynamics modelling study
Source: PLoS Med. 2026 Aug 13;23(8):e1005167. doi: 10.1371/journal.pmed.1005167 (PMC13472416; doi:10.1371/journal.pmed.1005167)
Supplement: Supplementary file 5 [file pmed.1005167.s005.docx]

**S2 Table . Overview of revenue reallocation scenarios**

| Policy name | Revenue reallocation scenario | Scenario description | Outcomes targeted | Effect estimates | Current policy in Peru |
| --- | --- | --- | --- | --- | --- |
| *Juntos Basic Transfer* | **A:** expand coverage | Increasing the number of households eligible for the 100 soles per month transfer, starting with those in the lowest socioeconomic quintile before extending coverage to households in higher quintiles as tax revenue allows. | Stunting and overweight in children (0-14 years) in the high socioeconomic group | overweight = - 0.0296  stunting = -0.0523 | Since 2005, 100 soles per month are given to households in extreme poverty with pregnant women and / or children up to age 19. Transfer is conditional on attending health care check-ups for children < 5 yrs, pregnant or lactating women, and attending more than >85% of all school lessons for children > 5 yrs. By 2023, 762,000 households were receiving *Basic Transfer*. |
|  | **B:** increase cash | Raising the cash amount provided to current beneficiaries of the *Basic Transfer* with amount based on available tax revenue. | Stunting and overweight in children (0-14 years) | overweight = -0.0006  stunting = -0.0009 |  |
| *Juntos Early Childhood Transfer* | **C:** expand coverage | Extending the 50 soles transfer to more households with children under five, starting with those in the lowest socioeconomic quintile before extending coverage to households in higher quintiles as tax revenue allows. | Stunting in children under-5 years | stunting = -0.065 | Since 2021, additional 50 soles are given to households with pregnant women and children under five, conditional on pregnant women attending scheduled appointments for the comprehensive package of health services, children aged 0-35 months attending appointments to receive the comprehensive package of health services, and children aged 36-59 months being enrolled and attending preschool or early childhood education. |
| Fruit and vegetable subsidies | **D:** subsidise fruit and vegetables | Additional subsidy for fruits and vegetables, with the subsidy amount determined by available tax revenue. | Stunting in children (0-14) and overweight in adults and children (0-14) | overweight = - 0.435  stunting = -0.593 | Fruit and vegetables are exempt from Value Added Tax (VAT) for both sale and import (Law No. 31651)^1^. |

Column “Scenario description” details how scenarios were incorporated into the model. All scenarios started in 2024. Column “Current policy in Peru” provides a description of each policy as currently implemented in Peru. Effect estimates refer to % increase in overweight or stunting per year for 1 unit increase in each revenue reallocation scenario. For further details on effect estimates sources and interpretation refer to S3 Table.

^1.^LEY QUE PRORROGA LA VIGENCIA DE LOS APÉNDICES I Y II DE LA LEY DEL IMPUESTO GENERAL A LAS VENTAS E IMPUESTO SELECTIVO AL CONSUMO, LEY 31651 (2022).
