# Supplementary material for: Retrofitting fiscal policies to reduce the double burden of malnutrition in Peru: A system dynamics modelling study
Source: PLoS Med. 2026 Aug 13;23(8):e1005167. doi: 10.1371/journal.pmed.1005167 (PMC13472416; doi:10.1371/journal.pmed.1005167)
Supplement: Supplementary file 6 [file pmed.1005167.s006.docx]

**S3 Table. Exogenous model parameter values and their data sources for the policy scenario modelling.**

| Variable (unit) | value | Source | Note |
| --- | --- | --- | --- |
| annual F and V change rate (soles/(Year*Year*people)) | 8.92542 | Euromonitor | Annual change rate of fruit and vegetable (F and V) sales between 2010 and 2023 calculated using data from Euromonitor |
| average children Juntos participants | 3.333 | Perez-Lu JE, Carcamo C, Nandi A, Kaufman JS. Health effects of 'Juntos', a conditional cash transfer programme in Peru. Matern Child Nutr 2017; 13(3): 07. [1] | Number of children in recipient households adjusted to cohort duration of children in model (children under 15) |
| Baseline TPI recipients hh | 94319 | Ministry of Development and Social Inclusion – Press Release[2] | Number of households that received the Early Childhood Transfer (TPI) payment in 2023, where the policy was first implemented. No households received TPI before the policy implementation. In the baseline scenario, we assumed that the number of beneficiaries did not change after 2023. |
| Baseline TB recipient hh | 0, 0, 0, 0, 0, 22550, 163742, 353067, 420491, 409610, 471511, 474064, 619723, 648734, 755556, 769158, 668030, 693504, 693980, 678810, 699751, 682099, 688254 |  | Number of households that received the Juntos Basic Transfer (TB) in each year of the simulation until 2022. In the baseline scenario, we assumed that the number of beneficiaries did not change after 2022. |
| baseline TB cash transferred per hh (soles/(Year*household)) | 1200 | Andersen CT, Reynolds SA, Behrman JR, et al. Participation in the Juntos Conditional Cash Transfer Program in Peru Is Associated with Changes in Child Anthropometric Status but Not Language Development or School Achievement. J Nutr 2015; 145(10): 2396-405. [3] | Cash transferred to eligible households under the Juntos Basic Transfer (TB) under the baseline scenario. Annual estimate for the amount of 100 soles per household per month. |
| baseline TPI costs per hh (soles/(Year*household)) | 600 | MIDIS. Evaluación de impacto de la transferencia a primera infancia (TPI) sobre la cobertura del paquete integral de salud, la prevalencia de anemia y desnutrición crónica infantil. 2024. https://evidencia.midis.gob.pe/evaluacion-transferencia-primera-infancia-juntos (accessed November 30 2024). [4] | Cash transferred to eligible households under the Juntos Early Childhood Transfer (TPI) under the baseline scenario. Annual estimate for the amount of 50 soles per household per month. |
| cross price elasticity F and V on SSB demand (Dmnl/Year) | 0.0675 | Pereda PC, Moz-Christofoletti MA, Duran AC, Louzada MLaC, Ng SW. Equity-Driven fiscal Policies: Taxing Ultra-Processed products and subsidising minimally processed foods. Food Policy 2024; 126. [5] | Estimates from Mexico. Average of cross-price elasticity for fruit and for vegetables. |
| cross price elasticity F and V on UPF demand (Dmnl/Year) | 0.0645 | Pereda PC, Moz-Christofoletti MA, Duran AC, Louzada MLaC, Ng SW. Equity-Driven fiscal Policies: Taxing Ultra-Processed products and subsidising minimally processed foods. Food Policy 2024; 126. [5] | Estimates from Mexico.  Average of cross-price elasticity for fruit and for vegetables. |
| cross price elasticity SSB on UPF demand (Dmnl/Year) | -0.02 | Pereda PC, Moz-Christofoletti MA, Duran AC, Louzada MLaC, Ng SW. Equity-Driven fiscal Policies: Taxing Ultra-Processed products and subsidising minimally processed foods. Food Policy 2024; 126. [5] | Estimates from Mexico |
| cross price elasticity UPF on SSB demand (Dmnl/Year) | 0.009 | Pereda PC, Moz-Christofoletti MA, Duran AC, Louzada MLaC, Ng SW. Equity-Driven fiscal Policies: Taxing Ultra-Processed products and subsidising minimally processed foods. Food Policy 2024; 126. [5] | Estimates from Mexico |
| current SSB excise tax | 0.25 | Lowery CM, Saavedra-Garcia L, Diez-Canseco F, Cárdenas MK, Miranda JJ, Taillie LS. Sugar-sweetened beverage purchases in urban Peru before the implementation of taxation and warning label policies: a baseline study. BMC Public Health 2022; 22(1): 2389. [6] | Current excise tax on SSBs with more than 6g of sugar per 100 ml in Peru. |
| EE F and V prices on overweight (Dmnl/Year) | - 0.435 | Han E, Powell LM. Effect of food prices on the prevalence of obesity among young adults. *Public Health* 2011; **125**(3): 129-35. [7] | Effect estimate (EE) of fruit and vegetable (F and V) price change on overweight. Estimates from the USA from studies between 1992 and 2003. Using the average elasticity of obesity for men and women. Elasticity is negative to indicate decrease. We assume that effect on obesity is the same as the effect on overweight. |
| EE F and V prices on stunting (Dmnl/Year) | -0.59333 | Headey DD, Alderman HH. The Relative Caloric Prices of Healthy and Unhealthy Foods Differ Systematically across Income Levels and Continents. J Nutr 2019; 149(11): 2020-33. [8] | Effect estimate (EE) of fruit and vegetable (F and V) price change compared to price of staple products on stunting.  Averaged across vegetables, the estimate assumes stable staple prices. Under this assumption, a 1% rise in F and V prices has the same impact on stunting as a 1% increase in their relative price to staples, as substitution dynamics remain unchanged. |
| EE Juntos cash on overweight (Dmnl/Year) | -0.0006 | Fernald LCH, Gertler PJ, Neufeld LM. Role of cash in conditional cash transfer programmes for child health, growth, and development: an analysis of Mexico's Oportunidades. Lancet 2008; 371(9615): 828-37. [9] | Effect estimate (EE) of increasing annual amount of cash transfer by 1% on overweight. Estimate from Mexico. (estimated adjusted to represent annual effect by dividing it with the total number of follow-up years, n=6) |
| EE Juntos cash on stunting (Dmnl/Year) | -0.0009 | Fernald LCH, Gertler PJ, Neufeld LM. Role of cash in conditional cash transfer programmes for child health, growth, and development: an analysis of Mexico's Oportunidades. Lancet 2008; 371(9615): 828-37. [9] | Effect estimate (EE) of increasing annual amount of cash transfer by 1% on stunting. Estimate from Mexico. |
| EE Juntos coverage on overweight children (Dmnl/Year) | -0.02963 | Andersen CT, Reynolds SA, Behrman JR, et al. Participation in the Juntos Conditional Cash Transfer Program in Peru Is Associated with Changes in Child Anthropometric Status but Not Language Development or School Achievement. J Nutr 2015; 145(10): 2396-405. [3] | Effect estimate (EE) of 1% increase in Juntos coverage on overweight (estimated adjusted to represent annual effect by dividing it with the total number of follow-up years, n=3) |
| EE Juntos coverage on stunting (Dmnl/Year) | -0.05229 | Andersen CT, Reynolds SA, Behrman JR, et al. Participation in the Juntos Conditional Cash Transfer Programme in Peru Is Associated with Changes in Child Anthropometric Status but Not Language Development or School Achievement. J Nutr 2015; 145(10): 2396-405. [3] | Effect estimate (EE) of 1% increase in Juntos coverage on stunting |
| EE Juntos TPI expansion (Dmnl/Year) | -0.065 | MIDIS. Evaluación de impacto de la transferencia a primera infancia (TPI) sobre la cobertura del paquete integral de salud, la prevalencia de anemia y desnutrición crónica infantil. 2024. <https://evidencia.midis.gob.pe/evaluacion-transferencia-primera-infancia-juntos> (accessed November 30 2024). [4] | Effect estimate (EE) of expanding Juntos TPI by 1% on stunting |
| EE SSB consumption on overweight (Dmnl/Year) | 0.00542 | Ferretti F, Mariani M. Sugar-sweetened beverage affordability and the prevalence of overweight and obesity in a cross section of countries. Global Health 2019; 15(1): 30. [10] | Effect estimate (EE) of SSB consumption on overweight |
| EE UPF consumption on overweight (Dmnl/Year) | 0.00542 | Ferretti F, Mariani M. Sugar-sweetened beverage affordability and the prevalence of overweight and obesity in a cross section of countries. Global Health 2019; 15(1): 30. [10] | Same effect estimate (EE) used as for SSB effect on overweight, due to unavailability of data on UPFs. |
| F and V market size 2010 (soles/(Year*people)) | 356.965 | Euromonitor |  |
| hh size Juntos participants | 4.9 | Perez-Lu JE, Carcamo C, Nandi A, Kaufman JS. Health effects of 'Juntos', a conditional cash transfer programme in Peru. Matern Child Nutr 2017; 13(3): 07.[1] | Average household size for Juntos participants, 2007-2013  Assumption that household size for Juntos participants did not change over time |
| average hh size Peru (people/household) | 3.5 | Source: Patterns and trends in household size and composition: Evidence from a United Nations dataset[11] | Average household size in Peru in 2012  Assumption that average household size in Peru did not change over time |
| Market size SSB Peru 2000 (soles/(people*Year)) | 117.682 | Euromonitor | Total retail sales data for Peru in 2000 was estimated using 2010 figures from Euromonitor and back-calculated based on annual growth rate, as outlined in PAHO, 2015[12] |
| Market size SSB Peru 2024 (soles/(people*Year)) | 366.596 | Euromonitor |  |
| proportion benefitting from F and V subsidies (Dmnl)[upper] | 1 | Hernández-Vásquez A, Visconti Lopez FJ, Vargas-Fernández R. Socioeconomic inequalities in the consumption of fruits and vegetables in Peru between 2014 and 2019. Public Health Nutr 2022; 25(12): 1-11. [13] | Proportion of individuals within the richest 80% of the population who benefit from fruit and vegetable (F and V) subsidies |
| proportion benefitting from F and V subsidies (Dmnl)[lower] | 0.376754 | Hernández-Vásquez A, Visconti Lopez FJ, Vargas-Fernández R. Socioeconomic inequalities in the consumption of fruits and vegetables in Peru between 2014 and 2019. Public Health Nutr 2022; 25(12): 1-11. [13] | Proportion of individuals within the poorest 20% of the population who benefit from fruit and vegetable (F and V) subsidies. It is calculated by dividing the proportion of poor individuals eating adequate F and V by the proportion of wealthier individuals eating adequate F and V. |
| proportion in lowest and upper wealth quartile adults (Dmnl)[upper] | 0.8036 | DHS, Peru | Proportion of adults living in 80% of wealthiest households, calculated across age groups and years, since there were no significant differences |
| proportion in lowest and upper wealth quartile adults (Dmnl)[lower] | 0.1964 | DHS, Peru | Proportion of adults living in 20% of poorest households, calculated across age groups and years, since there were no significant differences |
| proportion in lowest and upper wealth quartile children (Dmnl)[upper] | 0.7106 | DHS, Peru | Proportion of children living in 80% of wealthiest households, calculated across age groups and years, since there were no significant differences |
| proportion in lowest and upper wealth quartile children (Dmnl)[lower] | 0.2894 | DHS, Peru | Proportion of children living in 20% of poorest households, calculated across age groups and years, since there were no significant differences |
| proportion taxable drinks (Dmnl) | 0.903226 | Lowery CM, Saavedra-Garcia L, Diez-Canseco F, Cárdenas MK, Miranda JJ, Taillie LS. Sugar-sweetened beverage purchases in urban Peru before the implementation of taxation and warning label policies: a baseline study. BMC Public Health 2022; 22(1): 2389.[6] | Proportion of drinks in Peru that are taxed under the baseline scenario, based on a study conducted before the implementation of taxation and warning label policies in Peru, estimated as the ratio of average monthly purchase volume of high-sugar beverages over the average monthly purchase volume of high and low-sugar beverages |
| SSB goal market size (soles/(people*Year)) | 668.738 | Euromonitor | Average per capita SSB Market size across Latin America for 2024, following currency conversion. SSB defined according to PAHO, 2015[12] |
| SSB own price elasticity (Dmnl/Year)[upper] | -1.201 | Paraje G. The Effect of Price and Socioeconomic Level on the Consumption of Sugar-Sweetened Beverages (SSB): The Case of Ecuador. PLoS One 2016; 11(3): e0152260. [14] | Study from Ecuador, value for the upper three quintiles. |
| SSB own price elasticity (Dmnl/Year)[lower] | -1.328 | Paraje G. The Effect of Price and Socio-Economic Level on the Consumption of Sugar-Sweetened Beverages (SSB): The Case of Ecuador. PLoS One 2016; 11(3): e0152260. [14] | Study from Ecuador, value for the lowest two quintiles. |
| subsidy fade out time (Year) | 7 |  | Subsidy was assumed to fade out over seven years, consistent with tax fade out time |
| tax fade out time (Year) | 7 | Berezvai Z, Vitrai J, Tóth G, Brys Z, Bakacs M, Joó T. Long-term impact of unhealthy food tax on consumption and the drivers behind: A longitudinal study in Hungary. Health Policy 2024; 146: 105098. [15] | Tax was assumed to fade out over seven years, according to prior research from Hungary |
| policy peak time | 2 | Colchero MA, Rivera-Dommarco J, Popkin BM, Ng SW. In Mexico, Evidence Of Sustained Consumer Response Two Years After Implementing A Sugar-Sweetened Beverage Tax. Health Aff (Millwood) 2017; 36(3): 564-71. [16] | Assumption that tax and subsidy effects peaked after two years to reflect an adjustment period, according to prior research on Sugar-Sweetened Beverage taxation in Mexico |
| time to close SSB market size gap (Year) | 40 | NA | Assumption that the Peruvian market will reach the Latin American average values for 2024 by the year 2040. |
| time to close UPF market size gap (Year) | 40 | NA | Assumption that the Peruvian market will reach the Latin American average values for 2024 by the year 2040. |
| UPF goal market size (soles/(people*Year)) | 535.72 | Euromonitor | Average per capita UPF Market size across Latin America for 2024, following currency conversion. UPF defined according to PAHO, 2015[12] |
| UPF market size 2000 (soles/(Year*people)) | 84.4229 | Euromonitor | Total retail sales data for Peru in 2000 was estimated using 2010 figures from Euromonitor and back-calculated based on annual growth rate, as outlined in PAHO 2015[12] |
| UPF market size 2024 (soles/(Year*people)) | 290 | Euromonitor | Total UPF retail sales for 2024, UPF defined according to PAHO 2015[12] |
| UPF own price elasticity (Dmnl/Year)[upper] | -1.201 | Paraje G. The Effect of Price and Socio-Economic Level on the Consumption of Sugar-Sweetened Beverages (SSB): The Case of Ecuador. PLoS One 2016; 11(3): e0152260. | Own price elasticity for ultra-processed foods (UPF) for the wealthiest 80% of the population. We used the same estimates as for SSB, as there are no SES stratified estimates for UPF. Other non-stratified price elasticities for UPF fall within a similar range. |
| UPF own price elasticity (Dmnl/Year)[lower] | -1.328 | Paraje G. The Effect of Price and Socio-Economic Level on the Consumption of Sugar-Sweetened Beverages (SSB): The Case of Ecuador. PLoS One 2016; 11(3): e0152260. | Own price elasticity for ultra-processed foods (UPF) for the poorest 20% of the population. We used the same estimates as for SSB, as there are no SES stratified estimates for UPF. Other non-stratified price elasticities for UPF fall within a similar range. |
| cash devaluation since start TPI (Dmnl) | 0.939, 0.909, 0.88, 0.852, 0.825, 0.799, 0.773, 0.748, 0.724, 0.701, 0.679, 0.657, 0.636, 0.616, 0.596, 0.577, 0.559, 0.541 | Macrobond, accessed through <https://www.focus-economics.com/country-indicator/peru/inflation/>  [17] | Values of cash devaluation for each year of the simulation from the start of the Juntos Early Childhood Transfer (TPI), in 2022, until the end of the simulation. We use observed value for 2023 and then a 10 year average for the following years |
| cash devaluation since start TB (Dmnl) | 0.984, 0.965, 0.948, 0.896, 0.87, 0.857, 0.829, 0.8, 0.779, 0.753, 0.728, 0.703, 0.683, 0.673, 0.658, 0.645, 0.619, 0.571, 0.536, 0.519, 0.503, 0.487, 0.471, 0.456, 0.442, 0.427, 0.414, 0.401, 0.388, 0.375, 0.363, 0.352, 0.341, 0.33, 0.319, 0.309 | Macrobond, accessed through <https://www.focus-economics.com/country-indicator/peru/inflation/> [17] | Values of cash devaluation for each year from the start of Juntos Basic Transfer (TB), in 2005, until the end of the simulation. We use observed value for 2023 and then a 10 year average for the following years |
| SSB tax (Dmnl) | 0.25 |  | Current tax applied to SSBs with >6g/100mL |
| UPF tax (Dmnl) | 0.1 |  | Tax assumed for UPFs under Scenario 2 |
| Allocation time (Year) | 1 |  | Indicates that revenue is allocated once per year |

Dmnl = dimensionless. EE = effect estimate. F and V = fruit and vegetables. hh = household. Lower = represents the lowest wealth quintile. SSB = sugar-sweetened beverage. TB = *Basic Transfer* of Juntos. TPI = *Early Childhood Transfer* of Juntos. UPF = ultra-processed food. Upper = represents the upper four wealth quintiles.

**References**

1.	Perez-Lu JE, Carcamo C, Nandi A, Kaufman JS. Health effects of 'Juntos', a conditional cash transfer programme in Peru. Matern Child Nutr. 2017;13(3):07. doi: <https://dx.doi.org/10.1111/mcn.12348>. PubMed PMID: 27549365.

2.	Press Office, Juntos Programme, Ministry of Development and Social Inclusion. Juntos iniciará pago a más de 675 mil familias usuarias a nivel nacional 2023 [26/10/2025]. Press Release]. Available from: <https://www.gob.pe/institucion/juntos/noticias/789751-juntos-iniciara-pago-a-mas-de-675-mil-familias-usuarias-a-nivel-nacional>.

3.	Andersen CT, Reynolds SA, Behrman JR, Crookston BT, Dearden KA, Escobal J, et al. Participation in the Juntos Conditional Cash Transfer Program in Peru Is Associated with Changes in Child Anthropometric Status but Not Language Development or School Achievement<sup>1-4</sup>. J Nutr. 2015;145(10):2396-405. doi: <http://dx.doi.org/10.3945/jn.115.213546>. PubMed PMID: 606519098.

4.	social P-MdDeI. Evaluación de impacto de la transferencia a primera infancia (TPI) sobre la cobertura del paquete integral de salud, la prevalencia de anemia y desnutrición crónica infantil 2024 [cited 2024 November 30]. Available from: <https://evidencia.midis.gob.pe/evaluacion-transferencia-primera-infancia-juntos/>.

5.	Pereda PC, Moz-Christofoletti MA, Duran AC, Louzada MLaC, Ng SW. Equity-Driven fiscal Policies: Taxing Ultra-Processed products and subsidizing minimally processed foods. Food Policy. 2024.

6.	Lowery CM, Saavedra-Garcia L, Diez-Canseco F, Cárdenas MK, Miranda JJ, Taillie LS. Sugar-sweetened beverage purchases in urban Peru before the implementation of taxation and warning label policies: a baseline study. BMC Public Health. 2022;22(1):2389. Epub 20221220. doi: 10.1186/s12889-022-14762-w. PubMed PMID: 36539775; PubMed Central PMCID: PMCPMC9764463.

7.	Han E, Powell LM. Effect of food prices on the prevalence of obesity among young adults. Public Health. 2011;125(3):129-35. Epub 20110126. doi: 10.1016/j.puhe.2010.11.014. PubMed PMID: 21272902.

8.	Headey DD, Alderman HH. The Relative Caloric Prices of Healthy and Unhealthy Foods Differ Systematically across Income Levels and Continents. J Nutr. 2019;149(11):2020-33. doi: 10.1093/jn/nxz158. PubMed PMID: 31332436; PubMed Central PMCID: PMCPMC6825829.

9.	Fernald LCH, Gertler PJ, Neufeld LM. Role of cash in conditional cash transfer programmes for child health, growth, and development: an analysis of Mexico's Oportunidades. Lancet. 2008;371(9615):828-37. doi: 10.1016/s0140-6736(08)60382-7. PubMed PMID: WOS:000253866500030.

10.	Ferretti F, Mariani M. Sugar-sweetened beverage affordability and the prevalence of overweight and obesity in a cross section of countries. Global Health. 2019;15(1):30. Epub 20190418. doi: 10.1186/s12992-019-0474-x. PubMed PMID: 30999931; PubMed Central PMCID: PMCPMC6472017.

11.	Esri. Peru Average Household Size 2021. Available from: <https://www.arcgis.com/home/item.html?id=fc112ecdf46d4c8fae32a7ffe1914c0d>.

12.	PAHO. Ultra-processed food and drink products in Latin America: Trends, impact on obesity, policy implications. 2015.

13.	Hernández-Vásquez A, Visconti Lopez FJ, Vargas-Fernández R. Socio-economic inequalities in the consumption of fruits and vegetables in Peru between 2014 and 2019. Public Health Nutr. 2022;25(12):1-11. Epub 20220908. doi: 10.1017/s1368980022001860. PubMed PMID: 36073028; PubMed Central PMCID: PMCPMC9991701.

14.	Paraje G. The Effect of Price and Socio-Economic Level on the Consumption of Sugar-Sweetened Beverages (SSB): The Case of Ecuador. PLoS One. 2016;11(3):e0152260. Epub 20160330. doi: 10.1371/journal.pone.0152260. PubMed PMID: 27028608; PubMed Central PMCID: PMCPMC4814055.

15.	Berezvai Z, Vitrai J, Tóth G, Brys Z, Bakacs M, Joó T. Long-term impact of unhealthy food tax on consumption and the drivers behind: A longitudinal study in Hungary. Health Policy. 2024;146:105098. Epub 20240530. doi: 10.1016/j.healthpol.2024.105098. PubMed PMID: 38851004.

16.	Colchero MA, Rivera-Dommarco J, Popkin BM, Ng SW. In Mexico, Evidence Of Sustained Consumer Response Two Years After Implementing A Sugar-Sweetened Beverage Tax. Health Aff (Millwood). 2017;36(3):564-71. Epub 20170222. doi: 10.1377/hlthaff.2016.1231. PubMed PMID: 28228484; PubMed Central PMCID: PMCPMC5442881.

17.	FOCUSECONOMICS. Peru Inflation Data 2024. Available from: <https://www.focus-economics.com/country-indicator/peru/inflation/>.
