# Supplementary material for: Retrofitting fiscal policies to reduce the double burden of malnutrition in Peru: A system dynamics modelling study
Source: PLoS Med. 2026 Aug 13;23(8):e1005167. doi: 10.1371/journal.pmed.1005167 (PMC13472416; doi:10.1371/journal.pmed.1005167)
Supplement: Supplementary file 7 [file pmed.1005167.s007.docx]

**S3 Text. Behaviour Reproduction**

To evaluate the model’s ability to replicate observed trends, we compared simulated data with observed data from 2000-2020 for total population numbers (children and adults) and the prevalence of overweight and stunting, both overall and stratified by wealth group using data from the Instituto Nacional de Estadística e Informática (INEI) and Demographic and Health Surveys Peru (DHS)/Encuesta Demográfica y de Salud Familiar (ENDES). Overweight and stunting data for children aged 5-15 was unavailable at the national level, limiting comparison for this age group.

| **Variable** | **Results** | **Observations** |
| --- | --- | --- |
| Total adult population |  | The simulation effectively captures trends in the total adult population. Observed data show a slower rise from 2005 – 2015, followed by a sharper increase to 2020, while the simulation shows steadier growth over time, aligning with observed data by 2020. This variation is likely attributable to the reliance on 5-year estimates for mortality and fertility changes, which provide adequate precision for the model's purpose. |
| Total child population |  | The simulation closely mirrors the patterns observed in the total child population, showing minimal differences in behaviour or numerical values. |
| Under five overweight |    | Similar to the observed trends, the model simulated a relatively low and stable prevalence of overweight among children under five, with slightly higher rates in the upper wealth quintiles compared to the lowest. |
| Adult overweight |    | Among adults, the simulated overweight prevalence closely matches observed trends, showing an initial slight lag, followed by a steep rise and eventual plateau, with the gap between upper and lowest wealth groups narrowing over time. However, the increase in overweight prevalence occurs slightly later in the model compared to the observed data. |
| Under five stunting |    | For stunting among children under five, the model mirrors observed patterns, showing a sharp decline between 2008 and 2015, followed by a plateau. The lowest wealth group plateaus at a higher level than the upper group, consistent with observed data. Nonetheless, some numerical differences are notable, particularly in stunting prevalence among poorer groups between 2000 and 2005. For this period, no observed data from the DHS is available, which hinders comparisons. |
