# Supplementary material for: Retrofitting fiscal policies to reduce the double burden of malnutrition in Peru: A system dynamics modelling study
Source: PLoS Med. 2026 Aug 13;23(8):e1005167. doi: 10.1371/journal.pmed.1005167 (PMC13472416; doi:10.1371/journal.pmed.1005167)
Supplement: Supplementary file 8 [file pmed.1005167.s008.docx]

**S4 Text. Model equations**

| **Variable name** | **Units** | **Equation** | **Comments** |
| --- | --- | --- | --- |
| *Auxiliary (Endogenous) variables* | | | |
| adult mortality rate | Dmnl/Year | adult mortality rate[adult age] = Baseline adult mortality rate[ adult age]+RAMP( adult mortality rate annual change[ adult age], INITIAL TIME, INITIAL TIME+20) | Assumption: Mortality rate changes between 2000 and 2020, according to observed data. After 2020, it does not change. |
| adult overweight prevalence by age | Dmnl | adult overweight prevalence by age[adult age] = SUM( overweight adults[ adult age, wealth!])/SUM( adults sb[ adult age, wealth!]) |  |
| adult overweight prevalence by wealth | Dmnl | adult overweight prevalence by wealth[wealth] = SUM( overweight adults[ adult age!, wealth])/SUM( adults sb[ adult age!, wealth]) |  |
| adult ow faltering change rate | Dmnl/Year | adult ow faltering change rate[adult age,wealth] = adult TP overweight faltering[ adult age, wealth]+RAMP( overweight ACR adults 2000[ adult age, wealth], INITIAL TIME, INITIAL TIME+5)+RAMP( overweight ACR adults 2005[ adult age, wealth], INITIAL TIME+5, INITIAL TIME+10)+RAMP( overweight ACR adults 2010[ adult age, wealth], INITIAL TIME+10, INITIAL TIME+15)+RAMP( overweight ACR adults 2015[ adult age, wealth], INITIAL TIME+15, INITIAL TIME+20)+RAMP( overweight ACR adults 2020[ adult age, wealth], INITIAL TIME+20, INITIAL TIME+23) |  |
| adult ow recovery change rate | Dmnl/Year | adult ow recovery change rate[adult age,wealth] = adult TP overweight recovery[ adult age, wealth]+RAMP(- overweight ACR adults 2000[ adult age, wealth], INITIAL TIME, INITIAL TIME+5)+RAMP(- overweight ACR adults 2005[ adult age, wealth], INITIAL TIME+5, INITIAL TIME+10)+RAMP(- overweight ACR adults 2010[ adult age, wealth], INITIAL TIME+10, INITIAL TIME+15)+RAMP(- overweight ACR adults 2015[ adult age, wealth], INITIAL TIME+15, INITIAL TIME+20)+RAMP(- overweight ACR adults 2020[ adult age, wealth], INITIAL TIME+20, INITIAL TIME+23) |  |
| adult stunting faltering change rate | Dmnl/Year | adult stunting faltering change rate[adult age,wealth] = adult TP stunting faltering[ adult age, wealth]+RAMP( stunting ACR adults 2000[ adult age, wealth], INITIAL TIME, INITIAL TIME+5)+RAMP( stunting ACR adults 2005[ adult age, wealth], INITIAL TIME+5, INITIAL TIME+10)+RAMP( stunting ACR adults 2010[ adult age, wealth], INITIAL TIME+10, INITIAL TIME+15)+RAMP( stunting ACR adults 2015[ adult age, wealth], INITIAL TIME+15, INITIAL TIME+20)+RAMP( stunting ACR adults 2020[ adult age, wealth], INITIAL TIME+20, INITIAL TIME+23) |  |
| adult stunting prevalence by age | Dmnl | adult stunting prevalence by age[adult age] = SUM( short stature adults[ adult age, wealth!])/SUM( adults sb[ adult age, wealth!]) |  |
| adult stunting prevalence by wealth | Dmnl | adult stunting prevalence by wealth[wealth] = SUM( short stature adults[ adult age!, wealth])/SUM( adults sb[ adult age!, wealth]) |  |
| adult stunting recovery change rate | Dmnl/Year | adult stunting recovery change rate[adult age,wealth] = adult TP stunting recovery[ adult age, wealth]+RAMP(- stunting ACR adults 2000[ adult age, wealth], INITIAL TIME, INITIAL TIME+5)+RAMP(- stunting ACR adults 2005[ adult age, wealth], INITIAL TIME+5, INITIAL TIME+10)+RAMP(- stunting ACR adults 2010[ adult age, wealth], INITIAL TIME+10, INITIAL TIME+15)+RAMP(- stunting ACR adults 2015[ adult age, wealth], INITIAL TIME+15, INITIAL TIME+20)+RAMP(- stunting ACR adults 2020[ adult age, wealth], INITIAL TIME+20, INITIAL TIME+23) |  |
| adult TP overweight faltering | Dmnl/Year | adult TP overweight faltering[A15to24,rich] = baseline adult TP overweight faltering[A15to24]/ adult cohort duration[A15to24] |  |
| adult TP overweight recovery | Dmnl/Year | adult TP overweight recovery[A15to24,rich] = baseline adult TP overweight recovery[A15to24]/ adult cohort duration[A15to24] |  |
| adults | people | = SUM( adults sb[ adult age!, wealth!]) | only for validation purposes - compare with observed trends |
| annual F and V increase | soles/(Year*Year*people) | = IF THEN ELSE( Time>2010, annual F and V change rate,0) |  |
| average adult overweight prevalence | Dmnl | average adult overweight prevalence[adult age,wealth] = overweight adults[ adult age, wealth]/ adults sb[ adult age, wealth] |  |
| average adults per recipient hh | people/household | average adults per recipient hh[adult age,poor] = ( hh size Juntos participants- average children Juntos participants)/3 |  |
| average children per hh peru | people/household | = (SUM( fertility rate[ adult age!]* adult cohort duration[ adult age!])*SUM( adults sb[ adult age!, wealth!]* female percentage))* average hh size Peru/(SUM( total population size[ wealth!])) | Estimate average lifetime births per household by combining fertility rates with the time spent at each adult age to reflect lifetime childbearing, scale by the number of adult females across ages and wealth, and convert to a household figure by multiplying by Peru’s average household size and dividing by total population. |
| average children per recipient household by age group | people/household | average children per recipient household by age group[child age,poor] = average children Juntos participants/3 |  |
| average short stature prevalence | Dmnl | average short stature prevalence[adult age,wealth] = short stature adults[ adult age, wealth]/ adults sb[ adult age, wealth] |  |
| Average stunting prevalence | Dmnl | Average stunting prevalence[child age,wealth] = stunted children[ child age, wealth]/ children sb[ child age, wealth] |  |
| baseline juntos TB coverage adults | Dmnl | baseline juntos TB coverage adults[adult age,poor] = current TB recipients by wealth adults[ adult age,poor]/ adults sb[ adult age,poor]* policy switch TB cash |  |
| baseline juntos TB coverage children | Dmnl | baseline juntos TB coverage children[child age,poor] = current TB recipients by wealth children[ child age,poor]/ children sb[ child age,poor]* policy switch TB cash |  |
| baseline TPI recipients hh from 2023 | household | = IF THEN ELSE( Time>2023, baseline TPI recipients hh,0) |  |
| change in SSB consumption due to tax | Dmnl/Year | change in SSB consumption due to tax[wealth] = IF THEN ELSE( Time> policy initiation:AND: Time<= policy peak,RAMP(( SSB own price elasticity[ wealth]* SSB tax with effect+ UPF tax rate* cross price elasticity UPF on SSB demand-( F and V subsidy rate* cross price elasticity F and V on SSB demand))/( policy peak- policy initiation), policy initiation, policy peak),IF THEN ELSE( Time>= policy peak:AND: Time< tax fade out year,( SSB own price elasticity[ wealth]* SSB tax with effect+ UPF tax rate* cross price elasticity UPF on SSB demand-( F and V subsidy rate* cross price elasticity F and V on SSB demand))*(1-RAMP(1/( tax fade out year- policy peak), policy peak, tax fade out year)),0)) | Estimated change in SSB consumption due to taxes (both SSB and UPF taxes) using published data on price elasticity and a RAMP function calculating a gradual increase in effect of tax over the first two years, followed by an incline over next three years, assuming that adaptation time as well as fade out of effectiveness on consumer behaviour. |
| change in UPF consumption due to tax | Dmnl/Year | change in UPF consumption due to tax[wealth] = IF THEN ELSE( Time> policy initiation:AND: Time<= policy peak,RAMP(( UPF own price elasticity[ wealth]* UPF tax rate+ cross price elasticity SSB on UPF demand* SSB tax rate-( F and V subsidy rate* cross price elasticity F and V on UPF demand))/( policy peak- policy initiation), policy initiation, policy peak),IF THEN ELSE( Time>= policy peak:AND: Time< tax fade out year,( UPF own price elasticity[ wealth]* UPF tax rate+ cross price elasticity SSB on UPF demand* SSB tax rate-( F and V subsidy rate* cross price elasticity F and V on UPF demand))*(1-RAMP(1/( tax fade out year- policy peak), policy peak, tax fade out year)),0)) | Estimated change in UPF consumption due to taxes (both SSB and UPF taxes) using published data on price elasticity and a RAMP function calculating a gradual increase in effect of tax over the first two years, followed by an incline over next five years, assuming that adaptation time as well as fade out of effectiveness on consumer behaviour after 7 years in total |
| child mortality rate | Dmnl/Year | child mortality rate[child age] = Baseline child mortality rate[ child age]+RAMP( child mortality rate annual change[ child age], INITIAL TIME, INITIAL TIME+20) |  |
| child overweight prevalence by wealth | Dmnl | child overweight prevalence by wealth[wealth,child age] = overweight children[ child age, wealth]/ children sb[ child age, wealth] |  |
| child ow faltering change rate | Dmnl/Year | child ow faltering change rate[child age,wealth] = child TP overweight faltering[ child age, wealth]+RAMP( overweight ACR children 2000[ child age, wealth], INITIAL TIME, INITIAL TIME+5)+RAMP( overweight ACR children 2005[ child age, wealth], INITIAL TIME+5, INITIAL TIME+10)+RAMP( overweight ACR children 2010[ child age, wealth], INITIAL TIME+10, INITIAL TIME+15)+RAMP( overweight ACR children 2015[ child age, wealth], INITIAL TIME+15, INITIAL TIME+20)+RAMP( overweight ACR children 2020[ child age, wealth], INITIAL TIME+20, INITIAL TIME+23) |  |
| child ow recovery change rate | Dmnl/Year | child ow recovery change rate[child age,wealth] = child TP overweight recovery[ child age, wealth]+RAMP(- overweight ACR children 2000[ child age, wealth], INITIAL TIME, INITIAL TIME+5)+RAMP(- overweight ACR children 2005[ child age, wealth], INITIAL TIME+5, INITIAL TIME+10)+RAMP(- overweight ACR children 2010[ child age, wealth], INITIAL TIME+10, INITIAL TIME+15)+RAMP(- overweight ACR children 2015[ child age, wealth], INITIAL TIME+15, INITIAL TIME+20)+RAMP(- overweight ACR children 2020[ child age, wealth], INITIAL TIME+20, INITIAL TIME+23) |  |
| child stunting faltering change rate | Dmnl/Year | child stunting faltering change rate[child age,wealth] = child TP stunting faltering[ child age, wealth]+RAMP( stunting ACR children 2000[ child age, wealth], INITIAL TIME, INITIAL TIME+5)+RAMP( stunting ACR children 2005[ child age, wealth], INITIAL TIME+5, INITIAL TIME+10)+RAMP( stunting ACR children 2010[ child age, wealth], INITIAL TIME+10, INITIAL TIME+15)+RAMP( stunting ACR children 2015[ child age, wealth], INITIAL TIME+15, INITIAL TIME+20)+RAMP( stunting ACR children 2020[ child age, wealth], INITIAL TIME+20, INITIAL TIME+23) |  |
| child stunting prevalence by wealth | Dmnl | child stunting prevalence by wealth[wealth] = SUM( stunted children[ child age!, wealth])/SUM( children sb[ child age!, wealth]) |  |
| child stunting prevalence by wealth and age | Dmnl | child stunting prevalence by wealth and age[child age,wealth] = stunted children[ child age, wealth]/ children sb[ child age, wealth] |  |
| child stunting recovery change rate | Dmnl/Year | child stunting recovery change rate[child age,wealth] = child TP stunting recovery[ child age, wealth]+RAMP(- stunting ACR children 2000[ child age, wealth], INITIAL TIME, INITIAL TIME+5)+RAMP(- stunting ACR children 2005[ child age, wealth], INITIAL TIME+5, INITIAL TIME+10)+RAMP(- stunting ACR children 2010[ child age, wealth], INITIAL TIME+10, INITIAL TIME+15)+RAMP(- stunting ACR children 2015[ child age, wealth], INITIAL TIME+15, INITIAL TIME+20)+RAMP(- stunting ACR children 2020[ child age, wealth], INITIAL TIME+20, INITIAL TIME+23) |  |
| child TP overweight faltering | Dmnl/Year | child TP overweight faltering[A0to4,rich] = baseline child TP overweight faltering[A0to4]/ child cohort duration |  |
| child TP overweight recovery | Dmnl/Year | child TP overweight recovery[A0to4,rich] = baseline child TP overweight recovery[A0to4]/ child cohort duration |  |
| child TP stunting faltering | Dmnl/Year | child TP stunting faltering[A0to4,rich] = baseline child TP stunting faltering[A0to4]/ child cohort duration |  |
| child TP stunting recovery | Dmnl/Year | child TP stunting recovery[A0to4,rich] = baseline child TP stunting recovery[A0to4]/ child cohort duration |  |
| children | people | = SUM( children sb[ child age!, wealth!]) | only for validation purposes - compare with observed trends |
| current SSB excise tax from policy initiation | Dmnl | = IF THEN ELSE( Time>= policy initiation, current SSB excise tax,0) |  |
| current TB coverage adults by wealth | Dmnl | current TB coverage adults by wealth[wealth] = SUM( current TB recipients by wealth adults[ adult age!, wealth])/SUM( adults sb[ adult age!, wealth]) |  |
| current TB coverage children by wealth | Dmnl | current TB coverage children by wealth[wealth] = SUM( current TB recipients by wealth children[ child age!, wealth])/SUM( children sb[ child age!, wealth]) | Overall child TB coverage rate: total current TB child recipients (summed over all ages and wealth) ÷ total child stock (summed over all ages and wealth). |
| current TB recipients adults | people | current TB recipients adults[adult age] = baseline TB recipient hh* average adults per recipient hh[ adult age,poor] | calculate adult TB recipients as: baseline TB recipient households × average adults per recipient household for that age, using the current recipient-household composition (poor subscript) |
| current TB recipients by wealth adults | people | current TB recipients by wealth adults[adult age,rich] = IF THEN ELSE( current TB recipients adults[ adult age]>SUM( adults sb[ adult age, wealth!])- adults sb[ adult age,rich], current TB recipients adults[ adult age]-(SUM( adults sb[ adult age, wealth!])- adults sb[ adult age,rich]),0) |  |
| current TB recipients by wealth children | people | current TB recipients by wealth children[child age,rich] = IF THEN ELSE( current TB recipients children[ child age]>SUM( children sb[ child age, wealth!])- children sb[ child age,rich], current TB recipients children[ child age]-(SUM( children sb[ child age, wealth!])- children sb[ child age,rich]),0) |  |
| current TB recipients children | people | current TB recipients children[child age] = baseline TB recipient hh* average children per recipient household by age group[ child age,poor] | Current number of children receiving Basic Transfers calculated by multiplying number of recipient households with average children living in recipient households |
| current TPI coverage by wealth | Dmnl | current TPI coverage by wealth[wealth] = SUM( current TPI recipients by wealth children[ child age!, wealth])/SUM( children sb[ child age!, wealth]) | Overall child TPI coverage rate |
| current TPI recipients by wealth children | people | current TPI recipients by wealth children[child age,rich] = IF THEN ELSE( current TPI recipients children[ child age]>SUM( children sb[ child age, wealth!])- children sb[ child age,rich], current TPI recipients children[ child age]-(SUM( children sb[ child age, wealth!])- children sb[ child age,rich]),0) |  |
| current TPI recipients children | people | current TPI recipients children[child age] = baseline TPI recipients hh from 2023* average children per recipient household by age group[ child age,poor] |  |
| delta SSB | Dmnl | delta SSB[wealth] = IF THEN ELSE( Market size SSB Peru[ wealth]< market size SSB 2024:AND: Time> policy initiation,100*( Market size SSB Peru[ wealth]- market size SSB 2024)/ market size SSB 2024,0) | Percentage difference in per capita SSB purchases from baseline pre tax (2024) and under taxation scenario |
| delta UPF | Dmnl | delta UPF[wealth] = IF THEN ELSE( Market size UPF Peru[ wealth]< UPF market size 2024:AND: Time>2024,100*( Market size UPF Peru[ wealth]- UPF market size 2024)/ UPF market size 2024,0) | Percentage difference in per capita UPF purchases from baseline pre tax (2024) and under taxation scenario |
| F and V subsidy rate | Dmnl | = IF THEN ELSE( Time> policy initiation, total taxation revenue/ total F and V market size/ allocation time* policy switch F and V subsidies,0) | This represents the fraction of the F&V market that can be subsidized each period given available revenue. |
| fertility rate | Dmnl/Year | fertility rate[adult age] = Baseline fertility rate[ adult age]+RAMP( fertility rate annual change[ adult age], INITIAL TIME, INITIAL TIME+20) | Assumption: Fertility rate changes between 2000 and 2020, according to observed data. After 2020, it does not change. |
| increase cash transfer TB | Dmnl | = new TB cash transferred per hh/ baseline TB cash transferred per hh*100 | Percentage increase in cash transfere by dividing possible new amount transferred by baseline transfer amount |
| LBW rate | Dmnl | LBW rate[adult age,wealth] = ( baseline LBW[ wealth]* average short stature prevalence[ adult age, wealth]* maternal height and LBW RR) |  |
| mortality rate overweight | 1/Year | mortality rate overweight[adult age] = adult mortality rate[ adult age]*( RR obesity mortality-1) |  |
| mortality rate stunting | 1/Year | mortality rate stunting[child age] = child mortality rate[ child age]*( RR stunting mortality-1) |  |
| new coverage TB adults | Dmnl | new coverage TB adults[adult age,wealth] = new TB recipient adults[ adult age, wealth]/ adults sb[ adult age, wealth] | Fraction of adults (by wealth and age) that newly starts receiving TB transfers. |
| new coverage TB adults by wealth | Dmnl | new coverage TB adults by wealth[wealth] = SUM( new TB recipient adults[ adult age!, wealth])/SUM( adults sb[ adult age!, wealth]) | Overall share of adults newly receiving TB across all ages and wealth groups: total new adult recipients ÷ total adult stock (summed over age×wealth). |
| new coverage TB children | Dmnl | new coverage TB children[child age,wealth] = new TB recipient children[ child age, wealth]/ children sb[ child age, wealth] | Incremental child coverage by age×wealth: new recipients ÷ child stock. |
| new coverage TB children by wealth | Dmnl | new coverage TB children by wealth[wealth] = SUM( new TB recipient children[ child age!, wealth])/SUM( children sb[ child age!, wealth]) | new number of recipients divided by total amount of children to obtain coverage rate |
| new coverage TPI | Dmnl | new coverage TPI[child age,wealth] = new TPI recipient ppl[ child age, wealth]/ children sb[ child age, wealth] |  |
| new TB cash transferred per hh | soles/(Year*household) | = IF THEN ELSE( Time> policy initiation, total taxation revenue/ baseline TB recipient hh* policy switch TB cash/ allocation time,0) |  |
| new TB recipient adults | people | new TB recipient adults[adult age,poor] = IF THEN ELSE( Time> policy initiation:AND: current TB recipients by wealth adults[ adult age,poor]+ total taxation revenue/ baseline TB cash transferred per hh/ allocation time* average adults per recipient hh[ adult age,poor]< adults sb[ adult age,poor]:AND: total coverage tb children[poor]<1, total taxation revenue/ baseline TB cash transferred per hh/ allocation time* average adults per recipient hh[ adult age,poor]* policy switch TB coverage,IF THEN ELSE( Time> policy initiation:AND: current TB recipients by wealth adults[ adult age,poor]+ total taxation revenue/ baseline TB cash transferred per hh/ allocation time* average adults per recipient hh[ adult age,poor]> adults sb[ adult age,poor]:AND: total coverage tb children[poor]<1,( adults sb[ adult age,poor]- current TB recipients by wealth adults[ adult age,poor])* policy switch TB coverage,0)) |  |
| new TB recipient children | people | new TB recipient children[child age,poor] = IF THEN ELSE( Time> policy initiation:AND: current TB recipients by wealth children[ child age,poor]< children sb[ child age,poor], total taxation revenue/ baseline TB cash transferred per hh/ allocation time* average children per recipient household by age group[ child age,poor]* policy switch TB coverage,0) |  |
| non overweight adults | people | non overweight adults[adult age,wealth] = adults sb[ adult age, wealth]- overweight adults[ adult age, wealth] |  |
| non overweight children | people | non overweight children[child age,wealth] = children sb[ child age, wealth]- overweight children[ child age, wealth] |  |
| non stunted adults | people | non stunted adults[adult age,wealth] = (1- average short stature prevalence[ adult age, wealth])* adults sb[ adult age, wealth] |  |
| non stunted children | people | non stunted children[child age,wealth] = children sb[ child age, wealth]- stunted children[ child age, wealth] |  |
| obese adults | people | obese adults[adult age,wealth] = 0.37* overweight adults[ adult age, wealth] | 0.37 is the proportion of obese adults among those who are overweight (data from World Food Programme) |
| overweight change rate cash juntos children | 1/Year | overweight change rate cash juntos children[child age,wealth] = EE Juntos cash on overweight* increase cash transfer TB* cash devaluation since start TB* baseline juntos TB coverage children[ child age, wealth]* policy switch TB cash |  |
| overweight change rate coverage juntos children | Dmnl/Year | overweight change rate coverage juntos children[child age,wealth] = (( EE Juntos coverage on overweight children* new coverage TB children[ child age, wealth])-((1- cash devaluation since start TB)* EE Juntos cash on overweight))* policy switch TB coverage |  |
| overweight change rate F and V subsidies | Dmnl/Year | overweight change rate F and V subsidies[wealth] = IF THEN ELSE( Time> policy initiation:AND: Time<= policy peak,RAMP( F and V subsidy rate* EE F and V prices on overweight* proportion benefitting from F and V subsidies[ wealth]/( policy peak- policy initiation), policy initiation, policy peak),IF THEN ELSE( Time>= policy peak:AND: Time< subsidy fade out year,( F and V subsidy rate* EE F and V prices on overweight* proportion benefitting from F and V subsidies[ wealth])*(1-RAMP(1/( subsidy fade out year- policy peak), policy peak, subsidy fade out year)),0)) |  |
| policy initiation | Year | = INITIAL TIME+24 | Assumes policy initiation in Year 2024 |
| policy peak | Year | = policy initiation+ policy peak time |  |
| revenue SSB taxation | soles/Year | revenue SSB taxation[wealth] = Market size SSB Peru[ wealth]* SSB tax rate* total population size[ wealth]* proportion taxable drinks | Revenue calculation from SSB tax multiplying market size per capita with population size (both stratified by wealth) and proportion of taxable drinks (only those with >6mg of sugar/100ml) |
| SSB annual increase | soles/(people*Year*Year) | SSB annual increase[wealth] = ( SSB goal market size- Market size SSB Peru[ wealth])/ time to close SSB market size gap | Estimated annual increase in Market size of SSB sales |
| SSB effect on overweight | Dmnl/Year | SSB effect on overweight[wealth] = delta SSB[ wealth]* EE SSB consumption on overweight* policy switch SSB tax |  |
| SSB tax rate | Dmnl | = current SSB excise tax*(1- policy switch SSB tax)+ Scenario SSB tax* policy switch SSB tax | This is the SSB tax rate. When the policy switch for SSB tax is 0, then the SSB tax rate is equal to the current SSB tax rate (i.e. 25%). When the policy switch for SSB tax is 1, then the SSB taz rate is equal to the Scenario SSB tax (currently set at 30%) |
| SSB tax with effect | Dmnl | = ( SSB tax rate- current SSB excise tax from policy initiation)* policy switch SSB tax | This is the SSB tax whose effect is modelled.When the SSB tax rate is equal to the current SSB excise tax (i.e. 25%), then no effect from the tax is modelled, as we assume that the current tax's effect is already considered under the status quo scenario.When the SSB tax rate is higher than the current SSB excise tax, then we only model the effect of the tax that is above the current excise tax. |
| stunting change rate cash juntos | Dmnl/Year | stunting change rate cash juntos[child age,wealth] = EE Juntos cash on stunting* increase cash transfer TB* cash devaluation since start TB* baseline juntos TB coverage children[ child age, wealth]* policy switch TB cash |  |
| stunting change rate coverage juntos | Dmnl/Year | stunting change rate coverage juntos[child age,wealth] = (( EE Juntos coverage on stunting* new coverage TB children[ child age, wealth])-((1- cash devaluation since start TB)* EE Juntos cash on stunting))* policy switch TB coverage |  |
| stunting change rate subsidies | Dmnl/Year | stunting change rate subsidies[wealth] = IF THEN ELSE( Time> policy initiation:AND: Time<= policy peak,RAMP( F and V subsidy rate* EE F and V prices on stunting* proportion benefitting from F and V subsidies[ wealth]/( policy peak- policy initiation), policy initiation, policy peak),IF THEN ELSE( Time>= policy peak:AND: Time< subsidy fade out year,( F and V subsidy rate* EE F and V prices on stunting* proportion benefitting from F and V subsidies[ wealth])*(1-RAMP(1/( subsidy fade out year- policy peak), policy peak, subsidy fade out year)),0)) |  |
| stunting change rate TPI expansion | 1/Year | stunting change rate TPI expansion[child age,wealth] = (( EE Juntos TPI expansion* new coverage TPI[ child age, wealth])-((1- cash devaluation since start TPI)* EE Juntos cash on stunting)* policy switch TPI coverage) |  |
| subsidy fade out year | Year | = policy initiation+ subsidy fade out time |  |
| tax fade out year | Year | = policy initiation+ tax fade out time |  |
| total adult overweight prevalence | Dmnl | = SUM( overweight adults[ adult age!, wealth!])/SUM( adults sb[ adult age!, wealth!]) |  |
| total child stunting prevalence | Dmnl | = SUM( stunted children[ child age!, wealth!])/SUM( children sb[ child age!, wealth!]) |  |
| total coverage tb children | Dmnl | total coverage tb children[wealth] = current TB coverage children by wealth[ wealth]+ new coverage TB children by wealth[ wealth] | overall coverage of TB for children summing up current coverage and new coverage. |
| total coverage TPI | Dmnl | total coverage TPI[child age,wealth] = current TPI coverage by wealth[ wealth]+ new coverage TPI[ child age, wealth] | By age and wealth, total TPI coverage = existing wealth-level coverage (current TPI coverage by wealth, defined only for ages 0–4 so no age subscript) + this period’s increment for that specific age and wealth group (new coverage TPI) |
| total F and V market size | soles/Year | = F and V market size*SUM( total population size[ wealth!]) | Market size calculated using per capita market size multiplied with population size (by wealth) |
| total overweight change rate adults | 1/Year | total overweight change rate adults[adult age,wealth] = IF THEN ELSE( Time> policy initiation, SSB effect on overweight[ wealth]+ UPF effect on overweight[ wealth]+ overweight change rate F and V subsidies[ wealth],0) |  |
| total overweight change rate children | 1/Year | total overweight change rate children[child age,wealth] = IF THEN ELSE( Time> policy initiation, overweight change rate cash juntos children[ child age, wealth]+ overweight change rate coverage juntos children[ child age, wealth]+ SSB effect on overweight[ wealth]+ UPF effect on overweight[ wealth]+ overweight change rate F and V subsidies[ wealth],0) |  |
| total population size | people | total population size[wealth] = SUM( children sb[ child age!, wealth])+SUM( adults sb[ adult age!, wealth]) |  |
| total stunting change rate children | 1/Year | total stunting change rate children[child age,wealth] = IF THEN ELSE( Time> policy initiation, stunting change rate coverage juntos[ child age, wealth]+ stunting change rate cash juntos[ child age, wealth]+ stunting change rate TPI expansion[ child age, wealth]+ stunting change rate subsidies[ wealth],0) |  |
| under five overweight prevalence | Dmnl | = SUM( overweight children[A0to4, wealth!])/SUM( children sb[A0to4, wealth!]) |  |
| under five stunting prevalence | Dmnl | = SUM( stunted children[A0to4, wealth!])/SUM( children sb[A0to4, wealth!]) |  |
| UPF annual increase | soles/(people*Year*Year) | UPF annual increase[wealth] = ( UPF goal market size- Market size UPF Peru[ wealth])/ time to close UPF market size gap | Estimated annual increase in Market size of UPF sales |
| UPF effect on overweight | Dmnl/Year | UPF effect on overweight[wealth] = delta UPF[ wealth]* EE UPF consumption on overweight* policy switch UPF tax |  |
| UPF tax rate | Dmnl | = IF THEN ELSE( Time>= policy initiation, UPF tax* policy switch UPF tax,0) | For time past policy initiation (2024), UPF tax multiplied with policy switch (0 or 1) depending on if UPF tax is modelled or not. |
| UPF taxation revenue | soles/Year | UPF taxation revenue[wealth] = Market size UPF Peru[ wealth]* UPF tax rate* total population size[ wealth]* policy switch UPF tax | Revenue calculation from UPF tax multiplying market size per capita with population size (both stratified by wealth) |
| SAVEPER | Year | = TIME STEP | The frequency with which output is stored. |
| *Stocks* | | | |
| adults sb | people | adults sb[A15to24,wealth] = ∫( child aging[A10to14, wealth]- adult aging[A15to24, wealth])- adult death rate[A15to24, wealth] dt + baseline population adults[A15to24]* proportion in lowest and upper wealth quartile adults[ wealth] |  |
| children sb | people | children sb[A0to4,wealth] = ∫( births[ wealth]- child aging[A0to4, wealth])- child death rate[A0to4, wealth] dt + baseline population children[A0to4]* proportion in lowest and upper wealth quartile children[ wealth] |  |
| cumulative person years of overweight adults | Year*people | cumulative person years of overweight adults[adult age,wealth] = ∫ overweight adults per year[ adult age, wealth] dt + 0.0 |  |
| cumulative person years of overweight adults by wealth | Year*people | cumulative person years of overweight adults by wealth[wealth] = ∫ overweight adults per year by wealth[ wealth] dt + 0.0 |  |
| cumulative person years of stunted children | Year*people | cumulative person years of stunted children[child age,wealth] = ∫ stunted children per year[ child age, wealth] dt + 0.0 |  |
| cumulative person years of stunted children by wealth |  | cumulative person years of stunted children by wealth[wealth] = ∫ stunted children pe year by wealth[ wealth] dt + 0.0 |  |
| F and V market size | soles/(Year*people) | = ∫ F and V market size change rate dt + F and V market size 2010 |  |
| Market size SSB Peru | soles/(people*Year) | Market size SSB Peru[wealth] = ∫ SSB sales change rate[ wealth] dt + Market size SSB Peru 2000 | Stock representing SSB market size in Peru, using goal seeking loop for Peruvian SSB market size to to reach Latin American levels of 2024 by 2040. |
| Market size UPF Peru | soles/(people*Year) | Market size UPF Peru[wealth] = ∫ UPF sales change rate[ wealth] dt + UPF market size 2000 | Stock representing UPF market size in Peru, using goal seeking loop for Peruvian UPF market size to to reach Latin American levels of 2024 by 2040. |
| overweight adults | people | overweight adults[A15to24,wealth] = ∫(((( overweight aging[A10to14, wealth]- adult overweight aging[A15to24, wealth])+ adult overweight faltering[A15to24, wealth])- adult overweight recovery[A15to24, wealth])- adult overweight deaths[A15to24, wealth])+ overweight flow due to policy adults[A15to24, wealth] dt + Baseline adult overweight prevalence[A15to24, wealth]* adults sb[A15to24, wealth] |  |
| overweight children | people | overweight children[A0to4,wealth] = ∫((( overweight births[ wealth]- overweight aging[A0to4, wealth])+ child overweight faltering[A0to4, wealth])- child overweight recovery[A0to4, wealth])+ overweigt flow due to policy children[A0to4, wealth] dt + baseline child overweight prevalence[A0to4, wealth]* children sb[A0to4, wealth] |  |
| short stature adults | people | short stature adults[A15to24,wealth] = ∫(( stunted aging[A10to14, wealth]- short stature aging[A15to24, wealth])- adult stunting recovery[A15to24, wealth])+ adult stunting faltering[A15to24, wealth] dt + baseline short stature prevalence[A15to24, wealth]* adults sb[A15to24, wealth] |  |
| stunted children | people | stunted children[A0to4,wealth] = ∫(((((SUM( stunted births[ adult age!, wealth]))- stunted aging[A0to4, wealth])- child stunting recovery[A0to4, wealth])+ child stunting faltering[A0to4, wealth])- child stunting deaths[A0to4])+ stunting change children due to policy[A0to4, wealth] dt + baseline child stunting prevalence[A0to4, wealth]* children sb[A0to4, wealth] |  |
| total taxation revenue | soles | = ∫ tax inflow- spending outflow dt + 1.0 |  |
| *Flow* | | | |
| adult aging | people/Year | adult aging[adult age,wealth] = adults sb[ adult age, wealth]/ adult cohort duration[ adult age] |  |
| adult death rate | people/Year | adult death rate[adult age,wealth] = ( adults sb[ adult age, wealth]* adult mortality rate[ adult age]) |  |
| adult overweight aging | people/Year | adult overweight aging[adult age,wealth] = overweight adults[ adult age, wealth]/ adults sb[ adult age, wealth]* adult aging[ adult age, wealth] |  |
| adult overweight deaths | people/Year | adult overweight deaths[adult age,wealth] = obese adults[ adult age, wealth]* mortality rate overweight[ adult age] |  |
| adult overweight faltering | people/Year | adult overweight faltering[A15to24,wealth] = 0 |  |
| adult overweight recovery | people/Year | adult overweight recovery[A15to24,wealth] = 0 |  |
| births | people/Year | births[wealth] = SUM( adults sb[ adult age!, wealth]* fertility rate[ adult age!])* female percentage |  |
| child aging | people/Year | child aging[child age,wealth] = IF THEN ELSE( children sb[ child age, wealth]<=0,0, children sb[ child age, wealth]/ child cohort duration) |  |
| child death rate | people/Year | child death rate[child age,wealth] = IF THEN ELSE( children sb[ child age, wealth]<=0,0, children sb[ child age, wealth]* child mortality rate[ child age]) |  |
| F and V market size change rate | soles/(people*Year*Year) | = annual F and V increase |  |
| overweight adults per year | people | overweight adults per year[adult age,wealth] = IF THEN ELSE( Time> policy initiation, overweight adults[ adult age, wealth],0) |  |
| overweight adults per year by wealth | people | overweight adults per year by wealth[wealth] = IF THEN ELSE( Time< policy initiation,0,SUM( overweight adults[ adult age!, wealth])) |  |
| overweight aging | people/Year | overweight aging[child age,wealth] = overweight children[ child age, wealth]/ children sb[ child age, wealth]* child aging[ child age, wealth] |  |
| overweight births | people/Year | overweight births[wealth] = baseline infant overweight prevalence[ wealth]* births[ wealth] |  |
| overweight flow due to policy adults | people/Year | overweight flow due to policy adults[adult age,wealth] = total overweight change rate adults[ adult age, wealth]* overweight adults[ adult age, wealth] |  |
| overweight flow due to policy children | people/Year | overweigt flow due to policy children[child age,wealth] = overweight children[ child age, wealth]* total overweight change rate children[ child age, wealth] |  |
| short stature aging | people/Year | short stature aging[adult age,wealth] = average short stature prevalence[ adult age, wealth]* adult aging[ adult age, wealth] |  |
| spending outflow | soles/Year | = IF THEN ELSE( Time> policy initiation,SUM( new TB recipient children[ child age!, wealth!])/SUM( average children per recipient household by age group[ child age!, wealth!])* baseline TB cash transferred per hh* policy switch TB coverage+SUM( new TB recipient adults[ adult age!, wealth!])/SUM( average adults per recipient hh[ adult age!, wealth!])* baseline TB cash transferred per hh* policy switch TB coverage+ baseline TB recipient hh* new TB cash transferred per hh* policy switch TB cash+SUM( new TPI recipient ppl[ child age!, wealth!])/SUM( average children per recipient household by age group[A0to4, wealth!])* baseline TPI costs per hh* policy switch TPI coverage+ F and V subsidy rate* total F and V market size* policy switch F and V subsidies,0) | After policy initiation, total spending equals the sum of scenario-specific spending, each line is a different policy scenario and is multiplied by its policy switch (0/1) to compute the spending outcome under that scenario (before policy start: 0).TBcoverage expansion (children): Convert new child recipients to recipient households (new TB recipient children ÷ average children per recipient HH across age×wealth) and pay baseline TB cash per HH.TB coverage expansion (adults): Convert new adult recipients to recipient households (new TB recipient adults ÷ average adults per recipient HH across age×wealth) and pay baseline TB cash per HH.TB cash top-up for existing recipients: Pay the incremental transfer (new TB cash transferred per hh) to all baseline TB recipient households.TPI coverage expansion: Convert new TPI recipients (people) to recipient households using the A0–4 household conversion, then pay baseline TPI cost per HH.Fruit & Veg subsidies: Pay subsidy rate × total F&V market size. |
| SSB sales change rate | soles/(people*Year*Year) | SSB sales change rate[wealth] = SSB annual increase[ wealth]+( change in SSB consumption due to tax[ wealth]* Market size SSB Peru[ wealth]) |  |
| stunted aging | people/Year | stunted aging[child age,wealth] = Average stunting prevalence[ child age, wealth]* child aging[ child age, wealth] |  |
| stunted births | people/Year | stunted births[adult age,wealth] = baseline infant stunting[ wealth]* births[ wealth]*((1- LBW rate[ adult age, wealth])+( LBW rate[ adult age, wealth]* LBW and stunting RR)) |  |
| stunted children pe year by wealth | people | stunted children pe year by wealth[wealth] = IF THEN ELSE( Time< policy initiation,0,SUM( stunted children[ child age!, wealth])) |  |
| stunted children per year | people | stunted children per year[child age,wealth] = IF THEN ELSE( Time> policy initiation, stunted children[ child age, wealth],0) |  |
| stunting change children due to policy | people/Year | stunting change children due to policy[child age,wealth] = stunted children[ child age, wealth]* total stunting change rate children[ child age, wealth] |  |
| tax inflow | soles/Year | = IF THEN ELSE( Time>= policy initiation,SUM( revenue SSB taxation[ wealth!])+SUM( UPF taxation revenue[ wealth!]),0) | Sum of tax revenue inflow |
| UPF sales change rate | soles/(people*Year*Year) | UPF sales change rate[wealth] = UPF annual increase[ wealth]+( Market size UPF Peru[ wealth]* change in UPF consumption due to tax[ wealth]) |  |

Dmnl = dimensionless. ow: overweight. TPI = *Early Childhood Transfer* of Juntos. F and V = fruit and vegetables. hh = household. TB = *Basic Transfer* of Juntos. UPF = ultra-processed food. TP: Transition probability. . SSB = sugar-sweetened beverage. LBW = Low birth weight.
