# Supplementary material for: Retrofitting fiscal policies to reduce the double burden of malnutrition in Peru: A system dynamics modelling study
Source: PLoS Med. 2026 Aug 13;23(8):e1005167. doi: 10.1371/journal.pmed.1005167 (PMC13472416; doi:10.1371/journal.pmed.1005167)
Supplement: Supplementary file 9 [file pmed.1005167.s009.docx]

S5 Text. Sensitivity testing

We performed several sensitivity analyses, using Vensim’s Sensitivity2All tool to assess the model's response to parameter changes. Specifically, we measured the effects of ±10% variations in exogenous parameters on average adult overweight prevalence and under-5 stunting prevalence. Parameters showing the highest sensitivity impact on these variables were further examined through numerical and behavioral sensitivity tests. Numerical sensitivity focused on the percentage change in these variables, while behavioral sensitivity analysed changes in trends over time. Sensitivity testing was conducted separately, once for the population model only (Figure 1 and Figure 3) and once for the model with all policy scenarios switched on (Figure 2 and Figure 4).

**Figure 1.** Tornado graphs showing numerical sensitivity for the population model.

Graph illustrates the constant variables, their varying values (representing +/-10%), and their respective impacts on: **(A)** average adult overweight prevalence; and **(B)** under-five stunting prevalence. The 15 most influential constant variables affecting each outcome are shown.

TP: Transition Probability. RR: Relative Risk. ACR = Annual Change Rate

**Figure 2.** Tornado graphs showing numerical sensitivity for the policy model.

The graph illustrates the constant variables, their varying values (representing +/-10%), and their respective impacts on: **(A)** average adult overweight prevalence; and **(B)** under-five stunting prevalence. The 15 most influential constant variables affecting each outcome are shown.

Dmnl = dimensionless. EE: Effect estimate. SSB = sugar-sweetened beverage. UPF = ultra-processed food.

**Figure 3.** Behaviour-over-time graph showing behavioural sensitivity for population model.

Graphs illustrate the sensitivity of **(A)** average overweight prevalence and **(B)** under five stunting prevalence to varying parameter values using the population model (without policies). The most influential constant variables identified in numerical sensitivity analysis are adjusted by -10% (red line) or +10% (blue line) compared to the baseline scenario (black line).

**(A)**

**(B)**

Dmnl = dimensionless. TP: Transition probability. RR: Relative Risk. ACR = Annual Change Rate

**Figure 4.** Behaviour-over-time graph showing behavioural sensitivity of policy model.

Graphs illustrate sensitivity of **(A)** average overweight prevalence and **(B)** under five stunting prevalence to varying parameter values using the policy model (30% SSB tax, 10% UPF tax and all policies activated). The most influential constant variables identified in numerical sensitivity analysis are adjusted by -10% (red line) or +10% (blue line) compared to the baseline scenario (black line).

**(B)**

UPF = ultra-processed food. SSB = sugar-sweetened beverage. TP = Transition Probability. OR: Odds Ratio. RR: Relative Risk

**Conclusions:**

Sensitivity analyses identified that the population-model variables with the largest numerical impact on adult overweight and under-5 stunting prevalence were baseline adult mortality rates, definition of age groups (i.e. cohort duration), baseline overweight and stunting prevalence, and transition probabilities (e.g., recovery and faltering rates and their annual changes). However, changes in all these variables by ±10% resulted in small deviations (<5%) on overweight and under-5 stunting prevalence.

Sensitivity analyses found overweight prevalence most affected by parameters related to UPF and SSB market size projections, including the magnitude and timeline of projected growth, as well as tax-related factors such as tax rates and tax fade-out time. While overweight prevalence displayed notable numerical sensitivity (>5%) to ±10% changes in some of these parameters, behavioural sensitivity analysis showed that overall behaviour-over-time remained consistent upon parameter changes. In contrast, stunting prevalence was minimally impacted by these policy-related parameters. Even with all policies activated, stunting prevalence was predominantly influenced by population-model parameters. For these parameters, numerical sensitivity was minor (<5%), and behaviour-over-time graphs displayed consistent trends.
