# Supplementary material for: Retrofitting fiscal policies to reduce the double burden of malnutrition in Peru: A system dynamics modelling study
Source: PLoS Med. 2026 Aug 13;23(8):e1005167. doi: 10.1371/journal.pmed.1005167 (PMC13472416; doi:10.1371/journal.pmed.1005167)
Supplement: Supplementary file 10 [file pmed.1005167.s010.docx]

**S6 Text. Extreme condition testing**

We assessed how the model behaved under extreme values, parameters, and policy scenarios. This test focused on variables most affected by significant assumptions, especially those impacting adult overweight and under-5 stunting, as identified through parameter assessment. Extreme value testing was conducted separately for the population model only (Table 1) and once for the policy model (Table 2). For the policy model, The variables tested were those influenced by critical assumptions, such as "UPF goal market size," "SSB goal market size," "time to close the SSB market size gap," "time to close the UPF market size gap," "tax fade-out time," "subsidy fade-out time," "SSB tax," and "UPF tax".

**Table 1.** Behaviour-over-time graphs for testing extreme conditions for key parameters of the population model.

| **Variables** | **Description** | **Simulation results** |
| --- | --- | --- |
| Mortality rates | Mortality rates at baseline and their changes over time for all age groups were obtained from the Instituto Nacional de Estadística e Informática (INEI) of Peru. Mortality rates are modelled based on the baseline rate (year 2000) and annual change towards 2020. In the minimum extreme scenario, all variables are assumed to be zero (no deaths). In the maximum extreme scenario, baseline mortality is set to 1, with no change during the simulation. These scenarios assume either no population reduction (min mortality) or rapid reduction to zero (max mortality). This results in similar effects on overall overweight and stunted populations, as shown in the bottom graphs, though prevalence remains unchanged since it reflects population ratios. The behaviour over time aligns with expectations under these extremes. |        |
| Fertility rates | Fertility rates at baseline and their changes over time for all age groups were obtained from the Instituto Nacional de Estadística e Informática (INEI) of Peru. Rates are modelled based on the baseline rate (year 2000) and annual changes through 2020. In the minimum extreme scenario, fertility and annual change are set to zero (no births). In the maximum extreme scenario, fertility is set to 1, meaning every reproductive-age female gives birth yearly, with no change during the simulation. These scenarios result in either no population growth and eventual decline due to mortality and aging effects (min fertility) or extremely rapid expansion (max fertility). A separate graph is shown for the minimum scenario due to scale differences. The behaviour over time aligns with expectations under these extremes. |        |

**Table 2.** Behaviour-over-time graphs for testing extreme conditions for key parameters of the policy model.

| **Variables** | **Description** | **Simulation results** |
| --- | --- | --- |
| SSB goal market size,  UPF goal market size | In the simulation, UPF and SSB market sizes start at baseline levels from 2000 and grow to reach the average Latin American market size by 2040 (goal market size), reproducing observed trends up to 2020. The status quo (policies on) serves as the baseline scenario, with all policies active and parameters set as defined in the model. To test extremes, we simulated two scenarios: market stagnation after 2024 when taxes are implemented (minimum scenario) and market growth doubling the Latin American average by 2040 (maximum scenario). Over time, the tax effect is offset by SSB market growth; if growth stagnates, overweight decreases, while doubling growth nullifies the tax effect. For stunting, greater market growth generates more tax revenue, enabling stronger stunting reductions, with the maximum scenario showing the greatest impact. These simulations align with expected outcomes. |    |
| Time to close SSB market size gap, time to close UPF market size gap | In the simulation, UPF and SSB markets reach average Latin American levels by 2040 (40 years after the model's start). The status quo (policies on) serves as the baseline scenario, with all policies active and parameters set as defined in the model. To test extremes, we simulated two scenarios: markets reaching Latin American levels in 20 years (by 2020, minimum scenario) or in 80 years (maximum scenario), with the latter representing half the growth rate and extending beyond the model's boundaries. Over time, the tax effect is offset by market growth; slower growth (80 years) reduces overweight prevalence, while faster growth (20 years) nullifies the tax effect. For stunting, faster market growth generates more tax revenue, enabling stronger reductions, while slower growth results in less revenue and weaker reductions. These results align with expectations. |    |
| UPF and SSB taxes | In the simulation, we apply a 30% SSB tax and a 10% UPF tax. The status quo (policies on) serves as the baseline scenario, with all policies active and parameters as defined in the model. The minimum tax condition represents the status quo (policies off) with no additional taxes. Extreme scenarios involve setting UPF and SSB taxes to 100%, both individually and jointly. The combined effect of 100% taxes on both is less than the sum of their separate effects, as the pool of overweight individuals shrinks, reducing the impact of additional taxes. For stunting, higher taxes generate more funds for policies, resulting in greater stunting reductions. These simulations align with expectations. |    |
| Tax fade out time | In the simulation, we model a 7-year tax fade-out based on the literature. The status quo (policies on) serves as the baseline scenario, with all policies active and parameters as defined in the model. For extreme conditions, we simulate a 1-year tax fade-out (minimum) and a fade-out extending until 2040 (16 years, maximum). Overweight prevalence decreases far less when the tax effect fades after 1 year, while a sustained fade-out over time results in a more significant reduction. Stunting is only slightly impacted, with the 1-year fade-out yielding marginally greater reductions due to higher market growth and increased purchases, generating more funds for stunting policies. These outcomes align with expectations. |    |
| Subsidy fade out time | In the simulation, we model a 7-year subsidy fade-out. The status quo (policies on) serves as the baseline scenario, with all policies active and parameters as defined in the model. For extreme conditions, we simulate a 1-year subsidy fade-out (minimum) and a fade-out extending until 2040 (16 years, maximum). Overweight prevalence decreases slightly less when the subsidy effect fades after 1 year, while a sustained fade-out over time results in a larger reduction. Same applies for stunting, with slightly larger magnitudes. These outcomes align with expectations. |    |

Dmnl = dimensionless. 1 = dimensionless
