# Supplementary material for: Retrofitting fiscal policies to reduce the double burden of malnutrition in Peru: A system dynamics modelling study
Source: PLoS Med. 2026 Aug 13;23(8):e1005167. doi: 10.1371/journal.pmed.1005167 (PMC13472416; doi:10.1371/journal.pmed.1005167)
Supplement: Supplementary file 11 [file pmed.1005167.s011.docx]

**S7 Text. Comparison of integration methods and time steps**

System Dynamics models are systems of simultaneous differential equations that are solved algorithmically by numerical methods such as the Euler or Runge-Kutta fourth-order method.[1] When running these models, the time step (known as DT) in which the differential equations are solved must be defined. This test evaluates whether the model results are sensitive to changes in the integration method and the DT value.[1] We ran the model multiple times using different time steps for two integration methods: Euler and Runge-Kutta fourth-order. Finally, we compared the results of the two integration methods against each other.[1] This test was only performed for the population model.

**Table 1.** Model testing for the Euler integration method.

Adult overweight prevalence and under-five stunting prevalence under varying time step values (DT) from 1 to 0.0078125, across a total of 8 simulations using the Euler integration method. Difference shown in the table is calculated at the simulation midpoint (2020) and endpoint (2040) relative to the smallest DT.

| **Simulation results** | **Observation** |
| --- | --- |
| **Average adult overweight prevalence all ages** \| \|; **Integration type; Time step (DT)** \| **% Difference (compared to smallest DT value)** **t = 2020** \| **% Difference (compared to smallest DT value)** **t = 2040**; Euler; DT 0. 0078125 \| 0.000% \| 0.000%; Euler; DT 0.015625 \| -0.005% \| 0.003%; Euler; DT 0.03125 \| -0.014% \| 0.010%; Euler; DT 0.0625 \| -0.032% \| 0.022%; Euler; DT 0.125 \| -0.069% \| 0.048%; Euler; DT 0.25 \| -0.142% \| 0.100%; Euler; DT 0.5 \| -0.288% \| 0.207%; Euler; DT 1 \| -0.569% \| 0.435%  | The S-shape of the curve becomes slightly more pronounced as DT increases. For all DT values, the % difference remains below 1% at both the simulation midpoint (2020) and endpoint (2040). |
| **Average under five stunting prevalence** \| \|; **Integration type; Time step (DT)** \| **% Difference (compared to smallest DT value)** **t = 2020** \| **% Difference (compared to smallest DT value)** **t = 2040**; Euler; DT 0.0078125 \| 0.000% \| 0.000%; Euler; DT 0.015625 \| -0.007% \| -0.001%; Euler; DT 0.03125 \| -0.020% \| -0.001%; Euler; DT 0.0625 \| -0.048% \| -0.004%; Euler; DT 0.125 \| -0.102% \| -0.009%; Euler; DT 0.25 \| -0.216% \| -0.019%; Euler; DT 0.5 \| -0.458% \| -0.036%; Euler; DT 1 \| -1.006% \| -0.064%  | The curve depth decreases slightly as DT increases, particularly at DT = 1. At the simulation midpoint (2020), the % difference exceeds 1% for DT = 1, suggesting lower DT values are necessary to accurately capture the dynamics. For all other DT values, the % difference remains below 1% at both the midpoint and endpoint. |

DT: delta time

**Table 2.** Model testing for the Runge-Kutta integration method.

Adult overweight prevalence and under-five stunting prevalence under varying time step values (DT) from 1 to 0.0078125, across a total of 8 simulations using the Runge-Kutta’s fourth order integration method. % Difference is calculated for the simulated values at the midpoint of the simulation (t = 2020) and at the end of the simulation (t = 2040) compared to the smallest DT.

| **Simulation results** | **Observations** |
| --- | --- |
| **Average adult overweight prevalence all ages** \| \|; **Integration type; Time step (DT)** \| **% Difference (compared to smallest DT value)** **t = 2020** \| **% Difference (compared to smallest DT value)** **t = 2040**; Runge-Kutta 4; DT 0.0078125 \| 0.000% \| 0.000%; Runge-Kutta 4; DT 0.015625 \| -0.011% \| -0.002%; Runge-Kutta 4; DT 0.03125 \| -0.034% \| -0.006%; Runge-Kutta 4; DT 0.0625 \| -0.080% \| -0.015%; Runge-Kutta 4; DT 0.125 \| -0.173% \| -0.032%; Runge-Kutta 4; DT 0.25 \| -0.358% \| -0.066%; Runge-Kutta 4; DT 0.5 \| -0.734% \| -0.136%; Runge-Kutta 4; DT 1 \| -1.504% \| -0.279%  | There is no change in behavioural patterns when the DT changes.  For all DT values the % difference is less than 0.01% both at the midpoint of the simulation (t = 2020) and at the end of the simulation (t = 2040). |
| **Average under five stunting prevalence** \| \|; **Integration type; Time step (DT)** \| **% Difference (compared to smallest DT value)** **t = 2020** \| **% Difference (compared to smallest DT value)** **t = 2040**; Runge-Kutta 4; DT 0.0078125 \| 0.000% \| 0.000%; Runge-Kutta 4; DT 0.015625 \| 0.012% \| 0.000%; Runge-Kutta 4; DT 0.03125 \| 0.035% \| 0.002%; Runge-Kutta 4; DT 0.0625 \| 0.082% \| 0.004%; Runge-Kutta 4; DT 0.125 \| 0.177% \| 0.008%; Runge-Kutta 4; DT 0.25 \| 0.375% \| 0.016%; Runge-Kutta 4; DT 0.5 \| 0.793% \| 0.035%; Runge-Kutta 4; DT 1 \| 1.706% \| 0.074%  | The deep of the curve is slightly higher as DT increases, especially for the largest DT = 1.  For all DT values the % difference is less than 0.2% both at the midpoint of the simulation (t = 2020) and at the end of the simulation (t = 2040). |

DT: delta time

**Table 3.** Comparison of results between Euler's method and Runge-Kutta 4 integration method. The table highlights differences in simulation outcomes for time steps (DT) values ranging from 1 to 0.0078125 (16 simulations). % Difference is calculated at the simulation midpoint (2020) and endpoint (2040) between the two methods for 8 DT values.

| **Simulation results** | **Observations** |
| --- | --- |
| **Average adult overweight prevalence all ages** \| \|; **Time step (DT)** \| **% Difference (Euler method compared to Runge-Kutta´s fourth-order method)** **t = 2020** \| **% Difference (Euler method compared to Runge-Kutta´s fourth-order method)** **t = 2040**; DT 0.0078125 \| 0.007% \| 0.005%; DT 0.015625 \| 0.014% \| 0.011%; DT 0.03125 \| 0.027% \| 0.021%; DT 0.0625 \| 0.055% \| 0.043%; DT 0.125 \| 0.111% \| 0.085%; DT 0.25 \| 0.223% \| 0.172%; DT 0.5 \| 0.457% \| 0.349%; DT 1 \| 0.956% \| 0.721% | For all DT values, the % difference between Euler and Runge-Kutta's fourth-order method remains below 1% at both the simulation midpoint (2020) and endpoint (2040). However, differences increase with larger DTs, reinforcing the need for lower DT values. |
| **Average under five stunting prevalence** \| \|; **Time step (DT)** \| **% Difference (Euler method compared to Runge-Kutta´s fourth-order method)** **t = 2020** \| **% Difference (Euler method compared to Runge-Kutta´s fourth-order method)** **t = 2040**; DT 0.0078125 \| -0.027% \| -0.005%; DT 0.015625 \| -0.053% \| -0.008%; DT 0.03125 \| -0.105% \| -0.017%; DT 0.0625 \| -0.212% \| -0.035%; DT 0.125 \| -0.425% \| -0.069%; DT 0.25 \| -0.859% \| -0.142%; DT 0.5 \| -1.750% \| -0.300%; DT 1 \| -3.618% \| -0.673% | At the simulation midpoint (2020), the % difference exceeds 1% for DT = 0.5 and DT = 1, suggesting the need for lower DT values to accurately capture the dynamics. For all other DT values, the % difference remains below 1% at both the midpoint (2020) and endpoint (2040), though differences increase with larger DTs, further supporting the use of smaller DT values. |

DT: delta time

**References**

1.	Sterman JD. Business Dynamics Systems Thinking and Modeling for a Complex World Jeffrey J. Shelsfud. Irwin McGraw-Hill; 2000.
