# Supplementary material for: Retrofitting fiscal policies to reduce the double burden of malnutrition in Peru: A system dynamics modelling study
Source: PLoS Med. 2026 Aug 13;23(8):e1005167. doi: 10.1371/journal.pmed.1005167 (PMC13472416; doi:10.1371/journal.pmed.1005167)
Supplement: Supplementary file 12 [file pmed.1005167.s012.docx]

**S4 Table. Variables and their parameters used in the probabilistic sensitivity analysis**

| **Variable^1^** | **Effect estimate** | **SD/SE** | **Lower limit of 95% CI** | **Upper limit of 95% CI** | **Min^2^** | **Max^2^** | **Source** |
| --- | --- | --- | --- | --- | --- | --- | --- |
| cross price elasticity F and V on SSB demand | 0.0675 | 0.1190 |  |  | -0.2895 | 0.4245 | Pereda, 2024[1] |
| cross price elasticity F and V on UPF demand | 0.0645 | 0.1180 |  |  | -0.2895 | 0.4185 | Pereda, 2024[1] |
| cross price elasticity SSB on UPF demand | -0.0200 | 0.0630 |  |  | -0.2090 | 0.1690 | Pereda, 2024[1] |
| cross price elasticity UPF on SSB demand | 0.0090 | 0.0580 |  |  | -0.1650 | 0.1830 | Pereda, 2024[1] |
| EE F and V prices on stunting | -0.5933 | 1.440^3^ | -3.4167 | 2.2300 | -4.9148 | 3.7281 | Headey, 2019 [2] |
| EE F and V prices on overweight | -0.435 | 0.470^4^ |  |  | -1.8447 | 0.9747 | Han, 2011[3] |
| EE Juntos cash on overweight | -0.0006 | 0.0002^3^ | -0.0010 | -0.0002 | -0.0012 | 0.0000 | Fernald, 2008[4] |
| EE Juntos cash on stunting | -0.0009 | 0.0002^3^ | -0.0013 | -0.0004 | -0.0016 | -0.0002 | Fernald, 2008[4] |
| EE Juntos coverage on overweight children | -0.0296 | 0.0270^3^ | -0.0823 | 0.0233 | -0.1105 | 0.0512 | Andersen, 2015[5] |
| EE Juntos coverage on stunting | -0.0523 | 0.0291^3^ | -0.1094 | 0.0045 | -0.1395 | 0.0349 | Andersen, 2015[5] |
| EE Juntos TPI expansion | -0.065 | 0.034 |  |  | -0.1670 | 0.0370 | MIDIS, 2024[6] |
| EE SSB consumption on overweight | 0.0054 | 0.0013 |  |  | 0.0015 | 0.0094 | Ferretti, 2019[7] |
| EE UPF consumption on overweight | 0.0054 | 0.0013 |  |  | 0.0015 | 0.0094 | Ferretti, 2019[7] |
| SSB own price elasticity [poor] | -1.3280 | 0.0990 |  |  | -1.6250 | -1.0310 | Paraje, 2016[8] |
| SSB own price elasticity [rich] | -1.2010 | 0.0760 |  |  | -1.4290 | -0.9730 | Paraje, 2016[8] |
| UPF own price elasticity [poor] | -1.3280 | 0.0990 |  |  | -1.6250 | -1.0310 | Paraje, 2016[8] |
| UPF own price elasticity [rich] | -1.2010 | 0.0760 |  |  | -1.4290 | -0.9730 | Paraje, 2016[8] |
| Stunting faltering TP, ages 0to4 | 0.2803 | 0.0138 | 0.2533 | 0.3072 | 0.2390 | 0.3215 | Own estimations |
| Stunting faltering TP, ages 5to9 | 0.0876 | 0.0084 | 0.0712 | 0.1040 | 0.0626 | 0.1127 | Own estimations |
| Stunting faltering TP, ages 10to14 | 0.0909 | 0.0112 | 0.0690 | 0.1128 | 0.0574 | 0.1244 | Own estimations |
| Stunting recovery TP, ages 0to4 | 0.3782 | 0.0299 | 0.3196 | 0.4369 | 0.2885 | 0.4680 | Own estimations |
| Stunting recovery TP, ages 5to9 | 0.7636 | 0.0353 | 0.6945 | 0.8328 | 0.6578 | 0.8695 | Own estimations |
| Stunting recovery TP, ages 10to14 | 0.2780 | 0.0287 | 0.2217 | 0.3342 | 0.1919 | 0.3641 | Own estimations |
| Overweight faltering TP, ages 0to4 | 0.1771 | 0.0130 | 0.1516 | 0.2025 | 0.1381 | 0.2160 | Own estimations |
| Overweight faltering TP, ages 5to9 | 0.2532 | 0.0171 | 0.2197 | 0.2866 | 0.2019 | 0.3044 | Own estimations |
| Overweight faltering TP, ages 10to14 | 0.0738 | 0.0112 | 0.0519 | 0.0957 | 0.0403 | 0.1073 | Own estimations |
| Overweight recovery TP, ages 0to4 | 0.6934 | 0.0453 | 0.6046 | 0.7821 | 0.5576 | 0.8292 | Own estimations |
| Overweight recovery TP, ages 5to9 | 0.6888 | 0.0394 | 0.6115 | 0.7661 | 0.5705 | 0.8072 | Own estimations |
| Overweight recovery TP, ages 10to14 | 0.3791 | 0.0301 | 0.3202 | 0.4380 | 0.2889 | 0.4693 | Own estimations |
| OR wealth overweight faltering TP adults | 0.5491 | 0.1399 | 0.3396 | 0.8879 | 0.1295 | 0.9688 | Own estimations |
| OR wealth overweight faltering TP children | 1.0380 | 0.1296 | 0.8147 | 1.3225 | 0.6493 | 1.4267 | Own estimations |
| OR wealth overweight recovery TP adults | 2.1752 | 0.8301 | 1.0894 | 4.3432 | -0.3150 | 4.6654 | Own estimations |
| OR wealth overweight recovery TP children | 1.6103 | 0.3999 | 1.0071 | 2.5747 | 0.4106 | 2.8100 | Own estimations |
| OR wealth stunting faltering TP adults | 3.0320 | 0.7154 | 1.9383 | 4.7427 | 0.8858 | 5.1782 | Own estimations |
| OR wealth stunting faltering TP children | 4.0999 | 0.5152 | 3.2127 | 5.2321 | 2.5544 | 5.6454 | Own estimations |
| OR wealth stunting recovery TP adults | 0.7231 | 0.1890 | 0.4420 | 1.1830 | 0.1560 | 1.2902 | Own estimations |
| OR wealth stunting recovery TP children | 0.6122 | 0.0812 | 0.4734 | 0.7917 | 0.3686 | 0.8558 | Own estimations |

^1^A normal distribution was fitted for all variables

^2^Min and max values were estimated under the assumption that they fall within 3 standard deviations of the mean

^3^Standard errors were estimated from the 95% CIs using the formula (upper limit – lower limit)/3.92. We assumed that the distribution of the effect estimate approximates a normal distribution with standard deviation equal to the standard error of the mean (central limit theorem)

^4^Standard error of elasticity is approximated from standard error of marginal effects by adjusting it to the ratio of elasticity over marginal effects, assuming elasticity is a linear function of marginal effects

EE: Effect Estimate, F and V: Fruit and Vegetables,SSB: Sugar Sweetened Beverages, UPF: Ultra-processed Food, TP: Transition Probability, OR: Odds Ratio, TPI = Early Childhood Transfer of Juntos

**References**

1.	Pereda PC, Moz-Christofoletti MA, Duran AC, Louzada MLaC, Ng SW. Equity-Driven fiscal Policies: Taxing Ultra-Processed products and subsidizing minimally processed foods. Food Policy. 2024.

2.	Headey DD, Alderman HH. The Relative Caloric Prices of Healthy and Unhealthy Foods Differ Systematically across Income Levels and Continents. J Nutr. 2019;149(11):2020-33. doi: 10.1093/jn/nxz158. PubMed PMID: 31332436; PubMed Central PMCID: PMCPMC6825829.

3.	Han E, Powell LM. Effect of food prices on the prevalence of obesity among young adults. Public Health. 2011;125(3):129-35. Epub 20110126. doi: 10.1016/j.puhe.2010.11.014. PubMed PMID: 21272902.

4.	Fernald LCH, Gertler PJ, Neufeld LM. Role of cash in conditional cash transfer programmes for child health, growth, and development: an analysis of Mexico's Oportunidades. Lancet. 2008;371(9615):828-37. doi: 10.1016/s0140-6736(08)60382-7. PubMed PMID: WOS:000253866500030.

5.	Andersen CT, Reynolds SA, Behrman JR, Crookston BT, Dearden KA, Escobal J, et al. Participation in the Juntos Conditional Cash Transfer Program in Peru Is Associated with Changes in Child Anthropometric Status but Not Language Development or School Achievement<sup>1-4</sup>. J Nutr. 2015;145(10):2396-405. doi: <http://dx.doi.org/10.3945/jn.115.213546>. PubMed PMID: 606519098.

6.	social P-MdDeI. Evaluación de impacto de la transferencia a primera infancia (TPI) sobre la cobertura del paquete integral de salud, la prevalencia de anemia y desnutrición crónica infantil 2024 [cited 2024 November 30]. Available from: <https://evidencia.midis.gob.pe/evaluacion-transferencia-primera-infancia-juntos/>.

7.	Ferretti F, Mariani M. Sugar-sweetened beverage affordability and the prevalence of overweight and obesity in a cross section of countries. Global Health. 2019;15(1):30. Epub 20190418. doi: 10.1186/s12992-019-0474-x. PubMed PMID: 30999931; PubMed Central PMCID: PMCPMC6472017.

8.	Paraje G. The Effect of Price and Socio-Economic Level on the Consumption of Sugar-Sweetened Beverages (SSB): The Case of Ecuador. PLoS One. 2016;11(3):e0152260. Epub 20160330. doi: 10.1371/journal.pone.0152260. PubMed PMID: 27028608; PubMed Central PMCID: PMCPMC4814055.
