# Supplementary material for: Retrofitting fiscal policies to reduce the double burden of malnutrition in Peru: A system dynamics modelling study
Source: PLoS Med. 2026 Aug 13;23(8):e1005167. doi: 10.1371/journal.pmed.1005167 (PMC13472416; doi:10.1371/journal.pmed.1005167)
Supplement: Supplementary file 13 [file pmed.1005167.s013.docx]

**S2 Figure. Policy mechanisms showing changes in (A) Juntos Basic Transfer coverage in children, (B) Percentage change in Juntos Basic Transfer cash amount, (C) Juntos Early Childhood Transfer coverage in children under-5, and (D) Fruit and vegetable subsidy rate, under different scenarios.**

SSB = sugar-sweetened beverage. UPF = ultra-processed food. SES: Socioeconomic status
