# Supplementary material for: Retrofitting fiscal policies to reduce the double burden of malnutrition in Peru: A system dynamics modelling study
Source: PLoS Med. 2026 Aug 13;23(8):e1005167. doi: 10.1371/journal.pmed.1005167 (PMC13472416; doi:10.1371/journal.pmed.1005167)
Supplement: Supplementary file 14 [file pmed.1005167.s014.docx]

**S5 Table. Cumulative person-years lived with overweight in adults of reproductive age by 2040 under sensitivity analysis assuming Juntos effect on overweight in adults, equal to the Juntos effect on overweight in children, for Scenarios A and B, overall and by socioeconomic group.**

| Revenue Reallocation Scenario | Socioeconomic group | Cumulative Person-Years Lived with Overweight in Adults (Million), Median (95% UI) | Difference in cumulative Person-Years Lived with Overweight in Adults in sensitivity analysis compared to main analysis, (Thousands), Median (95% UI) |
| --- | --- | --- | --- |
| *Current SSB Tax* | | | |
| A. Juntos Basic Transfer coverage | Poorest | 51 (49 – 52) | -2734 (-5163, 596) |
|  | Richest | 164 (161 – 167) | 550 (988, -37) |
|  | *Total* | 215 (210 – 219) | -2184 (-4175, 559) |
| B. Juntos Basic Transfer cash | Poorest | 49 (48 – 51) | -2299 (-2842, -1434) |
|  | Richest | 166 (165 – 166) | 0 (0, 0) |
|  | *Total* | 215 (213 – 217) | -2299 (-2842, -1434) |
| *Additional SSB and UPF taxes* | | | |
| A. Juntos Basic Transfer coverage | Poorest | 38 (34 – 42) | -1867 (-1995, -864) |
|  | Richest | 122 (108 – 137) | -196 (-389, -472) |
|  | *Total* | 161 (142 – 179) | -2063 (-2384, -1336) |
| B. Juntos Basic Transfer cash | Poorest | 38 (34 – 41) | -1611 (-1335, -1897) |
|  | Richest | 123 (109 – 138) | 0 (0, 0) |
|  | *Total* | 160 (142 – 179) | -1611 (-1335, -1897) |

SSB: Sugar-Sweetened Beverages; UPF: Ultra-Processed Food; UI: Uncertainty Interval
