# Supplementary material for: Retrofitting fiscal policies to reduce the double burden of malnutrition in Peru: A system dynamics modelling study
Source: PLoS Med. 2026 Aug 13;23(8):e1005167. doi: 10.1371/journal.pmed.1005167 (PMC13472416; doi:10.1371/journal.pmed.1005167)
Supplement: Supplementary file 15 [file pmed.1005167.s015.docx]

**S1 Spanish Abstract**

**Spanish Language Abstract**

**Antecedentes**

El Perú enfrenta una doble carga de malnutrición, caracterizada por la coexistencia de sobrenutrición y desnutrición. Aunque se han implementado diferentes políticas fiscales para abordar estos desafíos nutricionales, han sido diseñadas e implementadas en forma aislada, limitando su potencial de impacto sinérgico. Este artículo investiga el posible impacto de la adaptación de la tributación de alimentos y bebidas poco saludables para acciones de 'doble función', destinando los ingresos a transferencias condicionadas y a subvenciones para frutas y verduras y evaluando su efecto sobre el sobrepeso y la desnutrición crónica en el Perú.

**Métodos y resultados**

Se desarrolló un modelo de dinámica de sistemas para simular el sobrepeso y la desnutrición crónica en niños y adultos en edad reproductiva, utilizando fuentes de datos secundarias y estimaciones de efectos publicadas. Se modeló varios escenarios de política fiscal, en concordancia con las recomendaciones de la OMS y el panorama político del Perú. Se realizó varias pruebas para aumentar la confianza en el modelo. Los escenarios de política fiscal pueden tener efectos beneficiosos sobre el sobrepeso en comparación con el escenario habitual, principalmente a través del impacto de impuestos adicionales sobre alimentos y bebidas poco saludables, lo que reduciría el número acumulado de años-persona vividos con sobrepeso en adultos entre el 2024 y el 2040 en -58,6 millones (Intervalo de Incertidumbre [II] del 95% [-77,7, -37,9]), principalmente entre los más ricos. Reinvertir los ingresos fiscales para aumentar la cantidad de efectivo ofrecida a través de la actual transferencia condicional de efectivo en el Perú podría reducir el número acumulado de años de persona vividos con desnutrición crónica de la niñez en hasta 876 mil (II 95% [-1139, -567]) para el 2040, con el 82% de estos casos entre los más pobres. Este estudio se basa en varios supuestos sobre los impactos y respuestas de las políticas y no incorpora análisis de costo-efectividad debido a que exclusyó grupos de edad mayores.

**Conclusiones**

La adaptación de la tributación de alimentos y bebidas poco saludables para reasignar los ingresos a programas de bienestar específicos como las transferencias de efectivo, tiene un potencial considerable para lograr impactos 'dobles' en la sobrenutrición y la desnutrición en el Perú.
