# Supplementary material for: Retrofitting fiscal policies to reduce the double burden of malnutrition in Peru: A system dynamics modelling study
Source: PLoS Med. 2026 Aug 13;23(8):e1005167. doi: 10.1371/journal.pmed.1005167 (PMC13472416; doi:10.1371/journal.pmed.1005167)
Supplement: Supplementary file 16 [file pmed.1005167.s018.docx]

Thank you for the opportunity to revise our paper and to the reviewers for their valuable feedback. We provide below a point-by-point response to all reviewer comments and present the relevant text from the manuscript, highlighting newly added text in italics.

**Reviewer #1: Thank you for the opportunity to review this interesting and impactful modeling study of redirecting sugar-sweetened beverage and junk food tax revenue toward fruit and vegetable subsidies in Peru. This well-written article addresses an innovative and important topic. Below are some suggestions for improving the rigor, presentation, and interpretation in rough order of priority:**

**The basic premise of the study seems to be that allocating unhealthy food tax revenue toward healthy food is more impactful than not allocating - and the more is allocated, the better. This is a somewhat self-evident finding and risks drawing an erroneous policy conclusion because it does not consider what the revenue would be spent on otherwise. There is a possibility that unhealthy food tax revenue could instead be allocated to an activity that generates even more population health benefits than, say, subsidizing fruits and vegetables. An example might be immunization against diarrheal diseases, which is extremely cost-effective at reducing morbidity/mortality and can reduce malnutrition/stunting by addressing nutrient malabsorption from diarrhea. Could the authors comment on: (A) what unhealthy food tax revenue is currently spent on, (B) what are the marginal opportunities of Peruvian healthcare (e.g., health services that are not yet funded), (C) what is the cost-effectiveness of spending it on the proposed interventions (in terms of money per QALY gained, DALY averted, etc.), and (D) how this compares to cost-effectiveness benchmarks for Peruvian government health spending?**

Thank you for this comment.

Revenue from Peru’s existing SSB tax is not earmarked for any specific health or nutrition programmes. As a result, these funds flow into the general budget and are not trackable to particular activities. We have clarified this in the revised manuscript, line 137-138: “Moreover, Peru does not earmark the revenue from its SSB tax for any specific health or nutrition programmes” . The absence of earmarking is a key motivation for our study, particularly given WHO recommendations to earmark unhealthy food and beverage tax revenues as a strategy to improve their effectiveness in promoting healthier diets. Importantly, our study offers a novel contribution by exploring how earmarking can be used to deliver double-duty actions, which has not been previously examined.

We addressed this gap by modelling tax revenue reallocation to Juntos, a conditional cash transfer programme, which can improve nutrition and health outcomes through various mechanisms, including conditionalities promoting immunisation, growth monitoring, utilisation of preventive health services, and others. This consideration of non-food revenue pathways adds further novelty to our study. We additionally modelled fruit and vegetable subsidies, as they are a commonly recommended earmarking option for unhealthy food and beverage taxes, although its double-duty potential has not been previously explored. Based on these points and responding to the reviewer’s comment that these revenues could be used to fund other health interventions, we have added the following sentence to the Discussion: lines 465-467: *“Fourth, our study does not model all potential double-duty actions in Peru but rather focuses on flagship fiscal policies that have strong DBM potential due to existing evidence of their impacts on nutrition outcomes[24, 26-32]”*

We appreciate the reviewer’s suggestion for a cost-effectiveness analysis. However, we did not conduct one because it would not be appropriate given our study’s focus on children and adults of reproductive age. DALYs and QALYs associated with overweight/obesity accrue predominantly in older adults, which would systematically underestimate the benefits of overnutrition-related interventions in our model, making comparisons between undernutrition and overnutrition pathways misleading. We have now clarified this as a limitation in our discussion: Lines 470-474: “Finally, *we did not conduct cost-effectiveness analysis*, *as summary measures of population health needed for this analysis would have been underestimated in this model due to its age group coverage*.”

**It is great that the authors conducted both univariate (+/- 10% on individual assumptions) and multivariate (PSA) sensitivity analyses. However, it is critical to then propagate the uncertainty ascertained in multivariate sensitivity analysis to reported outcomes. All reported results in the text and tables should have 95% UIs, and all figures should be graphed with uncertainty bands. Currently, this is implemented inconsistently throughout the manuscript.**

Thank you for your comment. We have now incorporated uncertainty intervals from our probabilistic sensitivity analysis in Figure 2. Figure 3 and newly added Figure 4 also have uncertainty intervals.

**Furthermore, there may be internal inconsistencies in when uncertainty is considered vs. not. E.g., in Figure 3, the authors have either neglected to graph confidence bands for some curves, or else the confidence bands are unreasonably tight for some bands so as to be invisible (e.g., adult overweight forecasts for the wealthiest quintile in Scenarios A and B; for the poorest quintile in Scenarios B and C, child stunting forecasts for both quintiles under all scenarios except for Scenario D). The authors should revisit assumptions and PSA design to ensure all main sources of uncertainty are captured, including uncertainty in baselines and their forecasts even in the absence of revenue reallocation.**

Thank you for this helpful recommendation. We have updated our PSA to incorporate uncertainty not only in policy-induced effects but also in baseline values. Specifically, we added uncertainty for all transition probabilities for overweight and stunting (faltering and recovery) in children, as well as for the ORs capturing differences in transition probabilities between the poorest and wealthiest quintiles. As a result, uncertainty bands are now visible for groups with greater underlying variability, particularly for stunting in the poorest quintile. For business‑as‑usual scenarios and for the wealthiest quintiles, uncertainty intervals remain very narrow due to larger sample sizes in the underlying data and the limited accumulation of uncertainty in early simulation years; hence the bands appear visually minimal.

To ensure consistency in reporting, we have also added uncertainty intervals to Figure 2.

**For the scenario set, the authors made conservative assumptions of no effect in groups where no evidence of an effect exists. While this seems reasonable for a main analysis, it would be apt to vary this assumption in sensitivity analysis.**

Thank you for this suggestion. We have now added sensitivity analysis, where we also assume that Juntos scenarios (expanding coverage and cash amount) would have a positive effect on adult overweight, equal in magnitude to the effect it had to child overweight. We do not assume an effect of the Juntos Early Childhood Transfer programme on adults, as this scenario explicitly targets children under-5.

We have now reported this sensitivity analysis in the methods, lines 285-286 and 292-293: *“We further conducted sensitivity analysis assuming an effect on adult overweight, equal to that for child overweight.”*

The sensitivity analysis results are presented in S5 Table and in the Results section of the revised manuscript in lines 388-391: *“Sensitivity analysis assuming that expansion of coverage and cash amount of Juntos also benefits overweight in adults showed an additional reduction of maximum 2063 (-2384 – -1336) and 1611 (-1335 – -1897) thousand overweight person-years among adults under the additional SSB/UPF Scenarios A and B respectively (S5 Table)”*

**In Figure 2A, could the authors explain why there is a revenue downturn between 2025 and 2030? What drives this?**

Thanks for this observation. The downturn between 2025 and 2030 is due to the taxes’ effect on purchasing behaviour, i.e. reduction in purchases of taxed products following implementation, which recovers within 5 years of the tax implementation.

This is described in the Results section, lines 343-346: “A temporary revenue dip may occur after the first year of implementation, due to the taxes’ effects on purchasing behaviour, but the model predicted annual revenue recovery within 5 years, followed by sustained long-term growth (Figure 2A)”

**Also in Figure 2A, could the authors graph revenue time-series for the other revenue generation scenarios for ease of comparison? It might be nice to show these on a per capita or per child basis to contextualize the ~3Bn magnitude for Peru, and show how things are trending after adjusting for population growth.**

Thank you for this comment. As described in Figure 1, we have only modelled two revenue generation scenarios, i.e. the “Current SSB tax” scenario, which refers to the current SSB tax of 25% on SSBs with >6g sugar/100mL and 17% on SSBs with <6g sugar/100mL, and the “Additional SSB and UPF taxes” scenario, which refers to a SSB tax of 30% on SSBs with >6g sugar/100mL, 17% on SSBs with <6g sugar/100mL and 10% on ultra-processed foods . Both of these revenue generation scenarios are presented in Figure 2A.

As per the reviewer’s suggestion, we have now updated Figure 2A to show revenue generation per capita.

**Table 1 might be better displayed as a floating bar chart or box and whiskers chart to make it easier to take in, considering values down the columns all have the same units so could be graphed together. Colors and groupings could be used to visually organize the information.**

Thank you for this suggestion. We have now replaced Table 1 with a floating bar chart (Figure 4), as per the reviewer’s recommendation.

**In Supplementary Table S2 showing the list of scenarios, it would be helpful to add columns specifying which age groups the intervention is assumed to impact, and the assumed effect sizes. In their main analysis.**

We have now added two additional columns to Supplementary Table S2, as recommended. Specifically, we added a column named *Outcomes targeted*, which highlights the outcomes and age groups each revenue reallocation scenario has targeted and a column named *Effect estimates* presenting corresponding effect estimates used in the main analysis for each outcome. Further detailed information on sources and interpretations of effect estimates is additionally presented in Table S3.

**There appears to be a table misnumbering issue in the Appendix. The second Supplementary Table S1 should be S2, S2 should be S3, etc.**

Thank you, we have now updated the numbering appropriately.

**Possible typographical error: "We adjusted the impact of Juntos scenarios on overweight and stunting using a an annually varying discount rate" - extra "a."**

Thank you, we have now removed the extra “a”.

**Reviewer #2: In line 64, define SSBs (as this is the first time they are mentioned in the manuscript).**

SSBs are first mentioned and defined in line 93 of the main manuscript.

**In line 65, for the sentence 'have enormous potential as double-duty actions', I suggest adding a reference.**

We have added a citation ([17]) in this line.

**Line 66, for the sentence 'These policies, usually implemented in silos', I suggest emphasising why this is a problem in the context of Peru before jumping to the solution.**

We have now added a sentence in the Introduction clarifying that this siloed implementation stems from the division of responsibility for overnutrition and undernutrition across diverse departments, in lines 119-121: *“This primarily stems from the division of responsibility across government departments, with overnutrition overseen by the Ministry of Health, whist undernutrition falls under social policy programmes led by the Ministry of Development and Social Inclusion.”*

**Why are these two potential double duty actions (DDA) chosen? Are DDAs in Peru likely to have the greatest potential impact on the double burden of malnutrition (DCM)? Why study these DDAs and not others? Is the fact that the DDAs studied are implemented in silos the problem? Or is there no policy for the double burden of malnutrition in Peru that brings together government actions with the potential to become DDA? Were the potential DDAs analysed in the article designed to address public problems other than DCM? Is there no evidence of the impact of the potential DDA analysed in this study on the achievement of the results proposed in its creation? Is there no evidence of the impact of the potential DDA analysed in this study on DCM? I suggest better defining and explaining the context of the issue addressed by the study.**

Thank you for this detailed comment.

The selection of policy scenarios in our study was guided by both global recommendations and the specific policy context in Peru. At the global level, the WHO identifies fiscal policies as a key lever to promote healthier diets and explicitly recommends using revenue earmarking to enhance the effectiveness of unhealthy food and beverage taxes, particularly through options such as fruit and vegetable subsidies. In parallel, seminal work on double‑duty actions (Hawkes, 2020) highlights a gap in using food environment policies, including SSB taxes, to address both sides of the double burden of malnutrition, as such policies have traditionally focused only on overnutrition. Together, these recommendations motivated our focus on fiscal measures and on exploring how revenue reallocation could strengthen the double‑duty potential of existing taxes.

Within Peru, we selected specific policies based on their demonstrated effectiveness and their relevance within ongoing policy debates. Although Peru introduced its SSB tax in 2018, discussions about its impact remain active. Nevertheless, international and regional evidence suggests that unhealthy food and beverage taxation can substantially improve diets. At the same time, Juntos is widely recognised both nationally and globally as a major contributor to Peru’s rapid decline in stunting between 2008 and 2016, yet recent stagnation in stunting levels may raise concerns about underinvestment in the programme. Recent reforms to Juntos, including the introduction of the Transfer for Early Childhood (Transferencia a la Primera Infancia) in 2021, also signal an important policy window for strengthening its implementation. These factors collectively informed our choice to model these fiscal scenarios as the most relevant, evidence‑based, and timely options for Peru.

We now more clearly describe this rationale for policy selection in our Introduction and Methods as follows,

Introduction, lines 121-126: *We focus on the Juntos cash transfer program (2005) and a tiered SSB tax (2018) as examples of policies implemented in the country with potential to be retrofitted as double duty actions. Given robust national or international evidence of their independent impacts [24, 26-32], and recent reforms, such as the Early Childhood Transfer of Juntos, these policies are both evidence-based and timely options for exploring double-duty potential of fiscal policies in Peru.”*

Methods, lines 213-217: *“Selection of fiscal policies was informed by WHO recommendations identifying unhealthy food and beverage taxation and revenue earmarking to fruit and vegetable subsidies as key levers to promote healthier diets,[16] as well as increasing calls for food environment policies, such as SSB taxation, to act as double-duty actions[17].”*

We have also added text in the Discussion clarifying that while other interventions could also be modelled, chosen policies have strong evidence of impact, making them appropriate for this study: lines 465-467: “Fourth, our study does not model all potential double-duty actions in Peru but rather focuses on flagship fiscal policies that have strong DBM potential due to existing evidence of their impacts on nutrition outcomes. [24, 26-32]”

In Peru, neither the SSB tax nor Juntos was originally designed as a double‑duty action. Juntos was developed as a conditional cash transfer programme to break the intergenerational cycle of poverty via increased and sustained access to education and health services. Its effectiveness on several outcomes, including substantial reductions in child stunting, has been well documented. In contrast, while Peru’s SSB tax is intended to improve dietary behaviours and reduce overnutrition, its impact on overweight and obesity outcomes has not yet been quantified in the Peruvian context. Crucially, no existing policy in Peru has been explicitly designed or implemented as a double‑duty action, nor have the potential double‑duty effects of either Juntos or the SSB tax been previously examined. This means the question is not that potential DDAs operate in silos, but rather that Peru currently lacks an integrated policy approach specifically targeting the double burden of malnutrition. This absence of a coordinated double‑duty policy framework, despite the individual evidence base for the policies’ intended outcomes, creates a clear gap and an opportunity for research to guide further policy innovation. Our study contributes to filling this gap by assessing how fiscal policies with established effects in their primary domains could be leveraged for double‑duty impact.

We have now updated the introduction to reflect these points more clearly:

Lines 118: *“… with no policies explicitly designed or implemented as double-duty actions”*

Line 136-137: *“Peru’s tiered SSB tax was introduced in 2018 in response to the country’s increasing obesity rates.”*

Lines 140-144: “*Moreover, Peru does not earmark revenue from its SSB tax for any specific health or nutrition programmes. Although the impacts of the Peruvian SSB tax on overweight and obesity have not yet been evaluated, substantial modelling and observational evidence from other settings, including in Latin America, indicate its strong potential to reduce SSB intake and associated overweight and obesity prevalence [23-25].”*

Lines 147-152: “*Although previous research has extensively examined the impact of SSB taxes on dietary intake and overnutrition outcomes[23, 24, 37], very few studies have explored revenue-earmarking strategies, and those that did focused on limited scenarios primarily targeting overnutrition[38]. No prior studies have explicitly examined the potential of retrofitting existing nutrition‑relevant fiscal policies, such as Peru’s SSB tax and Juntos, to jointly target the DBM”*

In summary, we focused on the SSB tax and Juntos because they are flagship policies in Peru with robust evidence of independent impact, both internationally and within Peru. The novelty of our work lies in retrofitting these established policies for enhanced double-duty impact, an approach aligned with WHO recommendations on leveraging fiscal measures and revenue use for healthier diets, but one that has not been empirically evaluated in Peru or elsewhere.

**In line 73, in the sentence 'Peru has implemented fiscal policies to address undernutrition and overnutrition,' I suggest adding "separately" or 'in silos' at the end.**

Thank you for this suggestion. We have now revised this line to: *“*Peru has implemented fiscal policies to address undernutrition and overnutrition, *but these have been designed and implemented separately…”*

**I suggest dividing or rewriting the paragraph contained between lines 73-85. The combination of both potential DDAs reads awkwardly. Both government actions were created with specific and 'different' objectives (Juntos: 'to reduce intergenerational poverty in Peru'; SSB taxes in Peru: to protect public health by discouraging the consumption of these products due to their associated harms... It would be good to highlight these in the text to guide the order of the paragraphs.**

Thank you for this comment. We have substantially revised the Introduction based on all reviewer suggestions. We have now clearly articulated the specific and different objectives of Juntos and the SSB tax in this paragraph.

Line 127: *“*Juntos *was developed to tackle poverty, especially in rural areas …”*

Line 136: *“Peru’s* tiered SSB tax *was introduced* in 2018 *in response to the country’s increasing in obesity rates.”*

**Line 86 Add reference or data (prevalence) for the sentence 'Despite Peru's high DBM prevalence'; I suggest that they be included in the first paragraph of the introduction.**

We have now added text in the first paragraph discussing the epidemiology of stunting, overweight, and DBM in Peru.

Lines 98-103: *“This phenomenon is exemplified in Peru, which has faced a significant increase in overweight and obesity over the last decades, with 65% of adults classified as overweight in 2022 [8-10]. At the same time, Peru has a long history of stunting, and despite significant reductions since the 2000s, recent data indicate that progress has stagnated over the last five years[5]. These trends have led to DBM at both household and population levels in Peru, with persistent inequalities[6. 7].”*

**The statement on lines 86-87 "policies targeting undernutrition and overnutrition appear to be designed and implemented separately, limiting their potential for synergistic impact" . Currently, SSB 87 tax revenue is not redirected to improve access to healthier diets or reduce poverty, a major driver of poor diets, and the opportunity to achieve double-duty benefits remains underutilized". I suggest that this be moved up to paragraph 2 and that the issue addressed by the study be better explained before moving on to the solutions.**

We have now restructured the introduction and removed this sentence. We hope that the revised introduction clearly explains the issue addressed before moving on to the solutions.

**Lines 92-94 mention a "systems dynamics approach"; it would be ideal to add a brief sentence on why this approach is appropriate for addressing the problem identified in the study.**

**Line 97: I suggest expanding on the explanation of why the system dynamics model (and not another) is appropriate for this study.**

Thank you for both of these suggestions. We have now added a new section in the Methods on system dynamics modelling, providing an overview of the method and detailing why it was appropriate for this study, in lines 164-183:

*“System dynamics modelling is a differential equation-based simulation modelling approach concerned with explaining dynamic behaviour, i.e. how problems change over time[39]. It is grounded on three main principles: feedback, accumulations, and delays. Feedback denotes situations in which an effect generates an outcome that, in turn, influences its original cause, creating causal loops. Accumulations, or stocks, refer to the gradual change of a system state, arising when rates of decrease and increase differ over time. Finally, delays capture the phenomenon where the effect of one variable on another does not occur instantaneously, but after a temporal lag[39].*

*These characteristics constitute system dynamics as a highly suitable approach for this study. First, the capacity of system dynamics to capture feedback mechanisms enables modelling of the intergenerational transmission of nutrition outcomes, from children to adults and back to children. Previous modelling analysis of the DBM in Peru highlight this important gap and recommends that future modelling work should incorporate the intergenerational transmission of overnutrition and undernutrition[40]. Second, the modelling of accumulations and delays allow us to capture important temporal lags that often characterise changes in prices and market conditions. There are limited system dynamics models that explore the dynamics of SSB taxation[41], with only one study explicitly incorporating delays in the analysis[42]. However, this study estimated the impact of SSB taxation on body weight via changes in dietary energy intake, which restricts exploration of both overnutrition and undernutrition outcomes or double-duty scenarios. Finally, given our focus on aggregated population-level dynamics, system dynamics modelling offers a more suitable methodological approach compared to other micro-perspective modelling frameworks[43].*

**In the section on policy selection and in the introduction, it is not clear why the two potential DDAs were chosen (and not other DDAs suggested by the WHO).**

We have now updated these to provide a clearer rationale for the selection of policies in the introduction, lines 121-126, and methods, lines 215-217, as indicated in our earlier response.

**In lines 115-116, it is suggested to clarify the reference point for the second scenario, both the 'SSB tax increase' and the 'new 10% UPF tax'. Do both follow the Colombian model as a reference for this scenario? Add a reference to what Colombia applies.**

Apologies for the confusion. We have revised the sentence to clarify that only the 10% UPF tax refers to Colombia’s example and to add a reference – line 217-218: “…, following Colombia’s example *in the implementation of UPF taxation [36]*.”

**In lines 118-121, it is suggested to explain which criteria are used to establish scenarios A, B, C, and D.**

Alongside the new text in the Introduction outlining our choice of policies in this study, we have now also added text in this section to clarify our selection of scenarios A-D, as per the reviewer’s suggestion: lines 229-232: *“Scenarios A and B were selected in accordance with SDG 1.3, reflecting the goals of ensuring both adequate and sufficient coverage of social assistance, while Scenario C reflects recent changes to the Juntos programme. Scenario D follows WHO recommendations for revenue reallocation to promote healthier diets[16].”*

The magnitude of the scenarios depended on modelled taxation revenue. We have revised the corresponding text in lines 246-252 to add clarity:

“Estimated *tax* revenue was distributed to each of the four revenue allocation *scenarios*. For Juntos coverage expansions (allocation scenarios A and C), the poorer quintile had priority over wealthier quintiles. After achieving full coverage at 100%, excess revenue was left unspent. *For Juntos cash amount increase (allocation scenario B), we estimated the cash increase that the accumulated tax revenue could achieve per household per year.* For fruit and vegetable subsidies (allocation scenario D), we calculated the flat, *population-wide* subsidy rate that estimated SSB/UPF tax revenue could fund, using annual fruit and vegetable sales data from Euromonitor[45]”

**In lines 125-138, in scenario D, it is suggested to specify the target audience for the fruit and vegetable subsidy. Are they the beneficiaries of the JUNTOS programme? The general population? Why was a subsidy for fruit and vegetables chosen, and foods rich in iron and essential amino acids? The districts that receive the JUNTOS programme have the highest rates of IDA and iron deficiency anaemia in the region. Among many factors, one is the poor quality of the diet in terms of the aforementioned nutrients.**

The target audience for the fruit and vegetable subsidy is the general population, not beneficiaries of the JUNTOS programme. We have now replaced the word “universal” that we had previously used to indicate this, with the word “population-wide” for further clarity – line 251: “… we calculated the flat, *population-wide* subsidy rate…”

A subsidy for fruit and vegetables was chosen in accordance with WHO recommendations, as it is now clarified in lines 231-232: *“Scenario D follows WHO recommendations for revenue reallocation to promote healthier diets[16].”*

We appreciate and acknowledge the reviewer’s comment about the problem of anaemia and iron deficiency in Peru. We have now clarified in our study limitations that our study does not capture all relevant malnutrition outcomes, in lines 467-469: *“Similarly, it focuses on overweight/obesity and stunting as DBM indicators, although Peru faces high levels of several additional overnutrition, such as diabetes, as well as undernutrition outcomes, such as anaemia.”*

**In lines 139-151, it is suggested to explain why IMCI and overweight were considered, and why the impact on iron deficiency anaemia (part of the double burden of malnutrition) was not considered, as this is one of the main public health problems in Peru today.**

Thank you for this comment. We acknowledge that the double burden of malnutrition can have multiple manifestations across different overnutrition and undernutrition outcomes. We also acknowledge that anaemia is an important public health problem in Peru today. However, the lack of robust quantitative evidence of the impact of Juntos, or comparable policies, on anaemia across diverse age groups restricts our ability to model anaemia with the same degree of confidence in our assumptions as is possible for stunting. We recommend that future research should also explore impacts on anaemia, especially as more robust causal-inference studies on the impact of policies on anaemia emerge.

We have now clarified this in our Discussion, lines 469-470: *“Future research should explore additional outcomes, particularly as new evidence on policy impacts emerges.”*

**In lines 144-146, the assumption made is debatable. Officials from the Peruvian Ministry of Economy often argue that there is no evidence to demonstrate the impact of existing taxes on SBS, which is why there is no evidence to support their increase. Furthermore, they mention that the increase in SBS consumption and the increase in SP and OB in Peru demonstrate poor impact.**

Apologies for the confusion in this sentence. To clarify, our intention was not to make any claim about the magnitude or direction of the effect of existing SSB taxes prior to 2024. This point is methodological, clarifying that for the period before 2024 we rely on observed trends, and therefore we do not model any additional impact of the tax during those years, as this will overestimate its effects. We have now adjusted the sentence to better clarify this, in lines 258-261: “Before 2024, we assumed that changes in transition probabilities informed by epidemiological data already captured *any potential* effects of existing policies, including the SSB tax (since 2018) and the Juntos programme (since 2005), *therefore no additional policy impacts were modelled*.”

**In lines 232-243, it is suggested that the target audience for scenario D be specified.**

We have now clarified in the suggested section that the subsidy rate targets the overall population – line 356: “… could fund up to a 17% or a 23% *population-wide* subsidy”

**In lines 335-355, it is suggested that the policy recommendations be framed and aligned with current country policies in Peru, such as the National Multisectoral Health Policy to 2030 and the Peru National Development Strategic Plan to 2050. It is also suggested that consideration be given to the potential barriers and facilitators to the implementation of the suggested recommendations.**

Thank you for these suggestions. We have now aligned our recommendations with the suggested policies in our Discussion, by revising the text as follows:

Lines 478-479: “… *and align with targets related to prioritising vulnerable populations, outlined in Peru’s National Development Strategic Plan to 2050[63]*”

Lines 485-487: “*This directly responds to priority L1.1 of Peru’s National Multisectoral Health Policy to 2030 to improve healthy population habits and behaviours[64].”*

Lines 491-492: *“Following recommendations related to policy monitoring and evaluation, outlined in the National Multisectoral Health Policy[64] can respond to these needs”*

We have also expanded on potential barriers and facilitators to the implementation of suggested recommendations, in lines 500-505: *“Ultimately, these recommendations can be achieved by capitalising on existing intersectoral platforms, such as those that enabled recent cross‑departmental health and development guidelines[63, 64], and strengthening collaboration with civil society actors who have been instrumental in implementing relevant policies in Peru, including the SSB tax. At the same time, persistent barriers need to be overcome, including weak alignment of shared policy goals across ministries and sustaining long‑term funding, especially amid periods of political instability.”*

**Reviewer #3: The study is clearly important, but the paper has very serious weaknesses especially with regard to the application of the System Dynamics (SD) approach. The paper needs to be improved in line with the following:**

**-Clarify the contribution of the paper and in which academic literature it is positioned. What is the novelty of the study.**

The novelty of our study lies in examining the potential of retrofitting Peru’s existing nutrition-relevant fiscal policies, specifically sugar sweetened beverage (SSB) taxation and Juntos, to jointly address the double burden of malnutrition. Although the WHO highlights retrofitting existing policies as a promising mechanism for double‑duty actions, no prior studies have assessed how Peru’s current fiscal policies could be reconfigured to generate synergistic impacts. Our work is situated within the wider academic literature on SSB taxation, which largely focuses on impacts on overnutrition and rarely explores the potential of earmarking revenues, particularly for double-duty outcomes. By assessing how coordinated fiscal policy design could advance both undernutrition and overnutrition objectives, our study introduces a new policy question and provides evidence to guide future policy innovation.

We have now added text in our introduction to explicit clarify our contribution and refer to the wider literature on the topic, in lines 145-152: *“The WHO emphasises the potential of retrofitting existing interventions as a viable mechanism for creating double-duty actions[11]. It identifies revenue reallocation as an important mechanism to enhance synergistic impacts of fiscal policies[16]. Although previous research has extensively examined the impact of SSB taxes on dietary intake and overnutrition outcomes[23, 24, 37], very few studies have explored revenue-earmarking strategies, and those that did focused on limited scenarios primarily targeting overnutrition[38]. No prior studies have explicitly examined the potential of retrofitting existing nutrition‑relevant fiscal policies, such as Peru’s SSB tax and Juntos, to jointly target the DBM.”*

**-Include a literature review section including a review of the use of SD in the context of malnutrition and related food policy.**

Thank you for this suggestion. Given the journal’s suggested manuscript format, we cannot add a section explicitly focusing on a literature review. However, we have now added a section in our Methods to further discuss the use of SD in this paper. We cite a relevant literature review that discusses the use of systems thinking approaches in the evaluation of SSB taxes and reiterate the lack of previous SD models with capacity to explore both overnutrition and undernutrition outcomes, as follows:

Lines 177-183: *“There are limited studies that employ system dynamics models to explore the dynamics and impacts of SSB taxation[41]. Only one prior study estimated the impacts of SSB taxation on body weight via changes in dietary energy intake[42], with no exploration of both overnutrition and undernutrition outcomes or double-duty scenarios.”*

**-Provide the rationale for using SD and the justification for the context of the study.**

Thank you for this suggestion. We have now added a new section in our Methods section expanding on our use of SD, including our rationale for its use, in lines 164-183:

*“System dynamics modelling is a differential equation-based simulation modelling approach concerned with explaining dynamic behaviour, i.e. how problems change over time[39]. It is grounded on three main principles: feedback, accumulations, and delays. Feedback denotes situations in which an effect generates an outcome that, in turn, influences its original cause, creating causal loops. Accumulations, or stocks, refer to the gradual change of a system state, arising when rates of decrease and increase differ over time. Finally, delays capture the phenomenon where the effect of one variable on another does not occur instantaneously, but after a temporal lag[39].*

*“These characteristics constitute system dynamics as a highly suitable approach for this study. First, the capacity of system dynamics to capture feedback mechanisms enables modelling of the intergenerational transmission of nutrition outcomes, from children to adults and back to children. Previous modelling analysis of the DBM in Peru highlight this important gap and recommends that future modelling work should incorporate the intergenerational transmission of overnutrition and undernutrition[40]. Second, the modelling of accumulations and delays allow us to capture important temporal lags that often characterise changes in prices and market conditions. There are limited system dynamics models that explore the dynamics of SSB taxation[41], with only one study explicitly incorporating delays in the analysis[42]. However, this study estimated the impact of SSB taxation on body weight via changes in dietary energy intake, which restricts exploration of both overnutrition and undernutrition outcomes or double-duty scenarios. Finally, given our focus on aggregated population-level dynamics, system dynamics modelling offers a more suitable methodological approach compared to other micro-perspective modelling frameworks[43].”*

**-Include a qualitative mapping of the system in the form of the a Causal Loop Diagram (CLD).**

Thank you for this suggestion. We have now included a CLD (S1 Figure) qualitatively presenting the main dynamics of the model, including market feedback loops and intergenerational transmission of nutrition outcomes feedback loops. The CLD is cited in the revised manuscript in lines 194-195.

**-Include a description of the model building process including the main stocks and flows and the rate equations.**

We have now added a new section in the Methods, providing an overview of the modelling process, before expanding with further details in the rest of the Methods. The newly added section also provides a description of the main stocks and flows comprising our model. Given the public health audience of the journal, we decided to not include detailed equations in the main manuscript. However, equations for all stocks, flows, and auxiliary variables are presented in detail in the Supplementary Material (S4 Text). We have now clearly reiterated this in this section.

Lines 184-197:

*“Overview of modelling process*

*We developed a system dynamics model in Vensim DSS 10.1.3 in two iterative steps. First, we developed the model’s population component, comprising a co-flow structure tracking three aging chains of Peru’s overall, stunted, and overweight populations, based on Sterman's population dynamics formulations[39] and building on our previous modelling work on the DBM[44]. We then developed the model’s policy component, comprising three main market stock and flow structures, representing the market size of SSBs, UPFs, and fruit and vegetables, as well as a fourth stock and flow structure, representing tax revenue accumulation. We manually calibrated the model to reproduce historical trends of adult overweight and under-5 stunting, using data from Peru’s Demographic and Health Surveys (2000-2020), and historical market size data from Euromonitor (2000-2024) [45], evaluating quality of fit by visually comparing simulated outputs with corresponding reference modes. A causal loop diagram depicting a summarised overview of the model structure is presented in S1 Figure. Detailed equations of stock and flow formulations are presented in S4 Text. The modelling process is described in more detail below.”*

**-Provide more details about the data collection process, data sources, and how the model was calibrated.**

Our study only uses publicly available secondary data. Thus, there was no additional data collection process beyond retrieving these data from online sources.

All our data sources, including values, units, and any potential modifications for model use, are described in detail in the Supplementary Material (S2 Text and S3 Table), which we cite throughout our Methods section. We have now also added text on our calibration process in lines 191-193, as iterated in the response above.

**-Provide details of the validation process and the different tests conducted to validate the model.**

Thank you for this point. We performed a series of validation tests to our model, following recommendations from Sterman, 2000. An overview of the tests conducted is provided in the “Model testing” section of our Methods, lines 313-324. Moreover, an overview of the main findings of our validation process is provided in the Results, lines 408-419. Finally, further details on validation processes and findings, including both quantitative and/or graphic findings, as well as qualitative assessments and conclusions, are presented in the Supplementary Material. Below, we present a list of the validation tests we conducted and their corresponding Supplementary Files:

1. Behaviour reproduction: S3 Text.
2. Dimensional consistency and parameter assessment: S4 Text.
3. Sensitivity analysis: S5 Text.
4. Extreme conditions: S6 Text.
5. Integration methods and time steps: S7 Text .

**-What is the source of the parameters values for the scenarios. What is the rationale for the selection of the simulation period.**

Effect estimates for scenario impacts were obtained from existing literature based on the geographical focus and study design, prioritised as described in lines 257-262, with the exact values and their sources presented in S3 Table.

Lines 261-266: “… we estimated the impact of different policy scenarios on stunting and overweight using effect estimates derived from previous studies (S3 Table), prioritising primary studies from Peru. When these were not available, we used meta-analyses of Latin American studies, meta-analyses of LMIC-based studies, primary studies from Latin American countries, meta-analyses of high-income countries studies, and primary studies from high-income countries, in this order of priority.”

The magnitude of the scenarios was endogenous, estimated based on modelled taxation revenue. We have revised the corresponding text in lines 246-252 to add clarity:

“Estimated *tax* revenue was distributed to each of the four revenue allocation *scenarios*. For Juntos coverage expansions (allocation scenarios A and C), the poorer quintile had priority over wealthier quintiles. After achieving full coverage at 100%, excess revenue was left unspent. *For Juntos cash amount increase (allocation scenario B), we estimated the cash increase that the accumulated tax revenue could achieve per household per year.* For fruit and vegetable subsidies (allocation scenario D), we calculated the flat, *population-wide* subsidy rate that estimated SSB/UPF tax revenue could fund, using annual fruit and vegetable sales data from Euromonitor[45]”

We chose a 40-year simulation period to achieve a balance between capturing key intergenerational effects of the double burden of malnutrition, an important contribution of our work, while avoiding making highly uncertain long-term assumptions about policy delays that are not supported by existing evidence. We have now added text to clarify this choice in our main manuscript, lines 201-202: *“This time horizon allowed capturing intergenerational impacts of DBM, while also utilising existing shorter-term evidence to support our assumptions”*

**-Discuss the findings with respect to the CLD. Which feedback processes have the highest influence on the system.**

We have now updated the first paragraph of the Discussion to more clearly present our findings from a feedback perspective, referring back to our CLD:

Lines 424-426: Estimated benefits of policy scenarios on overweight in both socioeconomic groups were mainly attributed to SSB and UPF taxes, *despite reinforcing market feedback mechanisms reducing their effectiveness over time (S1 Figure, loop B: SSB market and loop B: UPF market).*

Lines 426-431: Using tax revenue to raise the amount of conditional cash transfers for current recipients of the Juntos Basic Transfer appears particularly effective in reducing stunting among the poorest, where stunting prevalence is highest, *dominating the impact of the reinforcing feedback loop driving the intergenerational transmission of stunting (S1 Figure, loop R: Intergenerational transmission of stunting)*.

Although we did not explicitly perform loop dominance analysis in this study, these results indicate the greater influence of market feedback mechanisms over feedback mechanisms associated with the intergenerational transmission of overweight and stunting in this analysis.

**-What are the main implications of the findings for academia and policy design.**

Our paragraph in lines 475-505 discusses the main implications of our findings for policy design of fiscal double-duty policies. We have now revised the first sentence of the paragraph to more clearly signpost this, in line 475: “Our findings *highlight* *several policy design priorities* for retrofitting fiscal policies in Peru to achieve double-duty objectives.”

We have now also added a sentence to our conclusion further commenting on the academic implications of our work, with a focus on the use of system dynamics, in lines 509-512: “*Our findings demonstrate how system dynamics approaches can advance academic understanding of the DBM by capturing intergenerational pathways, and accounting for over‑time effects such as revenue accumulation and market adaptation, elements largely overlooked in previous modelling studies.”*

**General editorial requests:**

**(Note: not all will apply to your paper, but please check each item carefully)**

***	We ask every co-author listed on the manuscript to fill in a contributing author statement, making sure to declare all competing interests. If any of the co-authors have not filled in the statement, we will remind them to do so when the paper is revised. If all statements are not completed in a timely fashion this could hold up the re-review process. If new competing interests are declared later in the revision process, this may also hold up the submission. Should there be a problem getting one of your co-authors to fill in a statement we will be in contact. Please do not add or remove authors without first discussing this with the handling editor. You can see our competing interests policy here:** [**http://journals.plos.org/plosmedicine/s/competing-interests**](http://journals.plos.org/plosmedicine/s/competing-interests)**.**

***	Please upload any figures associated with your paper as individual TIF or EPS files with 300dpi resolution at resubmission; please read our figure guidelines for more information on our requirements:** [**http://journals.plos.org/plosmedicine/s/figures**](http://journals.plos.org/plosmedicine/s/figures)**. While revising your submission, we strongly recommend that you use PLOS's NAAS tool (**[**https://ngplosjournals.pagemajik.ai/artanalysis**](https://ngplosjournals.pagemajik.ai/artanalysis)**) to test your figure files. NAAS can convert your figure files to the TIFF file type and meet basic requirements (such as print size, resolution), or provide you with a report on issues that do not meet our requirements and that NAAS cannot fix.**

**After uploading your figures to PLOS's NAAS tool -** [**https://ngplosjournals.pagemajik.ai/artanalysis**](https://ngplosjournals.pagemajik.ai/artanalysis)**, NAAS will process the files provided and display the results in the "Uploaded Files" section of the page as the processing is complete.**

**If the uploaded figures meet our requirements (or NAAS is able to fix the files to meet our requirements), the figure will be marked as "fixed" above. If NAAS is unable to fix the files, a red "failed" label will appear above.**

**When NAAS has confirmed that the figure files meet our requirements, please download the file via the download option, and include these NAAS processed figure files when submitting your revised manuscript.**

We have now used NAAS to fix our figures to meet requirements.

***	Please ensure that the paper adheres to the PLOS Data Availability Policy (see** [**http://journals.plos.org/plosmedicine/s/data-availability**](http://journals.plos.org/plosmedicine/s/data-availability)**), which requires that all data underlying the study's findings be provided in a repository or as Supporting Information. For data residing with a third party, authors are required to provide instructions with contact information (web or email address) for obtaining the data. Please note that a study author cannot be the contact person for the data. PLOS journals do not allow statements supported by "data not shown" or "unpublished results." For such statements, authors must provide supporting data or cite public sources that include it.**

The model used in this paper is freely available to download from the Open Science Foundation (OSF) depository following this link: <https://osf.io/pxb9w/>. All data needed to run the model are available both in the Supporting Information of this paper and in the OSF repository, loaded in the model.

***	We expect all researchers with submissions to PLOS in which author-generated code underpins the findings in the manuscript to make all author-generated code available without restrictions upon publication of the work. In cases where code is central to the manuscript, we may require the code to be made available as a condition of publication. Authors are responsible for ensuring that the code is reusable and well documented. Please make any custom code available, either as part of your data deposition or as a supplementary file. Please add a sentence to your data availability statement regarding any code used in the study, e.g. "The code used in the analysis is available from Github [URL] and archived in Zenodo [DOI link]" Please review our guidelines at** [**https://journals.plos.org/plosmedicine/s/materials-software-and-code-sharing**](https://journals.plos.org/plosmedicine/s/materials-software-and-code-sharing) **and ensure that your code is shared in a way that follows best practice and facilitates reproducibility and reuse. Because Github depositions can be readily changed or deleted, we encourage you to make a permanent DOI'd copy (e.g. in Zenodo) and provide the URL.**

The model used in this paper is freely available to download from the Open Science Foundation (OSF) depository following this link: <https://osf.io/pxb9w/> with the following permanent DOI: <https://doi.org/10.17605/OSF.IO/PXB9W>

**FORMATTING - GENERAL**

***	Abstract: Please structure your abstract using the PLOS Medicine headings (Background, Methods and Findings, Conclusions). Please combine the Methods and Findings sections into one section.**

We have now updated the Abstract to combine Methods and Findings into one section, as requested.

***	At this stage, we ask that you include a short, non-technical Author Summary of your research to make findings accessible to a wide audience that includes both scientists and non-scientists. The Author Summary should immediately follow the Abstract in your revised manuscript. This text is subject to editorial change and should be distinct from the scientific abstract. Ideally each sub-heading should contain 2-3 single sentence, concise bullet points containing the most salient points from your study. In the final bullet point of 'What Do These Findings Mean?', please include the main limitations of the study in non-technical language. Please see our author guidelines for more information:** [**https://journals.plos.org/plosmedicine/s/revising-your-manuscript#loc-author-summary**](https://journals.plos.org/plosmedicine/s/revising-your-manuscript#loc-author-summary)**.**

We have now added the following non-technical Author Summary to our paper, on pages 2-3:

*Why Was This Study Done?*

- *Peru faces a double burden of malnutrition, with high levels of both overweight and stunting.*
- *The World Health Organization has called for double-duty actions to address the double-burden of malnutrition, including by retrofitting existing interventions to concurrently address multiple forms of malnutrition.*
- *The World Health Organization recommends fiscal policies as an effective way to improve diets, but their potential as double-duty actions has not been previously explored.*

*What Did the Researchers Do and Find?*

- *We developed a system dynamics model that simulates overweight and stunting trends in children and adults of reproductive age in Peru between 2000 and 2040 across two socioeconomic groups.*
- *We developed policy scenarios informed by two of Peru’s flagship fiscal policies with strong potential to reduce overweight and stunting, its sugar-sweetened beverage tax and its conditional cash transfer Juntos, as well as existing fiscal policy recommendations for healthier diets.*
- *We modelled a total of eight scenarios combining two tax options on unhealthy foods and beverages with four ways of reallocating the resulting revenue by expanding or increasing Juntos transfers, expanding the Juntos Early Childhood Transfer, or funding a fruit and vegetable subsidy.*
- *Retrofitting Peru’s current fiscal policies by expanding its current sugar sweetened beverage tax and reinvesting its revenue to increase the amount of cash offered through Peru’s existing conditional cash transfer programme has strong potential to reduce total rates of overweight and stunting among the poorest by 2040.*

*What Do These Findings Mean?*

- *Retrofitting fiscal policies to incorporate double-duty objectives by reallocating revenues from unhealthy food and beverage taxes towards social safety nets holds significant promise for addressing the double burden of malnutrition in Peru*
- *These benefits are maximised when revenues are strategically reinvested in programmes targeting the poorest.*
- *Future modelling studies should examine the health impacts and cost‑effectiveness of reallocating revenues from unhealthy food and beverage taxes across different strategies to address the diverse forms of the double burden of malnutrition.*

***	Please express the main results with 95% CIs as well as p values. When reporting p values please report as p<0.001 and where higher as the exact p value p=0.002, for example. Throughout, suggest reporting statistical information as follows to improve clarity for the reader "22% (95% CI [13%,28%]; p</=)". Please be sure to define all numerical values at first use.**

Not applicable. Our uncertainty intervals derive from probabilistic sensitivity analyses, not hypothesis testing, thus no p-values are produced.

***	Please include page numbers and line numbers in the manuscript file. Use continuous line numbers (do not restart the numbering on each page).**

Page numbers and continuous line numbers are included.

***	Please cite the reference numbers in square brackets. Citations should precede punctuation.**

Reference numbers are now presented in square brackets, preceding punctuation.

**FIGURES AND TABLES**

***	Please provide titles and legends for all figures and tables (including those in Supporting Information files).**

All figures and tables have titles and legends.

***	Please define all abbreviations used in each figure/table (including those in Supporting Information files).**

All abbreviations are defined.

***	Please consider avoiding the use of red and green in order to make your figure more accessible to those with color blindness.**

We have checked our graph colours using R package “colorblindcheck” and ensured that all colours are distinguishable for those with colour blindness.

**SUPPLEMENTARY MATERIAL**

***	Please note that supplementary material will be posted as supplied by the authors. Therefore, please amend it according to the relevant comments outlined here.**

***	Please cite your Supporting Information as outlined here:** [**https://journals.plos.org/plosmedicine/s/supporting-information**](https://journals.plos.org/plosmedicine/s/supporting-information)

We have updated our Supplementary material and relevant text citations as per recommendations

**REFERENCES**

***	PLOS uses the numbered citation (citation-sequence) method and first six authors, et al. *	Please ensure that journal name abbreviations match those found in the National Center for Biotechnology Information (NCBI) databases (**[**http://www.ncbi.nlm.nih.gov/nlmcatalog/journals**](http://www.ncbi.nlm.nih.gov/nlmcatalog/journals)**), and are appropriately formatted and capitalised.**

We have now updated our citation style as per PLOS method.

***	Where website addresses are cited, please include the complete URL and specify the date of access (e.g. [accessed: 12/06/2023]).**

URLs and dates of access are presented

***	Please also see** [**https://journals.plos.org/plosmedicine/s/submission-guidelines#loc-references**](https://journals.plos.org/plosmedicine/s/submission-guidelines#loc-references) **for further details on reference formatting.**

We now use PLOS reference style.

**STUDY TYPE-SPECIFIC REQUESTS**

**The following list is derived from Geoffrey P Garnett, Simon Cousens, Timothy B Hallett, Richard Steketee, Neff Walker. Mathematical models in the evaluation of health programmes. (2011) Lancet DOI:10.1016/S0140-6736(10)61505-X:**

***	If pertinent, please provide a diagram that shows the model structure, including how the natural history of the disease is represented, the process and determinants of disease acquisition, and how the putative intervention could affect the system.**

We have now presented a summarised model structure in the form of a causal loop diagram in S1 Figure, in addition to our model structure description in S1 Text.

***	Please provide a complete list of model parameters, including clear and precise descriptions of the meaning of each parameter, together with the values or ranges for each, with justification or the primary source cited and important caveats about the use of these values noted.**

All model parameters with corresponding values, descriptions, and sources, were relevant are presented in the Supplementary Appendix (S2 Text, S1 Table, S3 Table, S4 Text)

***	Please provide a clear statement about how the model was fitted to the data, including goodness-of-fit measure, the numerical algorithm used, which parameter varied, constraints imposed on parameter values, and starting conditions.**

Details on model calibration are now presented in lines 186-189 of the revised manuscript. Parameter values and stock starting conditions are presented in S3 Table and S4 Table. Parameters varied as part of probabilistic sensitivity analysis are presented in S4 Table.

***	For uncertainty analyses, please state the sources of uncertainties quantified and not quantified [can include parameter, data, and model structure].**

Sources of uncertainty are presented in S4 Table.

***	Please provide sensitivity analyses to identify which parameter values are most important in the model. Uncertainty estimates seek to derive a range of credible results on the basis of an exploration of the range of reasonable parameter values. The choice of method should be presented and justified.**

Sensitivity analyses are presented in S5 Text. Figures 1 and 2 in S5 Text provide Tornado graphs showing which parameters were most important to the model

***	Please discuss the scientific rationale for the choice of model structure and identify points where this choice could influence conclusions drawn. Please also describe the strength of the scientific basis underlying the key model assumptions.**

Rationale for the choice of the model approach and structure are provided in lines 168-178 of the revised manuscript with further details about model structure presented in S1 Text. Choices around model assumptions underlying model equations and structure are presented when relevant in the Comments column in S4 Text.

***	For studies that develop a prediction model or evaluate its performance, please ensure that the study is reported according to the TRIPOD statement (**[**https://www.equator-network.org/reporting-guidelines/tripod-statement**](https://www.equator-network.org/reporting-guidelines/tripod-statement)**) and include the completed checklist as Supporting Information. Please add the following statement, or similar, to the Methods: "This study is reported as per the Transparent Reporting of a Multivariable Prediction Model for Individual Prognosis Or Diagnosis (TRIPOD) statement (S1 Checklist)." For studies using machine learning, please use the TRIPOD-AI checklist. When completing the checklist, please use section and paragraph numbers, rather than page numbers.**

Not Applicable
