# Supplementary material for: Retrofitting fiscal policies to reduce the double burden of malnutrition in Peru: A system dynamics modelling study
Source: PLoS Med. 2026 Aug 13;23(8):e1005167. doi: 10.1371/journal.pmed.1005167 (PMC13472416; doi:10.1371/journal.pmed.1005167)
Supplement: Supplementary file 17 [file pmed.1005167.s019.docx]

Thank you to the peer reviewers for their positive feedback and to the academic editor and statistical reviewer for the additional comments. We provide below a point-by-point response to all comments and present the relevant text from the manuscript, highlighting newly added text in italics.

**1)	I am not sure why they could not provide a clearer answer to the questions on cost-effectiveness using DALYs and also what mix of "double duty" interventions were considered (and the evidence of effectiveness thereof).**

Our model includes children and adults up to the age of 49, as Peru does not have data on weight and height for older people. As a result, we are only able to provide DALYs for people under 49. However, this will result in an underestimation of DALYs attributed to overweight. Evidence from the GBD study indicates that high BMI-attributable DALYs increase with age and are highest between 55 and 64 years^[1]^. On the other hand, stunting-attributable DALYs mainly occur in children under-5. To avoid systematically underestimating the benefits of overnutrition-related interventions and providing misleading comparisons between undernutrition and overnutrition policies, we decided to not include DALYs in the model. We have now updated the relevant limitation in the discussion for further clarity:

Lines 462-465: “Finally, we did not conduct cost-effectiveness analysis, as summary measures of population health needed for this analysis, *such as disability-adjusted life years,* would have been underestimated in this model due to its age group coverage, *which does not include people over 49, excluding age groups where overweight-attributable measures are the highest.*”

The mix of double-duty interventions modelled in this paper is presented in Figure 1 of the manuscript. Briefly, we simulated eight combined policy scenarios, resulting from the combinations of two revenue generation scenarios (current SSB tax in Peru and extended SSB+UPF tax) and four revenue allocation scenarios (three scenarios expanding existing cash assistance programs in Peru and a fruit and vegetable subsidy scenario). These were informed by WHO recommendations on fiscal policies for healthier diets and the existing policy landscape in Peru. We expand on this in our Method’s section “Policy selection”, page 6 of the manuscript.

The evidence we used to model the impact of each scenario is described in detail in section “Modelling policy impacts”, pages 7-8 of the manuscript. We also provide a detailed overview of effect estimates in the Supporting Information (Tables S2 and S3). Briefly, we modelled the impact of unhealthy food taxes on overweight using published estimates of own-price and cross-price elasticities for UPF and SSB demand and estimates linking country-level changes in SSB consumption to overweight prevalence from a multi-country study. We modelled the effects of Juntos on stunting and overweight in children using data from primary studies from Peru and Mexico. Finally, we estimated the impacts of a fruit and vegetable subsidy on stunting and overweight using data from a multi-country study linking food prices to national stunting prevalence across 176 countries, and a longitudinal study from the U.S., which estimated the impact of a 10% price increase in fruit and vegetables on overweight. Please, see the relevant sections in the manuscript for further details.

**2)	From my perspective, it is a bit naive to assume that revenues from sin taxes will only go to one sector, especially when interventions that address stunting are largely multisectoral (as shown in Ref 22). What isolated interventions for stunting reduction were modeled? Was breastfeeding considered a double duty intervention?**

**Statistical reviewer: Would it be possible to include reduced diarrheal disease burden as a double-duty intervention?**

This paper aims to investigate the potential of fiscal policies as double-duty actions to address the double burden of malnutrition. This focus on fiscal policy is motivated by WHO recommendations highlighting fiscal measures as a promising lever for improving diets and nutrition outcomes^[2]^, and by the lack of explicit modelling studies examining their dual impact on undernutrition and overweight^[3]^, as outlined in our Introduction. By focusing on fiscal policies, our study addresses this specific gap in the literature.

Within this scope, we model one key fiscal policy mechanism for stunting reduction in Peru: the Juntos conditional cash transfer programme. This programme has been widely recognised as an important contributor to previous reductions in stunting in the country^[4]^, making it a policy-relevant and empirically grounded case for scenario analysis. We agree that other interventions, such as breastfeeding promotion or interventions targeting diarrhoeal disease, are relevant alternatives of double-duty actions. However, these lie outside the focus of fiscal policy instruments examined in this paper.

We have now clarified this scope more explicitly in the manuscript lines 148-153: “No prior studies have explicitly examined the potential of retrofitting existing nutrition‑relevant *fiscal* policies, such as Peru’s SSB tax and Juntos, to jointly target the DBM. In this study, we investigated the *role of fiscal policies on the DBM in Peru* *by modelling the* potential impact of retrofitting unhealthy food and beverage taxation for double-duty objectives through revenue reallocation, expanding conditional cash transfer programmes and fruit and vegetable subsidies in Peru.”

We have also expanded the Discussion to highlight the importance of future work exploring complementary non-fiscal interventions, including breastfeeding promotion and interventions to reduce infectious disease burden, lines 455-459: “Fourth, our study does not model all potential double-duty actions in Peru but rather focuses on flagship fiscal policies that have strong DBM potential due to existing evidence of their impacts on nutrition outcomes[24, 26-32]. *Future research should explore the impact of other policy options with strong double-duty potential, including breastfeeding promotion and healthcare interventions targeting infectious disease burden.”*

Finally, we appreciate the reviewer’s concern regarding the assumption that revenues from sin taxes are allocated to a single sector. While there are examples where the entirety of revenues is directed towards specific purposes (e.g. Colombia’s tobacco tax revenue for health insurance, Guatemala’s tobacco tax revenue for health programmes, and alcohol tax revenues in Gabon for the development of sport)^[5]^, most tax revenues reallocation is partial and distributed across sectors. In our model, we adopt a simplifying assumption whereby the total revenue is allocated to Juntos or subsidy interventions in order to estimate the maximum potential impact of such fiscal policies under an idealised scenario, rather than to reflect typical policy implementation. Importantly, our results indicate that many of the simulated policy effects could be achieved with only a fraction of the total revenue generated, which we believe strengthens the policy relevance of our findings.

We have now clarified this intend in our Methods, lines 235-236: “Estimated tax revenue was distributed to each of the four revenue allocation scenarios *to estimate the maximum potential impact under an idealised full earmarking assumption.”*

**3)	Finally, do they have any data on who is consuming SSBs and ultra-processed foods in Peru at the moment? Will the additional cash in Juntos 2 not go towards some of these items? What is the evidence that Juntos 1 has led to behavior change favoring healthy diets among the poor?""**

**Statistical reviewer: there was no consideration of a counterfactual scenario in which Juntos did not exist, which would need to be done to answer this question.**

Peru currently lacks sufficiently detailed and nationally representative dietary data to characterise consumption of SSBs and ultra-processed foods by population subgroup. As a result, we modelled population-wide impacts using available data on sales. We have now clarified this in our Methods, line 264-265: “*Due to lack of relevant data, we assumed uniform SSB and UPF consumption across income groups.*”

We agree that an increase in household income through programmes such as Juntos could potentially lead to increased consumption of unhealthy foods. However, existing evidence on the impact of Juntos on overweight and stunting has not examined the mechanisms underlying these associations. Potential pathways may include direct effects on dietary intake through changes in household income, as well as impacts due to conditionalities, such as healthcare visits that include nutritional counselling. Due to the lack of data on these pathways, we modelled the impact of Juntos on overweight and stunting using available effect estimates, without explicitly modelling changes in dietary behaviour attributed to Juntos. However, we have now clarified in our Discussion that changes in conditional cash transfer programmes, like Juntos, should be accompanied by wider food policy interventions to prevent extra cash being used towards unhealthy food intake, lines 483-487: “Behavioural change initiatives, such as school-based programmes promoting healthy habits, as well as food environment modifications, could reinforce and amplify the benefits of *tax and subsidy* measures, ensuring a more lasting impact on malnutrition outcomes[66], *whilst enhancing the impact of conditional cash transfers by discouraging spending on unhealthy foods*.”

We appreciate the statistical reviewer’s suggestion for a counterfactual scenario in which Juntos does not exist as a way to explore the impact of current Juntos policies on diets. However, given that Juntos has been implemented in Peru since 2005 and the lack of evidence exploring the pathways of its impacts described above, we believe that modelling such a scenario will add little value to current understanding of Juntos implications on the double burden of malnutrition.

**GENERAL EDITORIAL REQUESTS:**

*** Please confirm that your title complies with PLOS Medicine's style. Your title must be nondeclarative and not a question. It should begin with main concept if possible. "Effect of" should be used only if causality can be inferred, i.e., for an RCT. Please place the study design ("A randomized controlled trial," "A retrospective study," "A modelling study," etc.) in the subtitle (ie, after a colon).**

We confirm that our title complies with PLOS Medicine’s style, as it not declarative, begins with main policy concept, includes main outcome, and clarifies study design: “Retrofitting fiscal policies to reduce the double burden of malnutrition in Peru: a system dynamics modelling study”

*** Please ensure that the Introduction ends with a clear description of the study question or hypothesis.**

The introduction ends with a clear description of the study question as follows: “In this study, we investigated the role of fiscal policies on the DBM in Peru by modelling the potential impact of retrofitting unhealthy food and beverage taxation for double-duty objectives through revenue reallocation, expanding conditional cash transfer programmes and fruit and vegetable subsidies in Peru. We employed a system dynamics approach to estimate impacts of various tax and reallocation scenarios on national stunting and overweight across socioeconomic groups.”

*** Please ensure that all abbreviations are defined at first use throughout the text.**

We can confirm that all abbreviations are defined at first use throughout the text.

*** Please confirm that all numbers presented in the abstract are present and identical to numbers presented in the main manuscript text.**

Thanks for this comment. We have now updated the abstract and results so that the presented results are identical in both sections.

*** Please disclose any use of AI tools and technologies to the study or article content in the Methods, including the name(s) of any tool(s) used and a description of their use. Please see our guidelines for more information:** [**https://journals.plos.org/plosmedicine/s/ethical-publishing-practice#loc-artificial-intelligence-tools-and-technologies**](https://journals.plos.org/plosmedicine/s/ethical-publishing-practice#loc-artificial-intelligence-tools-and-technologies)**.**

AI tools were not used to generate any article content and were only used for minor editorial support (grammar, syntax, and clarity checks) in limited, selected parts of the text.

*** Statistical reporting: Please revise throughout the manuscript, including tables and figures. We appreciate that you do not have p-values, and it is fine to leave these out. However, please ensure that otherwise you adhere to the following style rules, including a comma instead of a hyphen between the 95% CI.**

**- Please report statistical information as follows to improve clarity for the reader ""22% (95% CI [13,28]; p</=)"".**

**- Please separate upper and lower bounds with commas instead of hyphens as the latter can be confused with reporting of negative values.**

**- Please repeat statistical definitions (HR, CI etc.) for each set of parentheses.**

We have now updated our statistical reporting according to recommendations above.

*** Financial statement: please include URLs to the funders websites.**

We have now added URLs for funders supporting authors of the study in the acknowledgements section

*** Data availability: thank you for including this statement. In addition to the OSF link, can you also provide us with a DOI for the data?**

We added the OSF doi in the data availability statement.

**ABSTRACT**

*** Please confirm that your abstract complies with our requirements, including format (three sections: Background, Methods and Findings, and Conclusions) and providing all the information relevant to this study type** [**https://journals.plos.org/plosmedicine/s/submission-guidelines#loc-abstract**](https://journals.plos.org/plosmedicine/s/submission-guidelines#loc-abstract)

Thank you. We can confirm that the abstract now complies with all requirements.

*** In the last sentence of the Abstract Methods and Findings section, please describe the main limitation(s) of the study’s methodology.**

We have added a sentence outlining the main limitations of the study: “This study relies on several assumptions regarding policy impacts and responses and does not incorporate cost-effectiveness analysis due to its exclusion of older age groups.”

**AUTHOR SUMMARY**

*** In the author summary, in the final bullet point of 'What Do These Findings Mean?', please include the main limitations of the study in non-technical language.**

We have added a bullet point outlining the main limitations of the study: “-	This study relies on several assumptions regarding policy impacts, market growth, and industry responses and does not incorporate cost-effectiveness analysis due to its exclusion of older age groups”.

**FIGURES AND TABLES**

*** Please define all abbreviations used in each figure/table (including those in Supporting Information files). (including SSB and UPF).**

We have now defined all abbreviations in figures and tables on main manuscript and Supporting Information.

*** Please ensure there is enough description of each of the figures in the legend. This includes describing what the grey areas and error bars mean (Figure 2 and Figure 4).**

We have now added further clarification in the description of Figures 2 and 4.

1. Zhou XD, Chen QF, Yang W, et al. Burden of disease attributable to high body mass index: an analysis of data from the Global Burden of Disease Study 2021. EClinicalMedicine 2024; 76: 102848.
2. WHO. Fiscal policies to promote healthy diets - WHO guideline. 2024.
3. Hawkes C, Ruel MT, Salm L, Sinclair B, Branca F. Double-duty actions: seizing programme and policy opportunities to address malnutrition in all its forms. The Lancet. 2020;395(10218):142-55.
4. Andersen CT, Reynolds SA, Behrman JR, Crookston BT, Dearden KA, Escobal J, et al. Participation in the Juntos Conditional Cash Transfer Program in Peru Is Associated with Changes in Child Anthropometric Status but Not Language Development or School Achievement. J Nutr. 2015;145(10):2396-405.

   Perez-Lu JE, Carcamo C, Nandi A, Kaufman JS. Health effects of 'Juntos', a conditional cash transfer programme in Peru. Matern Child Nutr. 2017;13(3):07
5. <https://hpfhub.info/using-health-promotion-funding/>
