# Supplementary material for: Retrofitting fiscal policies to reduce the double burden of malnutrition in Peru: A system dynamics modelling study
Source: PLoS Med. 2026 Aug 13;23(8):e1005167. doi: 10.1371/journal.pmed.1005167 (PMC13472416; doi:10.1371/journal.pmed.1005167)
Supplement: Peer review history

# Editor report: Decision Letter 0

**DOI:** 10.1371/journal.pmed.1005167.r001

**Authors:** Andreia Cunha

4 Dec 2025

Dear Dr Seferidi,

Thank you for submitting your manuscript entitled "Retrofitting fiscal policies to reduce the double burden of malnutrition in Peru: a system dynamics modelling study" for consideration by PLOS Medicine.

Your manuscript has now been evaluated by the PLOS Medicine editorial staff, and I am writing to let you know that we would like to send your submission out for external peer review.

However, before we can send your manuscript to reviewers, we need you to complete your submission by providing the metadata that is required for full assessment. To this end, please login to Editorial Manager where you will find the paper in the 'Submissions Needing Revisions' folder on your homepage. Please click 'Revise Submission' from the Action Links and complete all additional questions in the submission questionnaire.

For clinical studies, please upload a copy of your trial study protocol as a supporting information file. The study protocol should be the version submitted for approval to the institutional review board or ethics committee, should include any amendments to the study protocol, as well as the date of their approval by the institutional review or ethics committee. Please also detail any deviations from the study protocol in the Methods section of your manuscript. The editors will consider the protocol and study conduct prior to a final decision for external review.

Please re-submit your manuscript within two working days, i.e. by Dec 08 2025 11:59PM.

Login to Editorial Manager here: https://www.editorialmanager.com/pmedicine

Once your full submission is complete, your paper will undergo a series of checks in preparation for peer review. Once your manuscript has passed all checks it will be sent out for review.

Feel free to email us at plosmedicine@plos.org if you have any queries relating to your submission.

Kind regards,

Andreia Cunha, PhD

Senior Editor

PLOS Medicine

# Peer review: Decision Letter 1

**DOI:** 10.1371/journal.pmed.1005167.r002

**Authors:** Alexandra Tosun

30 Jan 2026

Dear Dr Seferidi,

Many thanks for submitting your manuscript "Retrofitting fiscal policies to reduce the double burden of malnutrition in Peru: a system dynamics modelling study" (PMEDICINE-D-25-04199R1) to PLOS Medicine. The paper has been reviewed by subject experts and a statistician; their comments are included below and can also be accessed here: [LINK]

As you will see, the reviewers provided mixed feedback on your study. While they are interested in the study, the reviewers also expressed concerns about the lack of detail regarding the modeling and the explanation of the context of the issue addressed by the study. After discussing the paper with the editorial team and an academic editor with relevant expertise, I'm pleased to invite you to revise the paper in response to the reviewers' comments. We plan to send the revised paper to some or all of the original reviewers, and we cannot provide any guarantees at this stage regarding publication.

In addition to these revisions, you may need to complete some formatting changes, which you will receive in a follow up email. A member of our team will be in touch with a set of requests shortly. If you do not receive a separate email within a few days, please assume that checks have been completed, and no additional changes are required.

When you upload your revision, please include a point-by-point response that addresses all of the reviewer and editorial points, indicating the changes made in the manuscript and either an excerpt of the revised text or the location (eg: page and line number) where each change can be found. Please also be sure to check the general editorial comments at the end of this letter and include these in your point-by-point response. When you resubmit your paper, please include a clean version of the paper as the main article file and a version with changes tracked as a marked-up manuscript. It may also be helpful to check the guidelines for revised papers at http://journals.plos.org/plosmedicine/s/revising-your-manuscript for any that apply to your paper.

We ask that you submit your revision by Feb 20 2026. However, if this deadline is not feasible, please contact me by email, and we can discuss a suitable alternative.

Don't hesitate to contact me directly with any questions (atosun@plos.org).

Best regards,

Alexandra

Alexandra Tosun, PhD

Senior Editor

PLOS Medicine

atosun@plos.org

-----------------------------------------------------------

Comments from the reviewers:

Reviewer #1: Thank you for the opportunity to review this interesting and impactful modeling study of redirecting sugar-sweetened beverage and junk food tax revenue toward fruit and vegetable subsidies in Peru. This well-written article addresses an innovative and important topic. Below are some suggestions for improving the rigor, presentation, and interpretation in rough order of priority:

The basic premise of the study seems to be that allocating unhealthy food tax revenue toward healthy food is more impactful than not allocating - and the more is allocated, the better. This is a somewhat self-evident finding and risks drawing an erroneous policy conclusion because it does not consider what the revenue would be spent on otherwise. There is a possibility that unhealthy food tax revenue could instead be allocated to an activity that generates even more population health benefits than, say, subsidizing fruits and vegetables. An example might be immunization against diarrheal diseases, which is extremely cost-effective at reducing morbidity/mortality and can reduce malnutrition/stunting by addressing nutrient malabsorption from diarrhea. Could the authors comment on: (A) what unhealthy food tax revenue is currently spent on, (B) what are the marginal opportunities of Peruvian healthcare (e.g., health services that are not yet funded), (C) what is the cost-effectiveness of spending it on the proposed interventions (in terms of money per QALY gained, DALY averted, etc.), and (D) how this compares to cost-effectiveness benchmarks for Peruvian government health spending?

It is great that the authors conducted both univariate (+/- 10% on individual assumptions) and multivariate (PSA) sensitivity analyses. However, it is critical to then propagate the uncertainty ascertained in multivariate sensitivity analysis to reported outcomes. All reported results in the text and tables should have 95% UIs, and all figures should be graphed with uncertainty bands. Currently, this is implemented inconsistently throughout the manuscript.

Furthermore, there may be internal inconsistencies in when uncertainty is considered vs. not. E.g., in Figure 3, the authors have either neglected to graph confidence bands for some curves, or else the confidence bands are unreasonably tight for some bands so as to be invisible (e.g., adult overweight forecasts for the wealthiest quintile in Scenarios A and B; for the poorest quintile in Scenarios B and C, child stunting forecasts for both quintiles under all scenarios except for Scenario D). The authors should revisit assumptions and PSA design to ensure all main sources of uncertainty are captured, including uncertainty in baselines and their forecasts even in the absence of revenue reallocation.

For the scenario set, the authors made conservative assumptions of no effect in groups where no evidence of an effect exists. While this seems reasonable for a main analysis, it would be apt to vary this assumption in sensitivity analysis.

In Figure 2A, could the authors explain why there is a revenue downturn between 2025 and 2030? What drives this?

Also in Figure 2A, could the authors graph revenue time-series for the other revenue generation scenarios for ease of comparison? It might be nice to show these on a per capita or per child basis to contextualize the ~3Bn magnitude for Peru, and show how things are trending after adjusting for population growth.

Table 1 might be better displayed as a floating bar chart or box and whiskers chart to make it easier to take in, considering values down the columns all have the same units so could be graphed together. Colors and groupings could be used to visually organize the information.

In Supplementary Table S2 showing the list of scenarios, it would be helpful to add columns specifying which age groups the intervention is assumed to impact, and the assumed effect sizes. In their main analysis.

There appears to be a table misnumbering issue in the Appendix. The second Supplementary Table S1 should be S2, S2 should be S3, etc.

Possible typographical error: "We adjusted the impact of Juntos scenarios on overweight and stunting using a an annually varying discount rate" - extra "a."

Reviewer #2: In line 64, define SSBs (as this is the first time they are mentioned in the manuscript).

In line 65, for the sentence 'have enormous potential as double-duty actions', I suggest adding a reference.

Line 66, for the sentence 'These policies, usually implemented in silos', I suggest emphasising why this is a problem in the context of Peru before jumping to the solution. Why are these two potential double duty actions (DDA) chosen? Are DDAs in Peru likely to have the greatest potential impact on the double burden of malnutrition (DCM)? Why study these DDAs and not others? Is the fact that the DDAs studied are implemented in silos the problem? Or is there no policy for the double burden of malnutrition in Peru that brings together government actions with the potential to become DDA? Were the potential DDAs analysed in the article designed to address public problems other than DCM? Is there no evidence of the impact of the potential DDA analysed in this study on the achievement of the results proposed in its creation? Is there no evidence of the impact of the potential DDA analysed in this study on DCM? I suggest better defining and explaining the context of the issue addressed by the study.

In line 73, in the sentence 'Peru has implemented fiscal policies to address undernutrition and overnutrition,' I suggest adding "separately" or 'in silos' at the end.

I suggest dividing or rewriting the paragraph contained between lines 73-85. The combination of both potential DDAs reads awkwardly. Both government actions were created with specific and 'different' objectives (Juntos: 'to reduce intergenerational poverty in Peru'; SSB taxes in Peru: to protect public health by discouraging the consumption of these products due to their associated harms... It would be good to highlight these in the text to guide the order of the paragraphs.

Line 86 Add reference or data (prevalence) for the sentence 'Despite Peru's high DBM prevalence'; I suggest that they be included in the first paragraph of the introduction.

The statement on lines 86-87 "policies targeting undernutrition and overnutrition appear to be designed and implemented separately, limiting their potential for synergistic impact" . Currently, SSB 87 tax revenue is not redirected to improve access to healthier diets or reduce poverty, a major driver of poor diets, and the opportunity to achieve double-duty benefits remains underutilized". I suggest that this be moved up to paragraph 2 and that the issue addressed by the study be better explained before moving on to the solutions.

Lines 92-94 mention a "systems dynamics approach"; it would be ideal to add a brief sentence on why this approach is appropriate for addressing the problem identified in the study.

Line 97: I suggest expanding on the explanation of why the system dynamics model (and not another) is appropriate for this study.

In the section on policy selection and in the introduction, it is not clear why the two potential DDAs were chosen (and not other DDAs suggested by the WHO).

In lines 115-116, it is suggested to clarify the reference point for the second scenario, both the 'SSB tax increase' and the 'new 10% UPF tax'. Do both follow the Colombian model as a reference for this scenario? Add a reference to what Colombia applies.

In lines 118-121, it is suggested to explain which criteria are used to establish scenarios A, B, C, and D.

In lines 125-138, in scenario D, it is suggested to specify the target audience for the fruit and vegetable subsidy. Are they the beneficiaries of the JUNTOS programme? The general population? Why was a subsidy for fruit and vegetables chosen, and foods rich in iron and essential amino acids? The districts that receive the JUNTOS programme have the highest rates of IDA and iron deficiency anaemia in the region. Among many factors, one is the poor quality of the diet in terms of the aforementioned nutrients.

In lines 139-151, it is suggested to explain why IMCI and overweight were considered, and why the impact on iron deficiency anaemia (part of the double burden of malnutrition) was not considered, as this is one of the main public health problems in Peru today.

In lines 144-146, the assumption made is debatable. Officials from the Peruvian Ministry of Economy often argue that there is no evidence to demonstrate the impact of existing taxes on SBS, which is why there is no evidence to support their increase. Furthermore, they mention that the increase in SBS consumption and the increase in SP and OB in Peru demonstrate poor impact.

In lines 232-243, it is suggested that the target audience for scenario D be specified.

In lines 335-355, it is suggested that the policy recommendations be framed and aligned with current country policies in Peru, such as the National Multisectoral Health Policy to 2030 and the Peru National Development Strategic Plan to 2050. It is also suggested that consideration be given to the potential barriers and facilitators to the implementation of the suggested recommendations.

Reviewer #3: The study is clearly important, but the paper has very serious weaknesses especially with regard to the application of the System Dynamics (SD) approach. The paper needs to be improved in line with the following:

-Clarify the contribution of the paper and in which academic literature it is positioned. What is the novelty of the study.

-Include a literature review section including a review of the use of SD in the context of malnutrition and related food policy.

-Provide the rationale for using SD and the justification for the context of the study.

-Include a qualitative mapping of the system in the form of the a Causal Loop Diagram (CLD).

-Include a description of the model building process including the main stocks and flows and the rate equations.

-Provide more details about the data collection process, data sources, and how the model was calibrated.

-Provide details of the validation process and the different tests conducted to validate the model.

-What is the source of the parameters values for the scenarios. What is the rationale for the selection of the simulation period.

-Discuss the findings with respect to the CLD. Which feedback processes have the highest influence on the system.

-What are the main implications of the findings for academia and policy design.

--------------------------------------------------------- ---

General editorial requests:

(Note: not all will apply to your paper, but please check each item carefully)

*We ask every co-author listed on the manuscript to fill in a contributing author statement, making sure to declare all competing interests. If any of the co-authors have not filled in the statement, we will remind them to do so when the paper is revised. If all statements are not completed in a timely fashion this could hold up the re-review process. If new competing interests are declared later in the revision process, this may also hold up the submission. Should there be a problem getting one of your co-authors to fill in a statement we will be in contact. Please do not add or remove authors without first discussing this with the handling editor. You can see our competing interests policy here: http://journals.plos.org/plosmedicine/s/competing-interests.

*Please upload any figures associated with your paper as individual TIF or EPS files with 300dpi resolution at resubmission; please read our figure guidelines for more information on our requirements: http://journals.plos.org/plosmedicine/s/figures. While revising your submission, we strongly recommend that you use PLOS's NAAS tool (https://ngplosjournals.pagemajik.ai/artanalysis) to test your figure files. NAAS can convert your figure files to the TIFF file type and meet basic requirements (such as print size, resolution), or provide you with a report on issues that do not meet our requirements and that NAAS cannot fix.

After uploading your figures to PLOS's NAAS tool - https://ngplosjournals.pagemajik.ai/artanalysis, NAAS will process the files provided and display the results in the "Uploaded Files" section of the page as the processing is complete.

If the uploaded figures meet our requirements (or NAAS is able to fix the files to meet our requirements), the figure will be marked as "fixed" above. If NAAS is unable to fix the files, a red "failed" label will appear above.

When NAAS has confirmed that the figure files meet our requirements, please download the file via the download option, and include these NAAS processed figure files when submitting your revised manuscript.

*Please ensure that the paper adheres to the PLOS Data Availability Policy (see http://journals.plos.org/plosmedicine/s/data-availability), which requires that all data underlying the study's findings be provided in a repository or as Supporting Information. For data residing with a third party, authors are required to provide instructions with contact information (web or email address) for obtaining the data. Please note that a study author cannot be the contact person for the data. PLOS journals do not allow statements supported by "data not shown" or "unpublished results." For such statements, authors must provide supporting data or cite public sources that include it.

*We expect all researchers with submissions to PLOS in which author-generated code underpins the findings in the manuscript to make all author-generated code available without restrictions upon publication of the work. In cases where code is central to the manuscript, we may require the code to be made available as a condition of publication. Authors are responsible for ensuring that the code is reusable and well documented. Please make any custom code available, either as part of your data deposition or as a supplementary file. Please add a sentence to your data availability statement regarding any code used in the study, e.g. "The code used in the analysis is available from Github [URL] and archived in Zenodo [DOI link]" Please review our guidelines at https://journals.plos.org/plosmedicine/s/materials-software-and-code-sharing and ensure that your code is shared in a way that follows best practice and facilitates reproducibility and reuse. Because Github depositions can be readily changed or deleted, we encourage you to make a permanent DOI'd copy (e.g. in Zenodo) and provide the URL.

FORMATTING - GENERAL

*Abstract: Please structure your abstract using the PLOS Medicine headings (Background, Methods and Findings, Conclusions). Please combine the Methods and Findings sections into one section.

*At this stage, we ask that you include a short, non-technical Author Summary of your research to make findings accessible to a wide audience that includes both scientists and non-scientists. The Author Summary should immediately follow the Abstract in your revised manuscript. This text is subject to editorial change and should be distinct from the scientific abstract. Ideally each sub-heading should contain 2-3 single sentence, concise bullet points containing the most salient points from your study. In the final bullet point of 'What Do These Findings Mean?', please include the main limitations of the study in non-technical language. Please see our author guidelines for more information: https://journals.plos.org/plosmedicine/s/revising-your-manuscript#loc-author-summary.

*Please express the main results with 95% CIs as well as p values. When reporting p values please report as p<0.001 and where higher as the exact p value p=0.002, for example. Throughout, suggest reporting statistical information as follows to improve clarity for the reader "22% (95% CI [13%,28%]; p</=)". Please be sure to define all numerical values at first use.

*Please include page numbers and line numbers in the manuscript file. Use continuous line numbers (do not restart the numbering on each page).

*Please cite the reference numbers in square brackets. Citations should precede punctuation.

FIGURES AND TABLES

*Please provide titles and legends for all figures and tables (including those in Supporting Information files).

*Please define all abbreviations used in each figure/table (including those in Supporting Information files).

*Please consider avoiding the use of red and green in order to make your figure more accessible to those with color blindness.

SUPPLEMENTARY MATERIAL

*Please note that supplementary material will be posted as supplied by the authors. Therefore, please amend it according to the relevant comments outlined here.

*Please cite your Supporting Information as outlined here: https://journals.plos.org/plosmedicine/s/supporting-information

REFERENCES

*PLOS uses the numbered citation (citation-sequence) method and first six authors, et al.

*Please ensure that journal name abbreviations match those found in the National Center for Biotechnology Information (NCBI) databases (http://www.ncbi.nlm.nih.gov/nlmcatalog/journals), and are appropriately formatted and capitalised.

*Where website addresses are cited, please include the complete URL and specify the date of access (e.g. [accessed: 12/06/2023]).

*Please also see https://journals.plos.org/plosmedicine/s/submission-guidelines#loc-references for further details on reference formatting.

STUDY TYPE-SPECIFIC REQUESTS

The following list is derived from Geoffrey P Garnett, Simon Cousens, Timothy B Hallett, Richard Steketee, Neff Walker. Mathematical models in the evaluation of health programmes. (2011) Lancet DOI:10.1016/S0140-6736(10)61505-X:

*If pertinent, please provide a diagram that shows the model structure, including how the natural history of the disease is represented, the process and determinants of disease acquisition, and how the putative intervention could affect the system.

*Please provide a complete list of model parameters, including clear and precise descriptions of the meaning of each parameter, together with the values or ranges for each, with justification or the primary source cited and important caveats about the use of these values noted.

*Please provide a clear statement about how the model was fitted to the data, including goodness-of-fit measure, the numerical algorithm used, which parameter varied, constraints imposed on parameter values, and starting conditions.

*For uncertainty analyses, please state the sources of uncertainties quantified and not quantified [can include parameter, data, and model structure].

*Please provide sensitivity analyses to identify which parameter values are most important in the model. Uncertainty estimates seek to derive a range of credible results on the basis of an exploration of the range of reasonable parameter values. The choice of method should be presented and justified.

*Please discuss the scientific rationale for the choice of model structure and identify points where this choice could influence conclusions drawn. Please also describe the strength of the scientific basis underlying the key model assumptions.

*For studies that develop a prediction model or evaluate its performance, please ensure that the study is reported according to the TRIPOD statement (https://www.equator-network.org/reporting-guidelines/tripod-statement) and include the completed checklist as Supporting Information. Please add the following statement, or similar, to the Methods: "This study is reported as per the Transparent Reporting of a Multivariable Prediction Model for Individual Prognosis Or Diagnosis (TRIPOD) statement (S1 Checklist)." For studies using machine learning, please use the TRIPOD-AI checklist. When completing the checklist, please use section and paragraph numbers, rather than page numbers.

# Author comment: Author response to Decision Letter 2

**DOI:** 10.1371/journal.pmed.1005167.r003

5 Mar 2026

Attachment Submitted filename: Response to reviewers PLOSMed final.docx

# Peer review: Decision Letter 2

**DOI:** 10.1371/journal.pmed.1005167.r004

**Authors:** Suzanne De Bruijn

2 Jun 2026

Dear Dr Seferidi,

Many thanks for submitting a revised version of your manuscript "Retrofitting fiscal policies to reduce the double burden of malnutrition in Peru: a system dynamics modelling study" (PMEDICINE-D-25-04199R2) to PLOS Medicine. The paper has been seen again by two of the original reviewers; their comments are included below and can also be accessed here: [LINK]

As you will see, the reviewers thought the revision was adequate. However, the academic editor has a few remaining issues. After discussing the paper with the editorial team, as well as getting additional advice from the statistical reviewer, I'm pleased to invite you to revise the paper in response to these comments. I've included the comments from the AE, as well as some of the comments from the statistical reviewer below. We cannot provide any guarantees at this stage regarding publication.

In addition to these revisions, we have a few editorial requests we like you to address, which are included at the bottom of this email. Please provide a point-by-point response to these too. In addition to these revisions, you may need to complete some formatting changes, which you will receive in a follow up email. A member of our team will be in touch with a set of requests shortly. If you do not receive a separate email within a few days, please assume that checks have been completed, and no additional changes are required.

When you upload your revision, please include a point-by-point response that addresses all of the reviewer and editorial points, indicating the changes made in the manuscript and either an excerpt of the revised text or the location (eg: page and line number) where each change can be found. Please also be sure to check the general editorial comments at the end of this letter and include these in your point-by-point response. When you resubmit your paper, please include a clean version of the paper as the main article file and a version with changes tracked as a marked-up manuscript. It may also be helpful to check the guidelines for revised papers at http://journals.plos.org/plosmedicine/s/revising-your-manuscript for any that apply to your paper.

We ask that you submit your revision by Jun 23 2026 11:59PM. However, if this deadline is not feasible, please contact me by email, and we can discuss a suitable alternative.

Don't hesitate to contact me directly with any questions (sbruijn@plos.org).

Best regards,

Suzanne

Suzanne De Bruijn, PhD

Associate Editor

PLOS Medicine

sbruijn@plos.org

-----------------------------------------------------------

Comments from the academic editor:

1)I am not sure why they could not provide a clearer answer to the questions on cost-effectiveness using DALYs and also what mix of "double duty" interventions were considered (and the evidence of effectiveness thereof).

2)From my perspective, it is a bit naive to assume that revenues from sin taxes will only go to one sector, especially when interventions that address stunting are largely multisectoral (as shown in Ref 22). What isolated interventions for stunting reduction were modeled? Was breastfeeding considered a double duty intervention?

Statistical reviewer: Would it be possible to include reduced diarrheal disease burden as a double-duty intervention?

3)Finally, do they have any data on who is consuming SSBs and ultra-processed foods in Peru at the moment? Will the additional cash in Juntos 2 not go towards some of these items? What is the evidence that Juntos 1 has led to behavior change favoring healthy diets among the poor?""

Statistical reviewer: there was no consideration of a counterfactual scenario in which Juntos did not exist, which would need to be done to answer this question.

-----------------------------------------------------------

Comments from the reviewers:

Reviewer #1: Thank you for the responsive revision. My comments have been addressed. The detailed response to Reviewer 2 on DDAs, and the addition of the CLD, helped to address my concerns as well.

Reviewer #2: Thank you for including me in the group of reviewers for this interesting article. I believe that all the suggestions made were analysed and explained clearly. Congratulations on the article; I believe it will make a significant contribution to the fight against the double burden of malnutrition in Peru and in developing countries.

Any attachments provided with reviews can be seen via the following link: [LINK]

--------------------------------------------------------- ---

Editorial requests:

GENERAL EDITORIAL REQUESTS:

* Please confirm that your title complies with PLOS Medicine's style. Your title must be nondeclarative and not a question. It should begin with main concept if possible. "Effect of" should be used only if causality can be inferred, i.e., for an RCT. Please place the study design ("A randomized controlled trial," "A retrospective study," "A modelling study," etc.) in the subtitle (ie, after a colon).

* Please ensure that the Introduction ends with a clear description of the study question or hypothesis.

* Please ensure that all abbreviations are defined at first use throughout the text.

* Please confirm that all numbers presented in the abstract are present and identical to numbers presented in the main manuscript text.

* Please disclose any use of AI tools and technologies to the study or article content in the Methods, including the name(s) of any tool(s) used and a description of their use. Please see our guidelines for more information: https://journals.plos.org/plosmedicine/s/ethical-publishing-practice#loc-artificial-intelligence-tools-and-technologies.

* Statistical reporting: Please revise throughout the manuscript, including tables and figures. We appreciate that you do not have p-values, and it is fine to leave these out. However, please ensure that otherwise you adhere to the following style rules, including a comma instead of a hyphen between the 95% CI.

- Please report statistical information as follows to improve clarity for the reader ""22% (95% CI [13,28]; p</=)"".

- Please separate upper and lower bounds with commas instead of hyphens as the latter can be confused with reporting of negative values.

- Please repeat statistical definitions (HR, CI etc.) for each set of parentheses.

* Financial statement: please include URLs to the funders websites.

* Data availability: thank you for including this statement. In addition to the OSF link, can you also provide us with a DOI for the data?

ABSTRACT

* Please confirm that your abstract complies with our requirements, including format (three sections: Background, Methods and Findings, and Conclusions) and providing all the information relevant to this study type https://journals.plos.org/plosmedicine/s/submission-guidelines#loc-abstract

* In the last sentence of the Abstract Methods and Findings section, please describe the main limitation(s) of the study's methodology.

AUTHOR SUMMARY

* In the author summary, in the final bullet point of 'What Do These Findings Mean?', please include the main limitations of the study in non-technical language.

FIGURES AND TABLES

* Please define all abbreviations used in each figure/table (including those in Supporting Information files). (including SSB and UPF).

* Please ensure there is enough description of each of the figures in the legend. This includes describing what the grey areas and error bars mean (Figure 2 and Figure 4).

# Author comment: Author response to Decision Letter 3

**DOI:** 10.1371/journal.pmed.1005167.r005

10 Jun 2026

Attachment Submitted filename: Response to Reviewers PLOSMed R2.docx

# Editor report: Decision Letter 3

**DOI:** 10.1371/journal.pmed.1005167.r006

**Authors:** Suzanne De Bruijn

19 Jun 2026

Dear Dr Seferidi,

On behalf of my colleagues and the Academic Editor, Zulfiqar Bhutta, I am pleased to inform you that we have agreed to publish your manuscript "Retrofitting fiscal policies to reduce the double burden of malnutrition in Peru: a system dynamics modelling study" (PMEDICINE-D-25-04199R3) in PLOS Medicine.

Before your manuscript can be formally accepted, we have one remaining request:

-Thank you for stating that "AI tools were not used to generate any article content and were only used for minor editorial support (grammar, syntax, and clarity checks) in limited, selected parts of the text.". Can you please include this statement in the Methods section?

Furthermore, you will need to complete some formatting changes, which you will receive in a follow up email. Please be aware that it may take several days for you to receive this email; during this time no action is required by you. Once you have received these formatting requests, please note that your manuscript will not be scheduled for publication until you have made the required changes.

In the meantime, please log into Editorial Manager at http://www.editorialmanager.com/pmedicine/, click the "Update My Information" link at the top of the page, and update your user information to ensure an efficient production process.

PRESS

We frequently collaborate with press offices. If your institution or institutions have a press office, please notify them about your upcoming paper at this point, to enable them to help maximise its impact. If the press office is planning to promote your findings, we would be grateful if they could coordinate with medicinepress@plos.org. If you have not yet opted out of the early version process, we ask that you notify us immediately of any press plans so that we may do so on your behalf.

We also ask that you take this opportunity to read our Embargo Policy regarding the discussion, promotion and media coverage of work that is yet to be published by PLOS. As your manuscript is not yet published, it is bound by the conditions of our Embargo Policy. Please be aware that this policy is in place both to ensure that any press coverage of your article is fully substantiated and to provide a direct link between such coverage and the published work. For full details of our Embargo Policy, please visit http://www.plos.org/about/media-inquiries/embargo-policy/.

Thank you again for submitting to PLOS Medicine. We look forward to publishing your paper.

Sincerely,

Suzanne De Bruijn, PhD

Associate Editor

PLOS Medicine
